# Integrated bioinformatics analysis reveals novel key biomarkers and potential candidate small molecule drugs in gestational diabetes mellitus

**DOI:** 10.1042/BSR20210617

**Published:** 2021-05-21

**Authors:** Varun Alur, Varshita Raju, Basavaraj Vastrad, Anandkumar Tengli, Chanabasayya Vastrad, Shivakumar Kotturshetti

**Affiliations:** 1 Department of Endocrinology, J.J.M. Medical College, Davanagere, Karnataka 577004, India; 2 Department of Obstetrics and Gynecology, J.J.M. Medical College, Davanagere, Karnataka 577004, India; 3 Department of Biochemistry, Basaveshwar College of Pharmacy, Gadag, Karnataka 582103, India; 4 Department of Pharmaceutical Chemistry, JSS College of Pharmacy, Mysuru and JSS Academy of Higher Education and Research, Mysuru, Karnataka 570015, India; 5 Biostatistics and Bioinformatics, Chanabasava Nilaya, Bharthinagar, Dharwad, Karnataka 580001, India

**Keywords:** bioinformatics analysis, differentially expressed genes, gestational diabetes mellitus, novel biomarkers, small drug molecules

## Abstract

Gestational diabetes mellitus (GDM) is the metabolic disorder that appears during pregnancy. The current investigation aimed to identify central differentially expressed genes (DEGs) in GDM. The transcription profiling by array data (E-MTAB-6418) was obtained from the ArrayExpress database. The DEGs between GDM samples and non-GDM samples were analyzed. Functional enrichment analysis were performed using ToppGene. Then we constructed the protein–protein interaction (PPI) network of DEGs by the Search Tool for the Retrieval of Interacting Genes database (STRING) and module analysis was performed. Subsequently, we constructed the miRNA–hub gene network and TF–hub gene regulatory network. The validation of hub genes was performed through receiver operating characteristic curve (ROC). Finally, the candidate small molecules as potential drugs to treat GDM were predicted by using molecular docking. Through transcription profiling by array data, a total of 869 DEGs were detected including 439 up-regulated and 430 down-regulated genes. Functional enrichment analysis showed these DEGs were mainly enriched in reproduction, cell adhesion, cell surface interactions at the vascular wall and extracellular matrix organization. Ten genes, *HSP90AA1, EGFR, RPS13, RBX1, PAK1, FYN, ABL1, SMAD3, STAT3* and *PRKCA* were associated with GDM, according to ROC analysis. Finally, the most significant small molecules were predicted based on molecular docking. This investigation identified hub genes, signal pathways and therapeutic agents, which might help us, enhance our understanding of the mechanisms of GDM and find some novel therapeutic agents for GDM.

## Introduction

Gestational diabetes mellitus (GDM) is the metabolic disorder diagnosed during pregnancy, affecting 2–5% of pregnant women worldwide [1,2]. Risk factors of GDM include obesity, previous occurrence of diabetes, family history of type 2 diabetes, preeclampsia, hypertension, cardiovascular diseases and genetic factors [3]. At third trimester of pregnancy, blood glucose levels are drastically elevated [4]. Moreover, the elevated glucose level in pregnancy is closely linked with detrimental consequences in the newborn babies includes fetal hyperglycemia and cardiovascular disease [5]. Therefore, it is essential to examine the factual molecular targets included in occurrence and advancement of GDM, in order to make an improvement to the diagnosis, prognosis and treatment of GDM.

The molecular mechanisms of GDM initiation and development remain unclear. It is therefore essential to identify new genes and pathways that are linked with GDM progression and patient prognosis, which might not only help to explicate the underlying molecular mechanisms associated, but also to discover new diagnostic molecular markers and therapeutic targets. Transcription profiling by array can rapidly detect gene expression on a global basis and are particularly useful in screening for differentially expressed genes (DEGs) [6]. Gene chips allow the analysis of gene expression in a high-throughput way with great sensitivity, specificity and repeatability. A symbolic amount of data have been produced via the use of gene chips and the majority of such gene expression datasets have been uploaded and stored in public databases includes ArrayExpress database and NCBI‐Gene Expression Omnibus (NCBI‐GEO) database. Previous investigation concerning GDM transcription profiling by array have found hundreds of DEGs [7,8]. The availability of bioinformatics analysis based on high-throughput technology enabled the investigation of altered gene expression and the interaction between genes in GDM, to provide novel insights for further in-depth investigations.

In the current investigation, public transcription profiling by array data of E-MTAB-6418 from ArrayExpress database was downloaded. A total of 38 patients with GDM and 70 non-GDM candidates data in E-MTAB-6418 were available. DEGs between patients with GDM and non-GDM candidates were filtered and obtained using bioconductor package limma in R software. Gene Ontology (GO) and REACTOME pathway enrichment analyses of the DEGs were performed. The functions of the DEGs were further assessed by PPI network and module analyses to identify the hub genes in GDM. Subsequently, miRNA–hub gene regulatory network and TF–hub gene regulatory network were constructed and analyzed to find out the hub genes, miRNAs and TFs in GDM. Further, hub genes were validated by receiver operating characteristic curve (ROC) analysis and RT-PCR. Finally, a molecular docking study was performed for prediction of small drug molecules. Collectively, the findings of the current investigation highlighted hub genes and pathways that might contribute to the pathology of GDM. These might provide a basis for the advancement of future diagnostic, prognostic and therapeutic targets for GDM.

## Materials and methods

### Transcription profiling by array data information

The mRNA expression profile E-MTAB-6418 [9] based on A-MEXP-2072—Illumina HumanHT-12_V4_0_R2_15002873_B was downloaded from the ArrayExpress database (https://www.ebi.ac.uk/arrayexpress/) [10], which included 38 patients with GDM and 70 non-GDM candidates.

### Identification of DEGs

To obtain DEGs between GDM and non-GDM samples. After limma package in R analysis [11], results including adjusted *P*-values (adj. P. Val) and log FC were provided. Cut-off criterion was set as adj. P. Val <0.05, |log FC| > 1.158 for up-regulated genes and |log FC| < −0.83 for down-regulated genes. A list of candidate DEGs was obtained via the above methods.

### Gene ontology and pathway enrichment of DEGs analysis

Gene ontology (GO) analysis (http://geneontology.org/) [12] and REACTOME (https://reactome.org/) [13] pathway enrichment analysis were both integrated in the ToppGene (ToppFun) (https://toppgene.cchmc.org/enrichment.jsp) [14] program. Therefore, ToppGene was capable of providing comprehensive annotations for functional and pathway interpretations. In this experiment, DEGs were uploaded on to ToppGene in order to perform related GO and REACTOME pathway enrichment analyses. The cut-off criterion was set as *P*<0.05.

### PPI network establishment and modules selection

Search Tool for the Retrieval of Interacting Genes StringDB interactome (https://string-db.org/) is a database of known and predicted protein–protein interactions (PPIs) [15]. All candidate DEGs were posted into the STRING website, with a confidence score of ≥0.4 set as the cut-off criterion for PPI network construction. Then, Cytoscape (version 3.8.2, http://www.cytoscape.org/) [16] software was utilized to construct protein interaction relationship network. The Network Analyzer plugin was performed to scale node degree [17], betweenness centrality [18], stress centrality [19] and closeness centrality [20] of the PPI network. Significant modules in the visible PPI network were screened using the PEWCC1 (http://apps.cytoscape.org/apps/PEWCC1) [21] plugin. Degree cutoff = 2, node score cutoff = 0.2, k-core = 2 and max depth = 100 were set as the cut-off criterion. Three highest degree modules were extracted, and the potential mechanisms of each module were investigated with ToppGene. A degree of ≥10 was set as the filter criterion. Hub genes with high degree were selected as the potential key genes and biomarkers.

### miRNA–hub gene regulatory network construction

The miRNet database (https://www.mirnet.ca/) [22] is an open-source platform mainly focusing on miRNA–target interactions. miRNet utilizes 14 established miRNA–target prediction databases, including TarBase, miRTarBase, miRecords, miRanda, miR2Disease, HMDD, PhenomiR, SM2miR, PharmacomiR, EpimiR, starBase, TransmiR, ADmiRE and TAM 2.0. In the present study, miRNAs were considered the targeted miRNAs of hub genes. Subsequently, the network of the hub genes and their targeted miRNAs was visualized by Cytoscape software.

### TF–hub gene regulatory network construction

The NetworkAnalyst database (https://www.networkanalyst.ca/) [23] is an open-source platform mainly focusing on TF–target interactions. NetworkAnalyst utilizes three established TF–target prediction databases, including ENCODE, JASPAR, ChEA. In the present study, TFs were considered the targeted TFs of hub genes based on ChEA database. Subsequently, the network of the hub genes and their targeted TFs was visualized by Cytoscape software.

### Receiver operating characteristic curve analysis

The receiver operating characteristic curve (ROC) was constructed by predicting the probability of a diagnosis being of high or low integrated score of significant hub gene expression in GDM. Area under curve (AUC) analysis was operated to calculate the diagnostic ability by using the statistical package pROC in R software [24].

### RT-PCR analysis

The HTR­8/SVneo (ATCC CRL3271) cell line procured from ATCC. For normal HTR­8/SVneo (ATCC CRL3271) cell line was grown in RPMI-1640 medium added with 10% fetal bovine serum, containing 5.5 mM glucose, and 1% penicillin/streptomycin. This cell line was incubated at 37°C in a 5% CO_2_ in humidified cell culture incubator. Similarly, for GDM HTR­8/SVneo (ATCC CRL3271) cell line was grown in RPMI-1640 medium added with 10% fetal bovine serum, containing 5.5 mM glucose, and 1% penicillin/streptomycin. This cell line was incubated at 37°C in a 5% CO_2_ in humidified cell culture incubator for 24 h, then stimulated with various concentrations 40 mM of d-glucose for 6 h. TRIzol (cat. no. 9109; Takara Bio, Inc.) was used to isolate total RNA from HTR­8/SVneo cell line and HTR­8/SVneo cell line treated with glucose according to the manufacturer’s instructions. TRI Reagent (Sigma, U.S.A.). was used to isolate total RNA from each tissue sample according to the manufacturer’s instructions. Then, total RNA was reverse transcribed into cDNAs using the FastQuant RT kit (with gDNase; Tiangen Biotech Co., Ltd.). RT-PCR was performed to measure the levels of cDNAs using a QuantStudio 7 Flex real-time PCR system (Thermo Fisher Scientific, Waltham, MA, U.S.A.). RT-PCR procedure was performed as follows: pre-denaturation at 95°C for 30 s for 1 cycle followed by 40 cycles of 95°C for 5 s and 60°C for 20 s. The relative expression level of the hub genes was calculated following comparative *C*_T_ method [25]. β-actin was used to normalize the mRNA expression level. The primer sequences are listed in Table 1.

**Table 1:** The sequences of primers for quantitative RT-PCR

Genes	Forward primers	Reverse primers
*HSP90AA1*	AGGAGGTTGAGACGTTCGC	AGAGTTCGATCTTGTTTGTTCGG
*EGFR*	AGGCACGAGTAACAAGCTCAC	ATGAGGACATAACCAGCCACC
*RPS13*	TCCCAGTCGGCTTTACCCTAT	CAGGATTACACCGATCTGTGAAG
*RBX1*	TTGTGGTTGATAACTGTGCCAT	GACGCCTGGTTAGCTTGACAT
*PAK1*	CAGCCCCTCCGATGAGAAATA	CAAAACCGACATGAATTGTGTGT
*FYN*	ATGGGCTGTGTGCAATGTAAG	GAAGCTGGGGTAGTGCTGAG
*ABL1*	AAGCCGCTCGTTGGAACTC	AGACCCGGAGCTTTTCACCT
*SMAD3*	TGGACGCAGGTTCTCCAAAC	CCGGCTCGCAGTAGGTAAC
*STAT3*	CAGCAGCTTGACACACGGTA	AAACACCAAAGTGGCATGTGA
*PRKCA*	GTCCACAAGAGGTGCCATGAA	AAGGTGGGGCTTCCGTAAGT

### Molecular docking experiments

Molecular docking was used to find biologically active hits among the designed ligands. Using perpetual software module BIOVIA Discovery Studio (Perpetual), Surflex-Docking docking studies were conducted on active constituents. The lowest binding energy conformation was presumed to form a stable complex within the active site of the overexpressed proteins. The 2D structures were sketched using Chemdraw software, imported and saved into sdf. format using Open Babelfree software. The protein structure was processed after introduction of the protein, the co-crystallized ligand and all the water molecules were excluded from the crystal structure; more hydrogen was added and refined the side chain. The present study employed CDOCKER, a grid-based molecular docking approach that utilizes the CHARMm force field. A higher number indicates a stronger bond. The CDOCKER score is expressed as a negative number (–CDOCKER ENERGY). The H-bonds, van der Waals and electrostatic interactions between the target protein and the ligand were used to measure the CDOCKER energy. The modeled protein’s binding site was determined using the template protein’s crystal data and proteins which did not co-crystallize ligand generated binding site automatically. To make it easier for ligands to interact with amino acids, the binding site sphere center was set at 9 Å radius. Furthermore, using smart minimizer algorithm, CHARMm force field was applied followed by energy minimization to define local minima (lowest energy conformation) of the modeled over expressed proteins with an energy gradient of 0.1 kcal mol^−1^.Å^−1^, respectively. The energy minimized receptor protein and the set of 44 natural molecules which was reported as effective in diabetes mellitus and the well-known commonly used allopathic drugs, Metformin and Glyburide, were used as standard and to compare the binding interactions with natural molecules on overexpressed proteins in gestational diabetes. The binding site sphere radius set at *X* = 29.50, *Y* = −31.38 and *Z* = −38.79 were submitted to the CDOCKER parameter and also calculated binding energy. The X-ray co-crystallized structures were extracted from Protein Data Bank of PDB code of 4UV7, 5NJX, 3Q4Z and 3FNI of overexpressed genes of Epidermal growth factor receptor (EGFR), Heat shock protein 90 α family class A member 1 (HSP90AA1), P21 RAC1 activated kinase 1 (PAK1) and Ring-box 1 (RBX1), respectively, in gestational diabetes were selected for docking studies [26–29]. The best position was inserted into the molecular area between the protein and the ligand. The 2D and 3D interaction of amino acid molecules was achieved using the free online Discovery Studio Visualizer.

## Results

### Identification of DEGs

**Figure 1:**
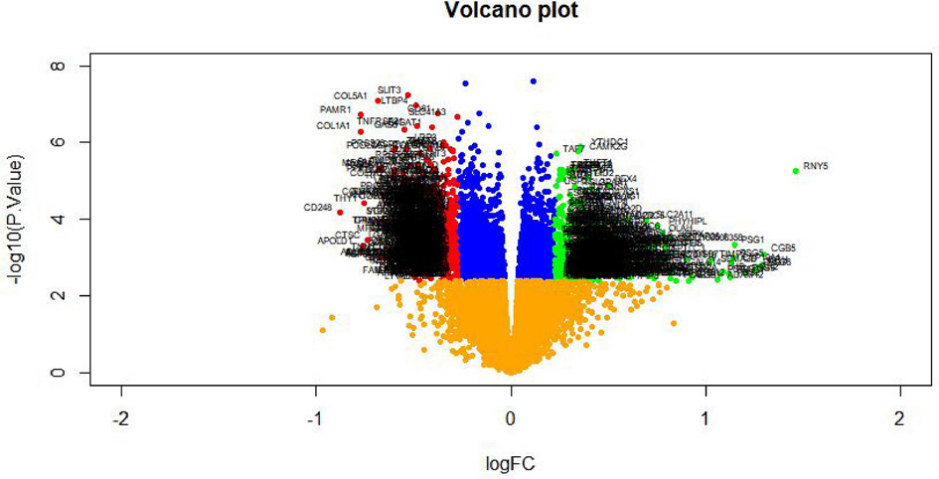
Volcano plot of DEGs Genes with a significant change of more than two-fold were selected. Green dot represented up-regulated significant genes and red dot represented down-regulated significant genes.

**Figure 2:**
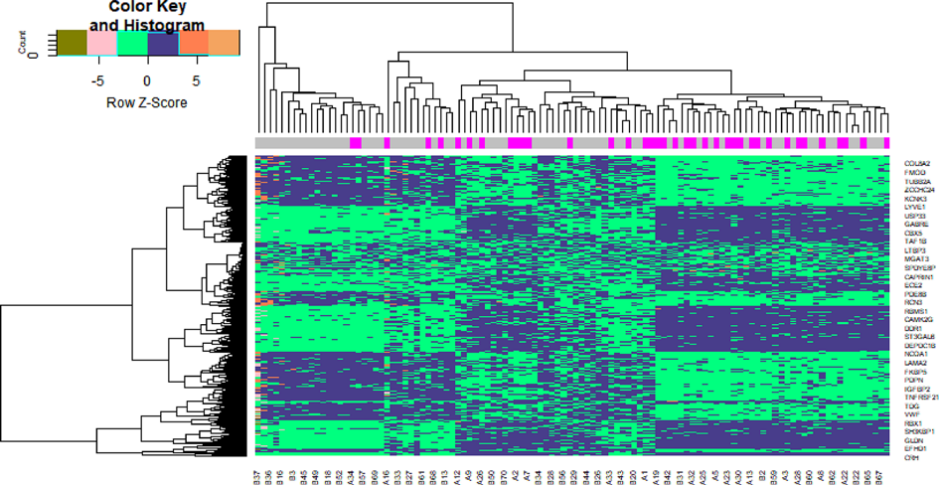
Heat map of DEGs Legend on the top left indicates log fold change of genes (A1– A38, GDM; B1–B70, GDM).

Transcription profiling by array datasets was obtained from the ArrayExpress database containing GDM and non-GDM samples; E-MTAB-6418. Then, the R package named ‘limma’ was processed for analysis with adjusted *P*<0.05, |log FC| > 1.158 for up-regulated genes and |log FC| < −0.83 for down-regulated genes. All DEGs were displayed in volcano maps (Figure 1). A total of 869 genes were finally obtained including 439 up-regulated and 430 down-regulated genes in the GDM samples compared with the non-GDM samples and are listed in Table 2. Top 869 genes in this dataset were displayed in the heatmap (Figure 2).

**Table 2:** The statistical metrics for key DEGs

IlluminaID	GeneSymbol	logFC	pValue	adj.P.Val	tvalue	Regulation	GeneName
ILMN_3246433	RNY5	1.462757	5.68E-06	0.002906	4.775349	Up	RNA, Ro60-associated Y5
ILMN_1691647	CGB5	1.297516	0.000781	0.021869	3.457767	Up	chorionic gonadotropin subunit β 5
ILMN_1668035	CRH	1.29002	0.001459	0.029715	3.266769	Up	corticotropin releasing hormone
ILMN_1716238	PSG6	1.284052	0.00171	0.032189	3.217109	Up	pregnancy specific β-1-glycoprotein 6
ILMN_1772768	PSG7	1.257768	0.001767	0.032784	3.206816	Up	pregnancy specific β-1-glycoprotein 7 (gene/pseudogene)
ILMN_2413473	GH2	1.248351	0.002276	0.037103	3.126124	Up	growth hormone 2
ILMN_1801776	PSG9	1.204077	0.002468	0.038662	3.100092	Up	pregnancy specific β-1-glycoprotein 9
ILMN_1798000	PSG1	1.147959	0.000469	0.017456	3.608336	Up	pregnancy specific β-1-glycoprotein 1
ILMN_1728734	PSG5	1.143624	0.000969	0.024168	3.392528	Up	pregnancy specific β-1-glycoprotein 5
ILMN_2387860	CYP19A1	1.130568	0.00132	0.028092	3.297762	Up	cytochrome P450 family 19 subfamily A member 1
ILMN_1764483	PSG2	1.123521	0.003218	0.044644	3.013539	Up	pregnancy specific β-1-glycoprotein 2
ILMN_1706911	PSG11	1.115865	0.002642	0.040078	3.07808	Up	pregnancy specific β-1-glycoprotein 11
ILMN_1765187	LHB	1.086528	0.002327	0.037529	3.118995	Up	luteinizing hormone subunit β
ILMN_1693397	PSG4	1.079921	0.002644	0.040091	3.077764	Up	pregnancy specific β-1-glycoprotein 4
ILMN_1785393	ADAM12	1.058143	0.003796	0.048669	2.958837	Up	ADAM metallopeptidase domain 12
ILMN_1749078	TIMP2	1.029031	0.001077	0.025325	3.360515	Up	TIMP metallopeptidase inhibitor 2
ILMN_2406299	SEMA3B	1.019814	0.001424	0.029234	3.274226	Up	semaphorin 3B
ILMN_1691937	CSH2	0.93195	0.003208	0.044568	3.014577	Up	chorionic somatomammotropin hormone 2
ILMN_2044645	CGB1	0.914238	0.003818	0.048801	2.956891	Up	chorionic gonadotropin subunit β 1
ILMN_2083578	CGB7	0.902725	0.001095	0.025576	3.355412	Up	chorionic gonadotropin subunit β 7
ILMN_1754207	PLAC1	0.846799	0.003832	0.048867	2.955674	Up	placenta enriched 1
ILMN_1698318	LGALS14	0.831948	0.001715	0.032189	3.216166	Up	galectin 14
ILMN_2068104	TFPI2	0.817524	0.003376	0.04562	2.997767	Up	tissue factor pathway inhibitor 2
ILMN_2316236	HOPX	0.817388	0.000335	0.014808	3.70535	Up	HOP homeobox
ILMN_1789638	MFSD2A	0.815456	0.002488	0.038799	3.097506	Up	major facilitator superfamily domain containing 2A
ILMN_1786908	KRTAP26-1	0.796216	0.00037	0.01533	3.677008	Up	keratin associated protein 26-1
ILMN_1659597	LOC100506358	0.792699	0.000395	0.015895	3.658029	Up	uncharacterized LOC100506358
ILMN_2118663	ERV3-1	0.791098	0.001056	0.025169	3.366462	Up	endogenous retrovirus group 3 member 1, envelope
ILMN_1712066	EXPH5	0.790876	0.000584	0.01904	3.543961	Up	exophilin 5
ILMN_1674696	OLAH	0.774415	0.00023	0.012505	3.811374	Up	oleoyl-ACP hydrolase
ILMN_2233454	SPTLC3	0.771167	0.000748	0.021335	3.470437	Up	serine palmitoyltransferase long chain base subunit 3
ILMN_1693530	PSG3	0.760751	0.002195	0.036289	3.137778	Up	pregnancy specific β-1-glycoprotein 3
ILMN_1784824	LINC01118	0.760468	0.001118	0.025812	3.349058	Up	long intergenic non-protein coding RNA 1118
ILMN_1813350	HSD11B2	0.758705	0.002101	0.035634	3.151814	Up	hydroxysteroid 11-β dehydrogenase 2
ILMN_2352921	BPGM	0.755678	0.000432	0.016748	3.631747	Up	bisphosphoglyceratemutase
ILMN_1678710	PHYHIPL	0.750771	0.000149	0.010164	3.932405	Up	phytanoyl-CoA 2-hydroxylase interacting protein like
ILMN_1794842	LGALS13	0.737107	0.002229	0.036621	3.132873	Up	galectin 13
ILMN_2188862	GDF15	0.733888	0.003343	0.045512	3.000989	Up	growth differentiation factor 15
ILMN_1702858	ADHFE1	0.733838	0.000566	0.018799	3.553236	Up	alcohol dehydrogenase iron containing 1
ILMN_2187746	EMX2	0.724034	0.002408	0.038098	3.108039	Up	empty spiracles homeobox 2
ILMN_1780693	HSD3B1	0.713165	0.003003	0.043117	3.03634	Up	hydroxy-δ-5-steroid dehydrogenase, 3 β- and steroid δ-isomerase 1
ILMN_1814737	LNPEP	0.699558	0.001327	0.028154	3.296246	Up	leucyl and cystinylaminopeptidase
ILMN_1807277	IFI30	0.696224	0.000433	0.01676	3.631088	Up	IFI30 lysosomalthiolreductase
ILMN_1756443	INHA	0.693534	0.000602	0.019237	3.535224	Up	inhibin subunit α
ILMN_1748090	SLC2A11	0.691618	0.000104	0.008561	4.029682	Up	solute carrier family 2 member 11
ILMN_1774287	CFB	0.689441	0.002619	0.039846	3.080829	Up	complement factor B
ILMN_1768662	UCK2	0.666106	0.00056	0.018724	3.556095	Up	uridine-cytidine kinase 2
ILMN_1720540	INSL4	0.662317	0.001009	0.024621	3.380194	Up	insulin like 4
ILMN_1797744	TPPP3	0.660409	0.000586	0.019062	3.543117	Up	tubulin polymerization promoting protein family member 3
ILMN_1680139	MAFF	0.655013	0.001413	0.02907	3.276627	Up	MAF bZIP transcription factor F
ILMN_2368188	TRPV6	0.647442	0.001158	0.02618	3.338062	Up	transient receptor potential cation channel subfamily V member 6
ILMN_1740466	TENT5A	0.645318	0.000443	0.016957	3.624672	Up	terminal nucleotidyltransferase 5A
ILMN_1800412	BMP1	0.634772	0.000459	0.017315	3.614562	Up	bone morphogenetic protein 1
ILMN_1727633	NECTIN3	0.633198	0.002322	0.037503	3.11981	Up	nectin cell adhesion molecule 3
ILMN_1664855	PPP1R14C	0.629794	0.001066	0.025246	3.363473	Up	protein phosphatase 1 regulatory inhibitor subunit 14C
ILMN_1695562	ZNF471	0.623497	0.000993	0.024402	3.38509	Up	zinc finger protein 471
ILMN_1714586	VGLL3	0.618471	0.001854	0.033507	3.191522	Up	vestigial like family member 3
ILMN_1744949	RHOBTB3	0.61326	0.001612	0.031272	3.235567	Up	Rho related BTB domain containing 3
ILMN_1703284	SPIRE2	0.612933	0.00328	0.045007	3.007246	Up	spire type actin nucleation factor 2
ILMN_1704376	GLDN	0.605249	0.002329	0.037531	3.118817	Up	gliomedin
ILMN_2415421	SLC30A2	0.602925	0.002569	0.039356	3.087159	Up	solute carrier family 30 member 2
ILMN_1757406	H1-2	0.598625	0.001243	0.027166	3.316284	Up	H1.2 linker histone, cluster member
ILMN_1651496	H2BC5	0.597413	0.000105	0.008597	4.027633	Up	H2B clustered histone 5
ILMN_1773125	ENTPD1	0.596924	0.003282	0.045007	3.007041	Up	ectonucleoside triphosphate diphosphohydrolase 1
ILMN_1790228	FURIN	0.595716	0.001344	0.028402	3.292157	Up	furin, paired basic amino acid cleaving enzyme
ILMN_1741143	TXK	0.593669	0.001359	0.028533	3.288903	Up	TXK tyrosine kinase
ILMN_1787750	CD200	0.592992	0.001168	0.02622	3.335461	Up	CD200 molecule
ILMN_1795106	PSG8	0.59239	0.002853	0.041916	3.053083	Up	pregnancy specific β-1-glycoprotein 8
ILMN_1672908	TWIST1	0.585791	0.002193	0.036269	3.138064	Up	twist family bHLH transcription factor 1
ILMN_1787691	CITED4	0.583851	0.000456	0.017275	3.616082	Up	Cbp/p300 interacting transactivator with Glu/Asp rich carboxy-terminal domain 4
ILMN_1740917	SCNN1B	0.580064	0.00208	0.035516	3.155039	Up	sodium channel epithelial 1 β subunit
ILMN_1681248	TCHH	0.579544	0.000868	0.023007	3.42568	Up	trichohyalin
ILMN_1713397	NCCRP1	0.577054	0.001607	0.031251	3.236641	Up	NCCRP1, F-box associated domain containing
ILMN_1771019	MTMR4	0.575772	0.000998	0.024462	3.383725	Up	myotubularin related protein 4
ILMN_1792689	H2AC6	0.572844	0.000198	0.011571	3.853384	Up	H2A clustered histone 6
ILMN_1732071	H2BC21	0.571255	0.000494	0.01788	3.593188	Up	H2B clustered histone 21
ILMN_1777934	MORN3	0.570881	0.000392	0.015793	3.660351	Up	MORN repeat containing 3
ILMN_1754126	SH2D5	0.567064	0.000548	0.018452	3.562897	Up	SH2 domain containing 5
ILMN_1768820	CYP11A1	0.562921	0.002281	0.037107	3.125417	Up	cytochrome P450 family 11 subfamily A member 1
ILMN_1721842	RYBP	0.560609	0.001133	0.026002	3.344898	Up	RING1 and YY1 binding protein
ILMN_2323172	CSF3R	0.55608	0.002173	0.03615	3.140995	Up	colony stimulating factor 3 receptor
ILMN_1693789	ALPP	0.554751	0.003332	0.045427	3.002056	Up	alkaline phosphatase, placental
ILMN_2129015	AFF1	0.5529	0.003097	0.043799	3.026214	Up	AF4/FMR2 family member 1
ILMN_1807652	STRA6	0.548925	0.001375	0.028697	3.285192	Up	stimulated by retinoic acid 6
ILMN_1746517	KYNU	0.547309	0.002021	0.035003	3.164234	Up	kynureninase
ILMN_1793695	ITIH5	0.543721	0.002744	0.041015	3.065788	Up	inter-α-trypsin inhibitor heavy chain 5
ILMN_1814600	DEPDC1B	0.542445	0.001287	0.027725	3.305687	Up	DEP domain containing 1B
ILMN_1708340	DAPK1	0.541376	0.003167	0.04426	3.018827	Up	death associated protein kinase 1
ILMN_2204545	ST3GAL4	0.537233	0.001733	0.032379	3.212881	Up	ST3 β-galactoside α-2,3-sialyltransferase 4
ILMN_1794239	ODAPH	0.533324	0.000498	0.017895	3.590399	Up	odontogenesis associated phosphoprotein
ILMN_2315780	TACC2	0.532522	0.000106	0.00868	4.024113	Up	transforming acidic coiled-coil containing protein 2
ILMN_2309446	RBBP6	0.528104	0.000254	0.013135	3.783862	Up	RB binding protein 6, ubiquitin ligase
ILMN_1791545	KRT23	0.527971	0.000209	0.011919	3.838417	Up	keratin 23
ILMN_1798458	KAZN	0.51384	0.001255	0.027297	3.313396	Up	kazrin, periplakin interacting protein
ILMN_1777683	ADAMTSL4	0.513545	0.00031	0.014326	3.727088	Up	ADAMTS like 4
ILMN_1811593	NIPAL1	0.509377	0.000573	0.018927	3.549631	Up	NIPA like domain containing 1
ILMN_3236821	HSPB1	0.507177	0.000829	0.022503	3.439718	Up	heat shock protein family B (small) member 1
ILMN_1774229	SLC7A4	0.504867	0.003047	0.043409	3.031496	Up	solute carrier family 7 member 4
ILMN_1795838	C4orf19	0.503581	0.000615	0.019397	3.528742	Up	chromosome 4 open reading frame 19
ILMN_1689004	TNFRSF12A	0.50273	0.000813	0.022284	3.44546	Up	TNF receptor superfamily member 12A
ILMN_1702105	EFS	0.502312	0.001688	0.032021	3.221088	Up	embryonal Fyn-associated substrate
ILMN_1725831	TINCR	0.502082	0.001143	0.026058	3.342141	Up	TINCR ubiquitin domain containing
ILMN_1726597	RIPOR2	0.501684	0.000791	0.022033	3.453621	Up	RHO family interacting cell polarization regulator 2
ILMN_1746618	PAQR7	0.498553	0.000293	0.014028	3.743357	Up	progestin and adipoQ receptor family member 7
ILMN_2351638	BEX4	0.49812	1.22E-05	0.003404	4.586051	Up	brain expressed X-linked 4
ILMN_1762207	SGSM1	0.495004	0.000544	0.018404	3.565008	Up	small G protein signaling modulator 1
ILMN_1802690	GULP1	0.492328	0.002712	0.040738	3.069508	Up	GULP PTB domain containing engulfment adaptor 1
ILMN_1679041	SLC3A2	0.486676	0.001058	0.025186	3.365946	Up	solute carrier family 3 member 2
ILMN_1728677	CREB5	0.486008	0.000136	0.009772	3.957019	Up	cAMP responsive element binding protein 5
ILMN_2390609	ANK3	0.481205	0.001627	0.031404	3.232619	Up	ankyrin 3
ILMN_1740170	CHCHD10	0.479287	0.002374	0.03792	3.11261	Up	coiled-coil-helix-coiled-coil-helix domain containing 10
ILMN_1813139	ANKDD1A	0.477831	0.000338	0.014847	3.702751	Up	ankyrin repeat and death domain containing 1A
ILMN_2194448	STT3B	0.477537	0.00228	0.037103	3.125635	Up	STT3 oligosaccharyltransferase complex catalytic subunit B
ILMN_2079991	ERVW-1	0.468734	0.000211	0.01196	3.835811	Up	endogenous retrovirus group W member 1, envelope
ILMN_1684034	STAT5B	0.466134	0.000495	0.01788	3.59265	Up	signal transducer and activator of transcription 5B
ILMN_1796423	CLIC3	0.465597	0.001265	0.027404	3.31097	Up	chloride intracellular channel 3
ILMN_3280402	GLRX	0.464843	0.000542	0.018404	3.56612	Up	glutaredoxin
ILMN_1753931	CDO1	0.464443	0.000182	0.011037	3.877815	Up	cysteine dioxygenase type 1
ILMN_2065690	GRAMD2B	0.464402	0.000499	0.017901	3.589918	Up	GRAM domain containing 2B
ILMN_1752510	FAM13A	0.463446	0.000335	0.014808	3.705566	Up	family with sequence similarity 13 member A
ILMN_2384857	DHRS2	0.460744	0.001634	0.031474	3.23133	Up	dehydrogenase/reductase 2
ILMN_1720771	STX11	0.459749	0.002149	0.035892	3.144568	Up	syntaxin 11
ILMN_1807563	FKBP2	0.457573	0.000724	0.02097	3.480237	Up	FKBP prolylisomerase 2
ILMN_1669557	CRYBG2	0.4569	0.001328	0.028154	3.296056	Up	crystallin β-γ domain containing 2
ILMN_1699206	FHDC1	0.455751	0.00209	0.035562	3.153528	Up	FH2 domain containing 1
ILMN_1806149	C16orf74	0.455006	0.000426	0.016582	3.636053	Up	chromosome 16 open reading frame 74
ILMN_1751120	H4C8	0.45076	0.000764	0.021605	3.464112	Up	H4 clustered histone 8
ILMN_1740604	RAB11FIP5	0.450629	0.003974	0.049778	2.943476	Up	RAB11 family interacting protein 5
ILMN_3195497	ADIRF-AS1	0.448015	0.002142	0.035854	3.145598	Up	ADIRF antisense RNA 1
ILMN_1813625	TRIM25	0.445493	0.000114	0.00893	4.005574	Up	tripartite motif containing 25
ILMN_1753515	SRR	0.44366	0.003251	0.044884	3.010168	Up	serine racemase
ILMN_1772627	NSG1	0.441643	0.001427	0.029273	3.273602	Up	neuronal vesicle trafficking associated 1
ILMN_2364700	ENSA	0.441237	1.88E-05	0.004187	4.477906	Up	endosulfine α
ILMN_1674243	TFRC	0.43767	0.003549	0.046989	2.981226	Up	transferrin receptor
ILMN_1779448	EFHD1	0.435614	0.003381	0.045635	2.997287	Up	EF-hand domain family member D1
ILMN_1798975	EGFR	0.434921	0.002718	0.040787	3.068785	Up	epidermal growth factor receptor
ILMN_1802053	ZNF91	0.433844	0.000914	0.023507	3.410338	Up	zinc finger protein 91
ILMN_1797557	PLEKHA6	0.43335	0.003538	0.046906	2.982198	Up	pleckstrin homology domain containing A6
ILMN_1814333	SERPINI1	0.433318	0.00355	0.046989	2.981082	Up	serpin family I member 1
ILMN_1683211	NCAN	0.430909	0.002311	0.037392	3.121301	Up	neurocan
ILMN_2142353	GRTP1	0.430742	0.001078	0.025332	3.360135	Up	growth hormone regulated TBC protein 1
ILMN_1809477	CARHSP1	0.428795	0.001041	0.024977	3.370793	Up	calcium regulated heat stable protein 1
ILMN_1767365	PAK1	0.427899	0.000143	0.009932	3.94428	Up	p21 (RAC1) activated kinase 1
ILMN_1759792	CLIP4	0.427572	0.000478	0.017642	3.60267	Up	CAP-Gly domain containing linker protein family member 4
ILMN_2143685	CLDN7	0.426872	0.000634	0.019679	3.519853	Up	claudin 7
ILMN_2074860	RN7SK	0.425278	0.000506	0.017919	3.586106	Up	RNA component of 7SK nuclear ribonucleoprotein
ILMN_1742538	PCDHGC4	0.422624	0.00088	0.023184	3.421715	Up	protocadherin γ subfamily C, 4
ILMN_1689817	LCOR	0.419011	0.001391	0.028854	3.281687	Up	ligand dependent nuclear receptor corepressor
ILMN_1667994	AMD1	0.418735	0.00061	0.019374	3.531034	Up	adenosylmethionine decarboxylase 1
ILMN_1683598	ACSL4	0.416616	0.003954	0.04961	2.945175	Up	acyl-CoA synthetase long chain family member 4
ILMN_1796206	KMT2C	0.415266	8.27E-05	0.007525	4.092148	Up	lysine methyltransferase 2C
ILMN_1729417	GNE	0.413507	0.001169	0.02622	3.335407	Up	glucosamine (UDP-N-acetyl)-2-epimerase/N-acetylmannosamine kinase
ILMN_1778956	STS	0.411932	0.000347	0.014977	3.695049	Up	steroid sulfatase
ILMN_2405254	GRB7	0.408773	0.00026	0.013186	3.777315	Up	growth factor receptor bound protein 7
ILMN_1813314	H2BC12	0.408761	0.002651	0.04015	3.076962	Up	H2B clustered histone 12
ILMN_2346339	FOLR1	0.407865	0.000266	0.013333	3.771078	Up	folate receptor α
ILMN_1747112	GPAA1	0.407772	1.5E-05	0.003692	4.534163	Up	glycosylphosphatidylinositol anchor attachment 1
ILMN_1736863	TMEM140	0.40612	0.000597	0.019237	3.537542	Up	transmembrane protein 140
ILMN_3226388	PSG10P	0.399644	0.00336	0.045553	2.999338	Up	pregnancy specific β-1-glycoprotein 10, pseudogene
ILMN_1769092	EVA1B	0.398688	0.002925	0.042546	3.044952	Up	eva-1 homolog B
ILMN_1654322	ATP1B3	0.398471	0.00148	0.029861	3.262353	Up	ATPase Na+/K+ transporting subunit β 3
ILMN_1699674	ZNF703	0.397878	0.003462	0.046339	2.989443	Up	zinc finger protein 703
ILMN_2159730	GABRB1	0.396679	0.000663	0.020103	3.506538	Up	γ-aminobutyric acid type A receptor β1 subunit
ILMN_2342437	KLHL5	0.395369	0.003031	0.043318	3.033257	Up	kelch like family member 5
ILMN_1704472	EID2	0.394584	8.47E-06	0.00295	4.677314	Up	EP300 interacting inhibitor of differentiation 2
ILMN_2374865	ATF3	0.394537	0.001939	0.034245	3.177337	Up	activating transcription factor 3
ILMN_1652540	RELL2	0.39244	0.00081	0.022284	3.446717	Up	RELT like 2
ILMN_1697642	BCAP29	0.391558	0.000201	0.011654	3.849685	Up	B cell receptor associated protein 29
ILMN_2382974	CCDC7	0.391544	0.000891	0.023304	3.4178	Up	coiled-coil domain containing 7
ILMN_1742260	ITPRID2	0.39091	0.000176	0.010925	3.886377	Up	ITPR interacting domain containing 2
ILMN_2060145	GRHL2	0.389706	0.000397	0.015954	3.656341	Up	grainyhead like transcription factor 2
ILMN_2195821	CREBRF	0.389024	0.000261	0.013186	3.77657	Up	CREB3 regulatory factor
ILMN_1746676	CLDN8	0.388757	0.002765	0.041192	3.063276	Up	claudin 8
ILMN_1700583	ZNF750	0.388452	0.000505	0.017918	3.586783	Up	zinc finger protein 750
ILMN_1655913	NUCB2	0.386679	0.002616	0.039837	3.081247	Up	nucleobindin 2
ILMN_1701393	TBX3	0.381209	1.9E-05	0.004198	4.474884	Up	T-box transcription factor 3
ILMN_1769201	ELF3	0.380808	0.002808	0.041529	3.058207	Up	E74 like ETS transcription factor 3
ILMN_1791280	HSPB8	0.380526	0.002023	0.03504	3.163783	Up	heat shock protein family B (small) member 8
ILMN_2149292	TMEM40	0.378498	0.000676	0.020287	3.500728	Up	transmembrane protein 40
ILMN_1707088	DENND2D	0.37794	7.09E-05	0.007061	4.133296	Up	DENN domain containing 2D
ILMN_2179778	PHLDB2	0.377513	0.000242	0.012762	3.797837	Up	pleckstrin homology like domain family B member 2
ILMN_1801216	S100P	0.375994	0.00042	0.016421	3.640526	Up	S100 calcium binding protein P
ILMN_1699254	PLEKHH1	0.374597	0.000398	0.015954	3.656068	Up	pleckstrin homology, MyTH4 and FERM domain containing H1
ILMN_1710954	FBXL19-AS1	0.37328	0.000271	0.013483	3.765506	Up	FBXL19 antisense RNA 1
ILMN_2376502	RHOBTB1	0.372696	0.001027	0.024813	3.374907	Up	Rho related BTB domain containing 1
ILMN_1673455	RASAL2	0.372204	6.16E-05	0.006575	4.170741	Up	RAS protein activator like 2
ILMN_3194638	EVA1A	0.371837	0.001826	0.033257	3.196396	Up	eva-1 homolog A, regulator of programmed cell death
ILMN_1710284	HES1	0.370834	0.000842	0.022673	3.435074	Up	hes family bHLH transcription factor 1
ILMN_2064655	CXorf40A	0.369875	3.68E-05	0.005267	4.305465	Up	chromosome X open reading frame 40A
ILMN_2373566	PJA1	0.365283	0.001767	0.032784	3.206709	Up	praja ring finger ubiquitin ligase 1
ILMN_1779648	H2AW	0.365117	0.002673	0.040354	3.074252	Up	H2A.W histone
ILMN_2333107	TLE5	0.363987	0.003586	0.047223	2.977803	Up	TLE family member 5, transcriptional modulator
ILMN_1722025	CPEB4	0.363264	0.000601	0.019237	3.535255	Up	cytoplasmic polyadenylation element binding protein 4
ILMN_1670263	CNST	0.362857	0.001654	0.031635	3.227556	Up	consortin, connexin sorting protein
ILMN_2214678	MXD1	0.36052	0.003377	0.04562	2.997692	Up	MAX dimerization protein 1
ILMN_2324202	GABRE	0.359786	0.001796	0.032966	3.20158	Up	γ-aminobutyric acid type A receptor epsilon subunit
ILMN_2049727	OSER1	0.358697	0.000322	0.014545	3.716924	Up	oxidative stress responsive serine rich 1
ILMN_1704377	USP27X	0.35826	0.00113	0.025976	3.34568	Up	ubiquitin specific peptidase 27 X-linked
ILMN_3233388	RELL1	0.357964	0.002177	0.036172	3.1404	Up	RELT like 1
ILMN_1670878	YTHDC1	0.357534	1.29E-06	0.001955	5.128971	Up	YTH domain containing 1
ILMN_1815445	IDS	0.356888	0.002505	0.038937	3.095289	Up	iduronate 2-sulfatase
ILMN_1775448	PFN2	0.353131	0.000871	0.023045	3.424684	Up	profilin 2
ILMN_1657423	SPG21	0.353073	0.000213	0.011986	3.833747	Up	SPG21 abhydrolase domain containing, maspardin
ILMN_2162799	AHR	0.353025	0.002516	0.039016	3.093903	Up	aryl hydrocarbon receptor
ILMN_1698323	PLEKHB2	0.352741	0.00209	0.035562	3.153476	Up	pleckstrin homology domain containing B2
ILMN_1725718	ZSCAN4	0.352414	0.000589	0.019114	3.541628	Up	zinc finger and SCAN domain containing 4
ILMN_2414325	TNFAIP8	0.351941	4.64E-05	0.005784	4.245233	Up	TNF α induced protein 8
ILMN_1656291	TSKS	0.350101	3.27E-05	0.005189	4.336503	Up	testis specific serine kinase substrate
ILMN_3245236	FBRS	0.349549	0.002921	0.042546	3.045346	Up	fibrosin
ILMN_3243972	SNORA70B	0.349376	0.00036	0.015127	3.684829	Up	small nucleolar RNA, H/ACA box 70B
ILMN_1687519	SNAP23	0.349045	0.000691	0.020442	3.494035	Up	synaptosome associated protein 23
ILMN_3307729	CXXC5	0.347435	0.003855	0.04893	2.953691	Up	CXXC finger protein 5
ILMN_2359601	CAMK2G	0.346831	1.67E-06	0.001955	5.068099	Up	calcium/calmodulin dependent protein kinase II γ
ILMN_2358541	RBMS1	0.346578	0.001495	0.030009	3.259102	Up	RNA binding motif single stranded interacting protein 1
ILMN_1812262	DDR1	0.345856	0.001274	0.027548	3.308776	Up	discoidin domain receptor tyrosine kinase 1
ILMN_1655702	ABHD5	0.34552	0.000199	0.011583	3.852742	Up	abhydrolase domain containing 5
ILMN_1730294	INO80C	0.345306	0.000584	0.01904	3.543858	Up	INO80 complex subunit C
ILMN_1729095	PDZD2	0.34383	0.000816	0.022314	3.444388	Up	PDZ domain containing 2
ILMN_1775405	ARL4A	0.3433	0.000509	0.017937	3.584468	Up	ADP ribosylation factor like GTPase 4A
ILMN_1680937	H2BC4	0.342683	0.003302	0.045124	3.005054	Up	H2B clustered histone 4
ILMN_1689578	TLR3	0.342449	0.002261	0.036968	3.128251	Up	toll like receptor 3
ILMN_2278335	AKR1B15	0.342114	0.001891	0.033788	3.185343	Up	aldo-ketoreductase family 1 member B15
ILMN_1721922	NAB2	0.340891	0.00057	0.018862	3.551119	Up	NGFI-A binding protein 2
ILMN_1691237	CAP2	0.339551	0.00234	0.037639	3.117244	Up	cyclase associated actin cytoskeleton regulatory protein 2
ILMN_2395389	PSMC4	0.336399	0.000477	0.017642	3.602967	Up	proteasome 26S subunit, ATPase 4
ILMN_2173919	MYO9A	0.33636	0.003286	0.045007	3.006655	Up	myosin IXA
ILMN_1661809	PRRG4	0.336227	0.00211	0.035643	3.150489	Up	proline rich and Gla domain 4
ILMN_2307455	UBE2A	0.334363	0.001495	0.030009	3.259139	Up	ubiquitin conjugating enzyme E2 A
ILMN_3307700	SPCS3	0.333342	0.002825	0.041676	3.056249	Up	signal peptidase complex subunit 3
ILMN_1654370	TESK2	0.333053	3.57E-05	0.005231	4.313565	Up	testis associated actin remodelling kinase 2
ILMN_1742824	SPATA13	0.331307	0.000114	0.008925	4.006357	Up	spermatogenesis associated 13
ILMN_1688755	AAK1	0.329844	7.89E-05	0.007387	4.104652	Up	AP2 associated kinase 1
ILMN_1781374	TUFT1	0.328884	4.48E-06	0.002802	4.832739	Up	tuftelin 1
ILMN_2124386	RGL2	0.327869	1.43E-05	0.003559	4.547271	Up	ral guanine nucleotide dissociation stimulator like 2
ILMN_1803939	YIPF6	0.327011	0.000892	0.023304	3.417647	Up	Yip1 domain family member 6
ILMN_2170949	SNX10	0.326909	0.002527	0.039088	3.092476	Up	sorting nexin 10
ILMN_1775304	DNAJB1	0.326714	0.001071	0.025282	3.362025	Up	DnaJ heat shock protein family (Hsp40) member B1
ILMN_1657515	RPS6KA5	0.32621	0.003592	0.047263	2.977249	Up	ribosomal protein S6 kinase A5
ILMN_1690826	TNKS1BP1	0.321786	0.001116	0.025804	3.349443	Up	tankyrase 1 binding protein 1
ILMN_1814002	TEAD3	0.320268	7.41E-05	0.007229	4.121364	Up	TEA domain transcription factor 3
ILMN_1768958	RASGRP1	0.31925	0.003686	0.047951	2.968625	Up	RAS guanyl releasing protein 1
ILMN_2077623	RRAS2	0.319214	0.001037	0.024918	3.371789	Up	RAS related 2
ILMN_1693014	CEBPB	0.318883	0.002995	0.043063	3.037146	Up	CCAAT enhancer binding protein β
ILMN_3235340	ACER2	0.318499	3.54E-05	0.005231	4.315633	Up	alkaline ceramidase 2
ILMN_2403458	SMARCB1	0.318053	0.002694	0.040563	3.071724	Up	SWI/SNF related, matrix associated, actin dependent regulator of chromatin, subfamily b, member 1
ILMN_1805395	LTBP3	0.317611	0.000756	0.021442	3.467486	Up	latent transforming growth factor β binding protein 3
ILMN_1804148	TMED4	0.317228	0.001133	0.026002	3.344761	Up	transmembrane p24 trafficking protein 4
ILMN_1702447	IGF2BP2	0.316952	0.000692	0.020442	3.493585	Up	insulin like growth factor 2 mRNA binding protein 2
ILMN_1717195	MBD2	0.316258	0.000121	0.009233	3.98994	Up	methyl-CpG binding domain protein 2
ILMN_1747451	PLCXD1	0.316197	3.44E-05	0.005189	4.323024	Up	phosphatidylinositol specific phospholipase C X domain containing 1
ILMN_1777439	TCL6	0.313824	4.13E-05	0.005607	4.275901	Up	T cell leukemia/lymphoma 6
ILMN_2358457	ATF4	0.312963	5E-06	0.002879	4.806336	Up	activating transcription factor 4
ILMN_1694233	ACYP1	0.312484	8.05E-06	0.00295	4.689698	Up	acylphosphatase 1
ILMN_1675937	ANKRD9	0.312142	0.000113	0.008909	4.008887	Up	ankyrin repeat domain 9
ILMN_1670304	FAM156A	0.311579	0.002344	0.03767	3.116659	Up	family with sequence similarity 156 member A
ILMN_1717234	CAST	0.310235	0.000968	0.024163	3.392908	Up	calpastatin
ILMN_1710136	PUDP	0.309696	0.000391	0.015793	3.661101	Up	pseudouridine 5′-phosphatase
ILMN_1750969	FAM120AOS	0.309171	0.001969	0.034468	3.172477	Up	family with sequence similarity 120A opposite strand
ILMN_1717046	MOB3B	0.309072	0.001014	0.024678	3.378711	Up	MOB kinase activator 3B
ILMN_1684042	BET1	0.307537	0.000168	0.010659	3.898957	Up	Bet1 golgi vesicular membrane trafficking protein
ILMN_1664303	HTATIP2	0.306236	0.00063	0.019629	3.521367	Up	HIV-1 Tat interactive protein 2
ILMN_3263225	CRIM1-DT	0.305254	0.000552	0.018547	3.560684	Up	CRIM1 divergent transcript
ILMN_1763127	ACKR2	0.305224	0.001928	0.03416	3.179209	Up	atypical chemokine receptor 2
ILMN_1708611	RDX	0.30478	0.001592	0.031099	3.239468	Up	radixin
ILMN_2190414	ZNF83	0.304639	0.003774	0.048525	2.960719	Up	zinc finger protein 83
ILMN_3184978	ST8SIA6-AS1	0.304228	3.46E-05	0.005189	4.321517	Up	ST8SIA6 antisense RNA 1
ILMN_1746494	FNTA	0.303227	2.43E-05	0.00477	4.412621	Up	farnesyltransferase, CAAX box, α
ILMN_3238854	RGPD8	0.302737	0.000139	0.00984	3.950626	Up	RANBP2 like and GRIP domain containing 8
ILMN_2322498	RORA	0.302665	5.91E-05	0.006473	4.181545	Up	RAR related orphan receptor A
ILMN_2181892	BEX2	0.301932	0.000691	0.020442	3.494243	Up	brain expressed X-linked 2
ILMN_1716988	OPN3	0.300318	0.002185	0.036205	3.139321	Up	opsin 3
ILMN_1780382	SPCS2P4	0.299459	0.003743	0.048256	2.963549	Up	signal peptidase complex subunit 2 pseudogene 4
ILMN_1782685	DDB1	0.298269	0.003647	0.047663	2.972203	Up	damage specific DNA binding protein 1
ILMN_1801020	ADK	0.298209	0.000201	0.011664	3.849125	Up	adenosine kinase
ILMN_1653793	PDPK1	0.297954	0.002793	0.041395	3.059925	Up	3-phosphoinositide dependent protein kinase 1
ILMN_1805225	LPCAT3	0.296395	6.38E-06	0.00295	4.746876	Up	lysophosphatidylcholineacyltransferase 3
ILMN_1741371	PGAP6	0.296064	0.003279	0.045007	3.007377	Up	post-glycosylphosphatidylinositol attachment to proteins 6
ILMN_2187718	COX17	0.295972	0.000244	0.01284	3.795442	Up	cytochrome c oxidase copper chaperone COX17
ILMN_2263466	ACADVL	0.295932	0.000784	0.021918	3.456584	Up	acyl-CoA dehydrogenase very long chain
ILMN_1687947	H2BC6	0.295728	0.000669	0.020173	3.503643	Up	H2B clustered histone 6
ILMN_1723843	CSNK2A2	0.294128	0.002683	0.040473	3.073056	Up	casein kinase 2 α 2
ILMN_1662578	C1GALT1	0.294084	0.00014	0.00984	3.949908	Up	core 1 synthase, glycoprotein-N-acetylgalactosamine 3-β-galactosyltransferase 1
ILMN_1807423	IGF2BP3	0.293802	0.000826	0.02247	3.440691	Up	insulin like growth factor 2 mRNA binding protein 3
ILMN_3204734	STAG3L5P-PVRIG2P-PILRB	0.293124	0.003136	0.044111	3.022076	Up	STAG3L5P-PVRIG2P-PILRB readthrough
ILMN_1754145	CAPRIN1	0.293108	0.00032	0.014545	3.71836	Up	cell cycle associated protein 1
ILMN_1730794	SERTAD4	0.292851	9.6E-05	0.008175	4.051978	Up	SERTA domain containing 4
ILMN_2394250	PLEKHA1	0.291747	0.002354	0.03774	3.115333	Up	pleckstrin homology domain containing A1
ILMN_2078389	SLC4A2	0.291257	0.001113	0.025759	3.35042	Up	solute carrier family 4 member 2
ILMN_2220403	GINM1	0.290762	0.000493	0.01788	3.593513	Up	glycoprotein integral membrane 1
ILMN_1710027	PNMT	0.290062	0.0003	0.014133	3.736366	Up	phenylethanolamine N-methyltransferase
ILMN_1734478	PIP5K1B	0.289858	5.33E-06	0.002879	4.790836	Up	phosphatidylinositol-4-phosphate 5-kinase type 1 β
ILMN_1758034	ETFDH	0.288994	0.000644	0.019798	3.514851	Up	electron transfer flavoprotein dehydrogenase
ILMN_1666713	LYPLA1	0.287789	0.00059	0.019114	3.541151	Up	lysophospholipase 1
ILMN_1797964	ARL6IP6	0.287061	0.000378	0.015488	3.670358	Up	ADP ribosylation factor like GTPase 6 interacting protein 6
ILMN_3290211	PIGH	0.286739	0.00285	0.041916	3.053413	Up	phosphatidylinositol glycan anchor biosynthesis class H
ILMN_1687546	HSP90AA1	0.286692	0.000957	0.02406	3.396413	Up	heat shock protein 90 α family class A member 1
ILMN_1719344	NRBF2	0.286671	0.002233	0.036626	3.132293	Up	nuclear receptor binding factor 2
ILMN_1734655	ATG9B	0.286517	0.000583	0.01904	3.544278	Up	autophagy related 9B
ILMN_1711408	ANXA4	0.286403	0.002191	0.036239	3.138437	Up	annexin A4
ILMN_1811178	SCAPER	0.286258	0.002028	0.035067	3.163085	Up	S-phase cyclin A associated protein in the ER
ILMN_1669281	CLN3	0.285791	0.000543	0.018404	3.565093	Up	CLN3 lysosomal/endosomaltransmembrane protein, battenin
ILMN_1686985	MTM1	0.284755	0.00147	0.029797	3.264326	Up	myotubularin 1
ILMN_1781560	ST3GAL6	0.284681	0.000826	0.02247	3.440705	Up	ST3 β-galactoside α-2,3-sialyltransferase 6
ILMN_1734229	SPPL2A	0.283933	0.001105	0.025674	3.352613	Up	signal peptide peptidase like 2A
ILMN_2094166	CHMP5	0.282317	0.002279	0.037103	3.125771	Up	charged multivesicular body protein 5
ILMN_1773849	ATP6V0C	0.282222	0.000886	0.023276	3.419682	Up	ATPase H+ transporting V0 subunit c
ILMN_1739876	RAB3GAP1	0.281181	0.001209	0.026753	3.324924	Up	RAB3 GTPase activating protein catalytic subunit 1
ILMN_1797594	NFAT5	0.28094	0.002689	0.040538	3.072332	Up	nuclear factor of activated T cells 5
ILMN_1734542	OVGP1	0.280116	0.00204	0.035106	3.161242	Up	oviductal glycoprotein 1
ILMN_1665982	AKTIP	0.277649	0.001862	0.03357	3.190258	Up	AKT interacting protein
ILMN_1679268	PELI1	0.277477	0.001777	0.032803	3.204955	Up	pellino E3 ubiquitin protein ligase 1
ILMN_3249846	LIMS3-LOC440895	0.276865	0.001178	0.026374	3.333032	Up	LIMS3-LOC440895 readthrough
ILMN_2323526	WAC	0.276545	0.000268	0.013383	3.769114	Up	WW domain containing adaptor with coiled-coil
ILMN_1748077	DDX59	0.275827	0.00214	0.035854	3.145909	Up	DEAD-box helicase 59
ILMN_1782444	YIPF4	0.275515	0.001307	0.027959	3.300882	Up	Yip1 domain family member 4
ILMN_2339284	CHD2	0.27514	0.000192	0.011354	3.862717	Up	chromodomain helicase DNA binding protein 2
ILMN_1706342	ZNF746	0.274797	0.001538	0.03037	3.250211	Up	zinc finger protein 746
ILMN_3215367	PPP4R2	0.274625	0.000744	0.021279	3.472213	Up	protein phosphatase 4 regulatory subunit 2
ILMN_1687279	DHPS	0.274317	0.000812	0.022284	3.445971	Up	deoxyhypusine synthase
ILMN_1685678	EEF1B2	0.273865	0.001055	0.025169	3.366614	Up	eukaryotic translation elongation factor 1 β 2
ILMN_1690066	TIGD2	0.273626	0.000317	0.014515	3.720901	Up	tigger transposable element derived 2
ILMN_1736752	COMTD1	0.273513	0.001712	0.032189	3.216644	Up	catechol-O-methyltransferase domain containing 1
ILMN_2387090	CGGBP1	0.273351	0.000341	0.01488	3.699974	Up	CGG triplet repeat binding protein 1
ILMN_2194627	GMCL1	0.273232	0.000277	0.013644	3.759819	Up	germ cell-less 1, spermatogenesis associated
ILMN_3241234	S100A11	0.273188	0.000851	0.022765	3.431703	Up	S100 calcium binding protein A11
ILMN_1678454	CASP4	0.27189	0.00153	0.030267	3.25192	Up	caspase 4
ILMN_1705907	NUP153	0.271384	0.000255	0.013135	3.782266	Up	nucleoporin 153
ILMN_2106265	GDPD1	0.271243	0.003428	0.046055	2.992682	Up	glycerophosphodiesterphosphodiesterase domain containing 1
ILMN_1699357	SLC22A5	0.270646	0.003156	0.044202	3.019938	Up	solute carrier family 22 member 5
ILMN_3282768	PPP1R14B	0.270021	0.001182	0.026432	3.331779	Up	protein phosphatase 1 regulatory inhibitor subunit 14B
ILMN_1784655	TLCD1	0.269664	0.001698	0.03211	3.219351	Up	TLC domain containing 1
ILMN_1809344	BTBD10	0.269367	0.003946	0.049535	2.945897	Up	BTB domain containing 10
ILMN_1651268	BORCS5	0.268841	0.000523	0.0181	3.576152	Up	BLOC-1 related complex subunit 5
ILMN_1676385	PAK2	0.268282	0.000156	0.010361	3.920545	Up	p21 (RAC1) activated kinase 2
ILMN_1658337	AKIRIN1	0.268214	0.003147	0.04417	3.020957	Up	akirin 1
ILMN_2137464	DVL3	0.267864	0.0012	0.026647	3.327306	Up	dishevelled segment polarity protein 3
ILMN_1721833	IER5	0.26766	0.003092	0.043799	3.026706	Up	immediate early response 5
ILMN_1781431	GLCCI1	0.267281	8.05E-05	0.007462	4.099378	Up	glucocorticoid induced 1
ILMN_1808824	NEBL	0.266945	0.001597	0.031164	3.238616	Up	nebulette
ILMN_1813028	CBX5	0.266695	4.39E-05	0.005755	4.259566	Up	chromobox 5
ILMN_1717745	TIAL1	0.266333	6.37E-06	0.00295	4.747368	Up	TIA1 cytotoxic granule associated RNA binding protein like 1
ILMN_1695110	BCAT2	0.266237	0.002867	0.042043	3.051398	Up	branched chain amino acid transaminase 2
ILMN_1735052	ULK1	0.266063	0.002947	0.042626	3.042442	Up	unc-51 like autophagy activating kinase 1
ILMN_1666670	RBX1	0.265833	9.11E-06	0.003026	4.659225	Up	ring-box 1
ILMN_1801476	CDS1	0.265788	0.002179	0.036176	3.140131	Up	CDP-diacylglycerol synthase 1
ILMN_1707350	TUSC1	0.265484	0.002186	0.036205	3.139179	Up	tumor suppressor candidate 1
ILMN_1671265	ING2	0.264936	0.000146	0.010103	3.937352	Up	inhibitor of growth family member 2
ILMN_1776297	GOLGA4	0.262744	0.001009	0.024621	3.380238	Up	golgin A4
ILMN_1717063	FBXO9	0.262663	5.06E-06	0.002879	4.803593	Up	F-box protein 9
ILMN_1791826	RAB25	0.262537	0.003142	0.04417	3.021403	Up	RAB25, member RAS oncogene family
ILMN_1704550	AZIN1	0.262282	0.002375	0.03792	3.112496	Up	antizyme inhibitor 1
ILMN_1660111	UCHL3	0.262244	4.55E-05	0.005773	4.250481	Up	ubiquitin C-terminal hydrolase L3
ILMN_1709043	PLGRKT	0.26194	0.001065	0.025246	3.363896	Up	plasminogen receptor with a C-terminal lysine
ILMN_1695961	CLK3	0.261157	6.84E-05	0.006944	4.142743	Up	CDC like kinase 3
ILMN_3197097	TSTD1	0.260702	0.000162	0.010401	3.909022	Up	thiosulfate sulfurtransferase like domain containing 1
ILMN_1792497	AGFG1	0.259601	0.000823	0.022397	3.442009	Up	ArfGAP with FG repeats 1
ILMN_1684346	TNFAIP8L1	0.259593	0.001641	0.031522	3.229929	Up	TNF α induced protein 8 like 1
ILMN_1737475	ABHD11	0.259331	0.002117	0.035667	3.149412	Up	abhydrolase domain containing 11
ILMN_1682147	HOOK2	0.258381	0.000116	0.009	4.000611	Up	hook microtubule tethering protein 2
ILMN_1736154	LZTS3	0.257357	0.000288	0.013983	3.747905	Up	leucine zipper tumor suppressor family member 3
ILMN_2328776	STK26	0.257332	0.003144	0.04417	3.021189	Up	serine/threonine kinase 26
ILMN_3246900	LINC01278	0.255848	0.002475	0.03872	3.09914	Up	long intergenic non-protein coding RNA 1278
ILMN_1702407	SPIN1	0.255702	0.001221	0.026874	3.322	Up	spindlin 1
ILMN_2344956	ACP1	0.255631	0.001788	0.032895	3.202956	Up	acid phosphatase 1
ILMN_1685415	HBP1	0.25552	0.00362	0.04748	2.974605	Up	HMG-box transcription factor 1
ILMN_2399264	SEPTIN6	0.25419	0.000112	0.008891	4.010048	Up	septin 6
ILMN_2055523	CSGALNACT1	0.253386	0.002808	0.041529	3.058213	Up	chondroitin sulfate N-acetylgalactosaminyltransferase 1
ILMN_3279712	SMS	0.252553	0.001715	0.032189	3.216153	Up	spermine synthase
ILMN_1701514	TRAF3IP2	0.252377	0.003172	0.044294	3.018317	Up	TRAF3 interacting protein 2
ILMN_3227529	RPS13	0.252336	0.000401	0.01601	3.65339	Up	ribosomal protein S13
ILMN_1680397	CXCR2	0.251961	0.000716	0.020858	3.483454	Up	C-X-C motif chemokine receptor 2
ILMN_1661142	TMF1	0.251932	0.000831	0.02251	3.439126	Up	TATA element modulatory factor 1
ILMN_2228044	TBC1D23	0.251655	5.18E-06	0.002879	4.797583	Up	TBC1 domain family member 23
ILMN_2352326	COASY	0.25148	0.003205	0.044541	3.014949	Up	Coenzyme A synthase
ILMN_1753457	PKP3	0.251362	0.000802	0.02216	3.449557	Up	plakophilin 3
ILMN_2081673	INSL6	0.250409	6.77E-06	0.00295	4.732468	Up	insulin like 6
ILMN_1743396	ACOX3	0.250377	0.001149	0.026102	3.340671	Up	acyl-CoA oxidase 3, pristanoyl
ILMN_1711786	NFE2	0.250104	0.001205	0.026691	3.326009	Up	nuclear factor, erythroid 2
ILMN_3289090	CAPZA1	0.24988	0.001472	0.029809	3.263922	Up	capping actin protein of muscle Z-line subunit α 1
ILMN_2151056	BORCS7	0.249315	0.000261	0.013186	3.776529	Up	BLOC-1 related complex subunit 7
ILMN_1716195	H2BC8	0.248687	0.00072	0.020871	3.481999	Up	H2B clustered histone 8
ILMN_2366864	JUP	0.248584	0.003341	0.045507	3.001191	Up	junction plakoglobin
ILMN_1709026	PXDC1	0.248166	0.00183	0.033266	3.19572	Up	PX domain containing 1
ILMN_1690894	HSP90B3P	0.248104	0.001371	0.028675	3.2861	Up	heat shock protein 90 β family member 3, pseudogene
ILMN_1662880	LINC01554	0.248026	0.000163	0.010418	3.908142	Up	long intergenic non-protein coding RNA 1554
ILMN_1664560	DYRK1A	0.248006	0.003816	0.048801	2.957108	Up	dual specificity tyrosine phosphorylation regulated kinase 1A
ILMN_3241665	SERTAD4-AS1	0.247969	2.49E-05	0.004816	4.406676	Up	SERTAD4 antisense RNA 1
ILMN_2347541	NIN	0.24778	0.002743	0.041015	3.065875	Up	ninein
ILMN_3185198	ACTR3C	0.247744	0.000969	0.024168	3.392602	Up	actin related protein 3C
ILMN_2274420	SPTLC1	0.247643	0.001511	0.030094	3.255789	Up	serine palmitoyltransferase long chain base subunit 1
ILMN_2356654	LGALS8	0.247618	0.000254	0.013135	3.783998	Up	galectin 8
ILMN_1711792	GPBP1	0.246944	0.000145	0.010064	3.939077	Up	GC-rich promoter binding protein 1
ILMN_1785765	TM9SF2	0.246663	0.003558	0.047012	2.980358	Up	transmembrane 9 superfamily member 2
ILMN_1739967	TBK1	0.246267	0.000318	0.01452	3.72025	Up	TANK binding kinase 1
ILMN_1737005	SMG9	0.245801	9.57E-06	0.003088	4.647172	Up	SMG9 nonsense mediated mRNA decay factor
ILMN_3243514	PP12613	0.245456	5.23E-05	0.006073	4.213967	Up	uncharacterized LOC100192379
ILMN_2113938	TOR1AIP2	0.245454	0.001286	0.027708	3.306016	Up	torsin 1A interacting protein 2
ILMN_1669905	DCP2	0.24544	0.001721	0.032249	3.215129	Up	decapping mRNA 2
ILMN_1667977	TAF1B	0.245081	0.000116	0.009	4.001937	Up	TATA-box binding protein associated factor, RNA polymerase I subunit B
ILMN_1765212	LARP1B	0.244844	0.001587	0.031031	3.240414	Up	La ribonucleoprotein 1B
ILMN_2275248	ECE2	0.244549	0.000156	0.010362	3.919402	Up	endothelin converting enzyme 2
ILMN_1697864	CXorf38	0.243864	7.18E-05	0.007104	4.130027	Up	chromosome X open reading frame 38
ILMN_1771286	PDE4DIP	0.242808	0.001603	0.031243	3.237261	Up	phosphodiesterase 4D interacting protein
ILMN_1804064	ESRRG	0.242656	2.74E-05	0.004869	4.381513	Up	estrogen related receptor γ
ILMN_1808860	STX5	0.241476	0.001945	0.034306	3.176409	Up	syntaxin 5
ILMN_2359345	NET1	0.241323	0.001147	0.026081	3.341062	Up	neuroepithelial cell transforming 1
ILMN_2324157	UBA3	0.24102	0.000534	0.018273	3.570218	Up	ubiquitin like modifier activating enzyme 3
ILMN_1778803	ZFAND6	0.239907	0.002388	0.037999	3.110718	Up	zinc finger AN1-type containing 6
ILMN_1810782	SH3KBP1	0.239001	0.003315	0.045247	3.003739	Up	SH3 domain containing kinase binding protein 1
ILMN_1666258	AMFR	0.238013	0.002066	0.035418	3.157186	Up	autocrine motility factor receptor
ILMN_1776154	COG3	0.237721	0.001616	0.031301	3.234779	Up	component of oligomericgolgi complex 3
ILMN_2387553	PSMA3	0.237489	0.000128	0.009505	3.973908	Up	proteasome 20S subunit α 3
ILMN_1730630	CXorf56	0.236626	0.002038	0.035103	3.1615	Up	chromosome X open reading frame 56
ILMN_1673380	GNG12	0.236565	0.003033	0.04332	3.033043	Up	G protein subunit γ 12
ILMN_3247111	LRRC69	0.233635	0.000701	0.020587	3.489939	Up	leucine rich repeat containing 69
ILMN_1757956	PCGF1	0.23308	0.000729	0.021012	3.478289	Up	polycomb group ring finger 1
ILMN_1759460	TAF7	0.232964	1.99E-06	0.002078	5.027658	Up	TATA-box binding protein associated factor 7
ILMN_1747241	IWS1	0.232787	0.00339	0.045729	2.996419	Up	interacts with SUPT6H, CTD assembly factor 1
ILMN_1676763	PIPSL	0.232594	0.000323	0.014545	3.715885	Up	PIP5K1A and PSMD4 like (pseudogene)
ILMN_1813148	TOM1	0.23244	0.002966	0.04284	3.040312	Up	target of myb1 membrane trafficking protein
ILMN_3240721	TDG	0.231924	0.000256	0.013135	3.782108	Up	thymine DNA glycosylase
ILMN_1682919	PAFAH2	0.231731	0.002527	0.039088	3.09253	Up	platelet activating factor acetylhydrolase 2
ILMN_2306077	USP33	0.231641	1.33E-05	0.00347	4.564689	Up	ubiquitin specific peptidase 33
ILMN_2413572	MARK2	0.231373	0.003901	0.049215	2.94973	Up	microtubule affinity regulating kinase 2
ILMN_2320853	UBE2D3	0.230668	0.001111	0.025736	3.351016	Up	ubiquitin conjugating enzyme E2 D3
ILMN_1760256	RBM22	0.230632	0.001806	0.033086	3.199833	Up	RNA binding motif protein 22
ILMN_1658743	CCNDBP1	0.229972	9.97E-05	0.008353	4.04187	Up	cyclin D1 binding protein 1
ILMN_1717294	PTPN3	0.229265	0.002347	0.037683	3.116328	Up	protein tyrosine phosphatase non-receptor type 3
ILMN_2101920	HNRNPH1	0.229246	0.000147	0.010107	3.93673	Up	heterogeneous nuclear ribonucleoprotein H1
ILMN_1736234	CHTOP	0.228517	0.001144	0.026058	3.341855	Up	chromatin target of PRMT1
ILMN_1700384	KIAA1522	0.228516	0.000577	0.018974	3.547666	Up	KIAA1522
ILMN_1719237	SPDYE8P	0.228465	0.00384	0.048887	2.954952	Up	speedy/RINGO cell cycle regulator family member E8, pseudogene
ILMN_1701724	GET4	0.228197	0.000247	0.012906	3.79222	Up	guided entry of tail-anchored proteins factor 4
ILMN_1785852	NABP1	0.227819	0.002071	0.035437	3.156321	Up	nucleic acid binding protein 1
ILMN_1755649	SLC16A5	0.227432	0.001462	0.029722	3.266055	Up	solute carrier family 16 member 5
ILMN_1742118	RNASE12	0.226586	0.000101	0.008365	4.039673	Up	ribonuclease A family member 12 (inactive)
ILMN_1701169	HP1BP3	0.226445	0.000651	0.019899	3.512006	Up	heterochromatin protein 1 binding protein 3
ILMN_1754179	AP1G2	0.226367	0.000599	0.019237	3.536286	Up	adaptor related protein complex 1 subunit γ 2
ILMN_2396813	BABAM1	0.226351	0.000283	0.013823	3.753519	Up	BRISC and BRCA1 A complex member 1
ILMN_1726589	CD248	−0.87731	6.86E-05	0.006945	−4.14215	Down	CD248 molecule
ILMN_1658356	PAMR1	−0.77303	1.88E-07	0.000867	−5.57122	Down	peptidase domain containing associated with muscle regeneration 1
ILMN_1701308	COL1A1	−0.77213	5.05E-07	0.001127	−5.34677	Down	collagen type I α 1 chain
ILMN_1723522	APOLD1	−0.75887	0.00051	0.017953	−3.58362	Down	apolipoprotein L domain containing 1
ILMN_1779875	THY1	−0.75181	3.89E-05	0.005414	−4.29148	Down	Thy-1 cell surface antigen
ILMN_1696347	CTSC	−0.73439	0.000361	0.015151	−3.68404	Down	cathepsin C
ILMN_1706505	COL5A1	−0.68289	8.23E-08	0.000792	−5.75462	Down	collagen type V α 1 chain
ILMN_3237946	PXDN	−0.68073	4.96E-06	0.002879	−4.80838	Down	peroxidasin
ILMN_1673639	ABI3BP	−0.67199	0.000934	0.023743	−3.40373	Down	ABI family member 3 binding protein
ILMN_1766914	MFAP4	−0.66612	4.35E-06	0.002772	−4.84032	Down	microfibril associated protein 4
ILMN_1795325	ACTG2	−0.65224	0.000984	0.024307	−3.38785	Down	actin γ 2, smooth muscle
ILMN_1757604	TPM2	−0.65	0.00014	0.00984	−3.94965	Down	tropomyosin 2
ILMN_1706643	COL6A3	−0.64553	2.54E-05	0.004833	−4.40149	Down	collagen type VI α 3 chain
ILMN_1725193	IGFBP2	−0.63091	0.001035	0.024881	−3.3724	Down	insulin like growth factor binding protein 2
ILMN_1720231	TNNT3	−0.61868	0.000693	0.020442	−3.49345	Down	troponin T3, fast skeletal type
ILMN_2104356	COL1A2	−0.60473	8.52E-06	0.00295	−4.67573	Down	collagen type I α 2 chain
ILMN_1773079	COL3A1	−0.6033	5.14E-06	0.002879	−4.79969	Down	collagen type III α 1 chain
ILMN_1707070	PCOLCE	−0.59908	1.55E-06	0.001955	−5.0867	Down	procollagen C-endopeptidase enhancer
ILMN_1797776	PRSS23	−0.59791	1.36E-06	0.001955	−5.11653	Down	serine protease 23
ILMN_2390919	FBLN2	−0.59331	6.9E-06	0.00295	−4.72775	Down	fibulin 2
ILMN_1712046	CPXM1	−0.59179	0.000142	0.009889	−3.94586	Down	carboxypeptidase X, M14 family member 1
ILMN_1670379	ANTXR1	−0.59159	3.06E-05	0.005173	−4.35299	Down	ANTXR cell adhesion molecule 1
ILMN_1743445	FAM107A	−0.58708	0.001007	0.02461	−3.38091	Down	family with sequence similarity 107 member A
ILMN_1697268	EMILIN2	−0.58178	2.61E-05	0.004833	−4.39456	Down	elastin microfibrilinterfacer 2
ILMN_1756071	MFGE8	−0.58136	0.00023	0.012505	−3.81152	Down	milk fat globule-EGF factor 8 protein
ILMN_2115125	CCN2	−0.56274	0.00135	0.028444	−3.29078	Down	cellular communication network factor 2
ILMN_1700690	VAT1	−0.55561	1.38E-05	0.003478	−4.55564	Down	vesicle amine transport 1
ILMN_1761968	PPP1R14A	−0.55382	6.36E-06	0.00295	−4.74771	Down	protein phosphatase 1 regulatory inhibitor subunit 14A
ILMN_1783909	COL6A2	−0.55352	3.46E-05	0.005189	−4.32174	Down	collagen type VI α 2 chain
ILMN_2384056	GPER1	−0.55173	0.001275	0.027557	−3.30852	Down	G protein-coupled estrogen receptor 1
ILMN_1688642	LAMC3	−0.54794	0.000167	0.010606	−3.90071	Down	laminin subunit γ 3
ILMN_1779558	GAS6	−0.54545	4.6E-07	0.001127	−5.36823	Down	growth arrest specific 6
ILMN_1800787	RFTN1	−0.54246	9.71E-06	0.003091	−4.6434	Down	raftlin, lipid raft linker 1
ILMN_1665909	LASP1	−0.53453	1.44E-06	0.001955	−5.10383	Down	LIM and SH3 protein 1
ILMN_1811313	SLIT3	−0.53304	5.6E-08	0.000792	−5.83956	Down	slit guidance ligand 3
ILMN_1793476	CAVIN3	−0.5317	2.2E-05	0.004675	−4.43772	Down	caveolae associated protein 3
ILMN_2307903	VCAM1	−0.53139	8.12E-05	0.007511	−4.09693	Down	vascular cell adhesion molecule 1
ILMN_1656560	PARM1	−0.53103	0.00016	0.010366	−3.91255	Down	prostate androgen-regulated mucin-like protein 1
ILMN_1672611	CDH11	−0.52302	0.000212	0.011964	−3.83473	Down	cadherin 11
ILMN_1765557	OLFML2B	−0.52097	4.2E-06	0.00273	−4.84868	Down	olfactomedin like 2B
ILMN_1815057	PDGFRB	−0.52016	1.82E-05	0.004138	−4.48559	Down	platelet derived growth factor receptor β
ILMN_1736178	AEBP1	−0.5199	3.33E-05	0.005189	−4.33169	Down	AE binding protein 1
ILMN_1748124	TSC22D3	−0.51824	0.000928	0.02368	−3.40564	Down	TSC22 domain family member 3
ILMN_1723978	LGALS1	−0.51718	0.000364	0.015227	−3.68144	Down	galectin 1
ILMN_1738147	NES	−0.51576	8.77E-06	0.003014	−4.6686	Down	nestin
ILMN_2301722	PDE8B	−0.51488	2.73E-05	0.004869	−4.38289	Down	phosphodiesterase 8B
ILMN_1687301	VCAN	−0.51483	0.000221	0.012223	−3.82288	Down	versican
ILMN_1778523	KLF9	−0.50597	5.25E-05	0.006073	−4.21296	Down	Kruppel like factor 9
ILMN_2062468	IGFBP7	−0.50184	0.000947	0.023893	−3.3994	Down	insulin like growth factor binding protein 7
ILMN_1748323	CXCL14	−0.49871	0.000454	0.017215	−3.61778	Down	C-X-C motif chemokine ligand 14
ILMN_1751326	FAM162B	−0.49815	0.00278	0.041278	−3.06149	Down	family with sequence similarity 162 member B
ILMN_2373791	ENPP2	−0.49776	6.94E-05	0.006958	−4.13908	Down	ectonucleotidepyrophosphatase/phosphodiesterase 2
ILMN_1752968	LAMB2	−0.49708	0.000303	0.014156	−3.73363	Down	laminin subunit β 2
ILMN_1654324	HEYL	−0.49611	0.000102	0.008419	−4.03652	Down	hes related family bHLH transcription factor with YRPW motif like
ILMN_1667295	VASN	−0.49482	1.16E-05	0.003364	−4.5987	Down	vasorin
ILMN_1812618	ARAP3	−0.49404	0.003139	0.044148	−3.0217	Down	ArfGAP with RhoGAP domain, ankyrin repeat and PH domain 3
ILMN_1661599	DDIT4	−0.49301	0.002513	0.039003	−3.09422	Down	DNA damage inducible transcript 4
ILMN_1713496	ST3GAL5	−0.492	8.48E-05	0.007599	−4.08534	Down	ST3 β-galactoside α-2,3-sialyltransferase 5
ILMN_1665865	IGFBP4	−0.49057	0.002826	0.041676	−3.05613	Down	insulin like growth factor binding protein 4
ILMN_1687652	TGFB3	−0.48958	0.002071	0.035437	−3.15638	Down	transforming growth factor β 3
ILMN_1801616	EMP1	−0.48942	0.000266	0.013333	−3.77097	Down	epithelial membrane protein 1
ILMN_1733259	TMIGD3	−0.48909	0.00024	0.012746	−3.79958	Down	transmembrane and immunoglobulin domain containing 3
ILMN_1670490	PDPN	−0.48881	0.000138	0.009821	−3.95297	Down	podoplanin
ILMN_1665219	LTBP4	−0.4882	1.05E-07	0.000838	−5.70174	Down	latent transforming growth factor β binding protein 4
ILMN_1738578	FILIP1L	−0.48742	0.000178	0.010961	−3.88315	Down	filamin A interacting protein 1 like
ILMN_1654109	EGFLAM	−0.48718	0.000239	0.012746	−3.80108	Down	EGF like, fibronectin type III and laminin G domains
ILMN_1796734	SPARC	−0.48641	1.76E-05	0.004057	−4.49427	Down	secreted protein acidic and cysteine rich
ILMN_1675797	EPDR1	−0.48638	0.002889	0.0423	−3.04891	Down	ependymin related 1
ILMN_1752755	VWF	−0.48403	0.000698	0.02056	−3.491	Down	von Willebrand factor
ILMN_1743836	MXRA7	−0.48256	0.000135	0.009758	−3.9585	Down	matrix remodeling associated 7
ILMN_1732151	COL6A1	−0.48252	0.000537	0.018298	−3.56848	Down	collagen type VI α 1 chain
ILMN_1699695	TNFRSF21	−0.48252	3.82E-07	0.001058	−5.41081	Down	TNF receptor superfamily member 21
ILMN_1671703	ACTA2	−0.48127	0.001006	0.024602	−3.3812	Down	actin α 2, smooth muscle
ILMN_1777190	CFD	−0.48004	0.001794	0.032947	−3.20188	Down	complement factor D
ILMN_1785646	PMP22	−0.47862	1.24E-05	0.003412	−4.58237	Down	peripheral myelin protein 22
ILMN_1795166	PTH1R	−0.47706	1.22E-05	0.003404	−4.58718	Down	parathyroid hormone 1 receptor
ILMN_1779182	TMEM98	−0.47485	3.81E-06	0.002678	−4.87203	Down	transmembrane protein 98
ILMN_3248591	LTBP2	−0.47272	3.28E-05	0.005189	−4.33519	Down	latent transforming growth factor β binding protein 2
ILMN_1672503	DPYSL2	−0.47143	5.37E-06	0.002879	−4.78877	Down	dihydropyrimidinase like 2
ILMN_2223941	FBLN5	−0.47095	1.94E-06	0.002072	−5.03358	Down	fibulin 5
ILMN_1688480	CCND1	−0.46977	0.000483	0.017744	−3.59964	Down	cyclin D1
ILMN_1808114	LYVE1	−0.46936	0.003925	0.049436	−2.9477	Down	lymphatic vessel endothelial hyaluronan receptor 1
ILMN_2087692	CYBRD1	−0.46894	2.69E-05	0.004861	−4.38626	Down	cytochrome b reductase 1
ILMN_1808707	FSCN1	−0.46767	3.69E-05	0.005267	−4.30499	Down	fascin actin-bundling protein 1
ILMN_1660808	WFDC1	−0.46665	0.000163	0.010418	−3.90783	Down	WAP four-disulfide core domain 1
ILMN_2337655	WARS1	−0.4653	4.87E-05	0.005873	−4.23248	Down	tryptophanyl-tRNAsynthetase 1
ILMN_2347145	DCN	−0.46414	8.34E-05	0.007533	−4.08985	Down	decorin
ILMN_1694840	MATN2	−0.46405	0.000542	0.018404	−3.56578	Down	matrilin 2
ILMN_1729117	COL5A2	−0.46318	0.000177	0.010947	−3.88468	Down	collagen type V α 2 chain
ILMN_1681983	RSPO3	−0.46314	0.000248	0.012955	−3.79059	Down	R-spondin 3
ILMN_3246401	AIF1L	−0.46241	0.000187	0.011245	−3.86955	Down	allograft inflammatory factor 1 like
ILMN_1778668	TAGLN	−0.46167	0.001483	0.029912	−3.26159	Down	transgelin
ILMN_1700183	APLNR	−0.46027	0.000159	0.010362	−3.91414	Down	apelin receptor
ILMN_2413158	PODXL	−0.45912	0.000113	0.008916	−4.0078	Down	podocalyxin like
ILMN_1701877	AXL	−0.45803	3.57E-05	0.005231	−4.31359	Down	AXL receptor tyrosine kinase
ILMN_1676893	ADCY3	−0.45717	5.23E-05	0.006073	−4.21395	Down	adenylatecyclase 3
ILMN_1660086	MYH11	−0.45693	0.00135	0.028444	−3.29091	Down	myosin heavy chain 11
ILMN_1781149	INMT	−0.45264	0.000222	0.012233	−3.82134	Down	indolethylamine N-methyltransferase
ILMN_1671928	PROS1	−0.45214	5.66E-05	0.006347	−4.19283	Down	protein S
ILMN_2377900	MAP1B	−0.45172	0.000292	0.014028	−3.74419	Down	microtubule associated protein 1B
ILMN_1691127	VTN	−0.45044	5.91E-06	0.002933	−4.76576	Down	vitronectin
ILMN_1701441	LPAR1	−0.45043	2.49E-05	0.004816	−4.40602	Down	lysophosphatidic acid receptor 1
ILMN_1734190	TCEAL3	−0.45031	0.000148	0.010152	−3.93406	Down	transcription elongation factor A like 3
ILMN_1696749	LMNA	−0.44963	7.46E-06	0.00295	−4.70841	Down	lamin A/C
ILMN_3236825	RAPGEF5	−0.4488	0.00152	0.030129	−3.25398	Down	Rap guanine nucleotide exchange factor 5
ILMN_1791890	SPON1	−0.44793	0.000369	0.01532	−3.67762	Down	spondin 1
ILMN_1656951	APCDD1	−0.44608	5.84E-05	0.006463	−4.18452	Down	APC down-regulated 1
ILMN_1723480	BST2	−0.44433	3.39E-05	0.005189	−4.32664	Down	bone marrow stromal cell antigen 2
ILMN_1651429	SELENOM	−0.44235	0.000729	0.021012	−3.47803	Down	selenoprotein M
ILMN_1758164	STC1	−0.44235	5.34E-05	0.006098	−4.20849	Down	stanniocalcin 1
ILMN_1728197	CLDN5	−0.44196	0.002297	0.037268	−3.1232	Down	claudin 5
ILMN_1663866	TGFBI	−0.44055	0.000185	0.011135	−3.87297	Down	transforming growth factor β induced
ILMN_2057479	EGFL6	−0.44039	0.002923	0.042546	−3.04508	Down	EGF like domain multiple 6
ILMN_1784863	CD36	−0.43914	0.001443	0.029457	−3.27014	Down	CD36 molecule
ILMN_1789492	ZDHHC8	−0.43853	1.97E-05	0.004301	−4.4659	Down	zinc finger DHHC-type containing 8
ILMN_1790689	CRISPLD2	−0.43782	0.000578	0.018974	−3.54695	Down	cysteine rich secretory protein LCCL domain containing 2
ILMN_1795442	LAMA4	−0.43771	8.57E-05	0.007637	−4.08249	Down	laminin subunit α 4
ILMN_1702501	RPS6KA2	−0.43678	2.81E-06	0.002369	−4.94542	Down	ribosomal protein S6 kinase A2
ILMN_1671106	GJA4	−0.43644	0.000331	0.014708	−3.70883	Down	gap junction protein α 4
ILMN_1692058	NDN	−0.43561	2.4E-05	0.004755	−4.41545	Down	necdin, MAGE family member
ILMN_1668629	C4orf48	−0.43536	3.2E-05	0.005189	−4.34205	Down	chromosome 4 open reading frame 48
ILMN_1697448	TXNIP	−0.43462	4.43E-05	0.005755	−4.25718	Down	thioredoxin interacting protein
ILMN_1798360	ACKR3	−0.43355	0.000768	0.021647	−3.46258	Down	atypical chemokine receptor 3
ILMN_1671565	RNASET2	−0.43033	0.000961	0.024096	−3.39507	Down	ribonuclease T2
ILMN_1676449	SLIT2	−0.42992	0.000142	0.009889	−3.94603	Down	slit guidance ligand 2
ILMN_1687978	PHLDA1	−0.42734	1.82E-05	0.004138	−4.48565	Down	pleckstrin homology like domain family A member 1
ILMN_1715991	CAVIN2	−0.42665	0.001128	0.025959	−3.34614	Down	caveolae associated protein 2
ILMN_2067656	CCND2	−0.42587	0.000216	0.012027	−3.82966	Down	cyclin D2
ILMN_1680037	RIPOR1	−0.42581	3.23E-06	0.002557	−4.91159	Down	RHO family interacting cell polarization regulator 1
ILMN_1669409	VSIG4	−0.42564	0.000387	0.015721	−3.66386	Down	V-set and immunoglobulin domain containing 4
ILMN_1673566	ADAMTS1	−0.42513	0.000988	0.024354	−3.3866	Down	ADAM metallopeptidase with thrombospondin type 1 motif 1
ILMN_1709486	SRPX	−0.42497	0.000723	0.020945	−3.48076	Down	sushi repeat containing protein X-linked
ILMN_2308849	MYADM	−0.42477	0.00028	0.013713	−3.75666	Down	myeloid associated differentiation marker
ILMN_1742534	COL4A5	−0.42458	3.49E-05	0.005194	−4.31961	Down	collagen type IV α 5 chain
ILMN_1662419	COX7A1	−0.42139	0.001898	0.033868	−3.1842	Down	cytochrome c oxidase subunit 7A1
ILMN_1666894	CSPG4	−0.42081	0.000309	0.014326	−3.72785	Down	chondroitin sulfate proteoglycan 4
ILMN_1681679	TSPO	−0.41878	0.000143	0.009968	−3.9429	Down	translocator protein
ILMN_2410929	PAPSS2	−0.41817	0.000199	0.011586	−3.85207	Down	3′-phosphoadenosine 5′-phosphosulfate synthase 2
ILMN_1675936	HIGD1B	−0.41805	0.002299	0.037268	−3.12291	Down	HIG1 hypoxia inducible domain family member 1B
ILMN_1800697	LDB2	−0.41769	0.000812	0.022284	−3.44608	Down	LIM domain binding 2
ILMN_1695959	EVA1C	−0.41726	1.48E-06	0.001955	−5.09704	Down	eva-1 homolog C
ILMN_1815700	WNT3A	−0.41588	0.000685	0.020354	−3.49693	Down	Wnt family member 3A
ILMN_1672878	ABR	−0.41554	1.42E-06	0.001955	−5.1066	Down	ABR activator of RhoGEF and GTPase
ILMN_1653203	EFEMP2	−0.41486	1.59E-05	0.003762	−4.52062	Down	EGF containing fibulin extracellular matrix protein 2
ILMN_1705442	CMTM3	−0.41405	3.33E-05	0.005189	−4.33163	Down	CKLF like MARVEL transmembrane domain containing 3
ILMN_1678353	FARP1	−0.41327	3.99E-06	0.002688	−4.86121	Down	FERM, ARH/RhoGEF and pleckstrin domain protein 1
ILMN_1745963	FOLR2	−0.4129	0.002926	0.042546	−3.04483	Down	folate receptor β
ILMN_2189027	LIPG	−0.41278	0.002567	0.039356	−3.08744	Down	lipase G, endothelial type
ILMN_1673352	IFITM2	−0.41277	1.45E-05	0.003584	−4.54291	Down	interferon induced transmembrane protein 2
ILMN_1814327	AGTR1	−0.4127	0.003348	0.045512	−3.00053	Down	angiotensin II receptor type 1
ILMN_1727532	OLFML3	−0.41123	0.000299	0.014133	−3.73746	Down	olfactomedin like 3
ILMN_1764964	IFNGR2	−0.41058	0.000123	0.009352	−3.98429	Down	interferon γ receptor 2
ILMN_1756573	NDUFA4L2	−0.40838	0.001643	0.031536	−3.22967	Down	NDUFA4 mitochondrial complex associated like 2
ILMN_1653466	HES4	−0.40777	0.000111	0.008868	−4.013	Down	hes family bHLH transcription factor 4
ILMN_2396875	IGFBP3	−0.40708	0.001143	0.026058	−3.34212	Down	insulin like growth factor binding protein 3
ILMN_2038775	TUBB2A	−0.40565	0.001946	0.03431	−3.17614	Down	tubulin β 2A class IIa
ILMN_1812031	PALM	−0.40551	1.37E-05	0.003478	−4.55698	Down	paralemmin
ILMN_1709307	GPSM1	−0.4053	3.66E-05	0.005267	−4.30712	Down	G protein signaling modulator 1
ILMN_3246214	B4GAT1	−0.40496	3.87E-07	0.001058	−5.40781	Down	β-1,4-glucuronyltransferase 1
ILMN_1802411	ITGA1	−0.4038	3.16E-05	0.005189	−4.34541	Down	integrin subunit α 1
ILMN_1714861	CD68	−0.40235	0.001299	0.027871	−3.30268	Down	CD68 molecule
ILMN_1666819	PHLDB1	−0.40159	2.35E-05	0.004738	−4.42152	Down	pleckstrin homology like domain family B member 1
ILMN_1723481	CHST3	−0.40156	5.39E-06	0.002879	−4.78828	Down	carbohydrate sulfotransferase 3
ILMN_1724994	COL4A2	−0.40135	0.000401	0.01601	−3.65332	Down	collagen type IV α 2 chain
ILMN_1768483	KCNK3	−0.40133	4.77E-05	0.005826	−4.23792	Down	potassium two pore domain channel subfamily K member 3
ILMN_1717934	SYT11	−0.40115	2.98E-05	0.005073	−4.35983	Down	synaptotagmin 11
ILMN_1812968	SOX18	−0.40033	0.000526	0.018141	−3.57485	Down	SRY-box transcription factor 18
ILMN_2173611	MT1E	−0.39988	0.000353	0.015039	−3.69035	Down	metallothionein 1E
ILMN_1668283	HYAL2	−0.39864	0.000646	0.019821	−3.51392	Down	hyaluronidase 2
ILMN_1757440	DIPK1B	−0.39796	0.00033	0.014704	−3.70918	Down	divergent protein kinase domain 1B
ILMN_1773059	ADGRA2	−0.39739	0.000911	0.023503	−3.41136	Down	adhesion G protein-coupled receptor A2
ILMN_1795429	VCL	−0.39693	0.000348	0.014991	−3.69401	Down	vinculin
ILMN_1789733	CLIP3	−0.39665	1.53E-05	0.00373	−4.53031	Down	CAP-Gly domain containing linker protein 3
ILMN_1675062	MYL9	−0.39519	0.001559	0.030619	−3.24612	Down	myosin light chain 9
ILMN_1711566	TIMP1	−0.39465	0.001568	0.030761	−3.24426	Down	TIMP metallopeptidase inhibitor 1
ILMN_1682781	TEAD2	−0.39462	2.27E-05	0.004675	−4.42977	Down	TEA domain transcription factor 2
ILMN_1806733	COL18A1	−0.39225	0.000595	0.019227	−3.53859	Down	collagen type XVIII α 1 chain
ILMN_1769091	PRCP	−0.39168	0.000281	0.013751	−3.75557	Down	prolylcarboxypeptidase
ILMN_1691376	JAG1	−0.39137	0.000108	0.008727	−4.02057	Down	jagged canonical Notch ligand 1
ILMN_1808238	RBPMS2	−0.39047	2.27E-05	0.004675	−4.43015	Down	RNA binding protein, mRNA processing factor 2
ILMN_1684391	PLOD1	−0.38987	5.67E-06	0.002906	−4.77593	Down	procollagen-lysine,2-oxoglutarate 5-dioxygenase 1
ILMN_1755657	RASIP1	−0.38985	0.00111	0.025736	−3.35121	Down	Ras interacting protein 1
ILMN_1754795	FAT1	−0.38928	0.000803	0.02216	−3.44913	Down	FAT atypical cadherin 1
ILMN_1723684	ACKR1	−0.38904	0.000258	0.013173	−3.77982	Down	atypical chemokine receptor 1 (Duffy blood group)
ILMN_1763640	NCKAP5L	−0.38828	4.47E-05	0.005755	−4.25481	Down	NCK associated protein 5 like
ILMN_2066151	TEK	−0.38809	0.001131	0.025976	−3.34543	Down	TEK receptor tyrosine kinase
ILMN_1730995	AFAP1L2	−0.38806	6.29E-05	0.006657	−4.16513	Down	actin filament associated protein 1 like 2
ILMN_1676846	ABCE1	−0.38772	0.002141	0.035854	−3.14571	Down	ATP binding cassette subfamily E member 1
ILMN_2306540	PDE9A	−0.38716	0.000255	0.013135	−3.78258	Down	phosphodiesterase 9A
ILMN_1756920	ADAM15	−0.38698	2.31E-05	0.004723	−4.4258	Down	ADAM metallopeptidase domain 15
ILMN_1667460	SULF2	−0.38575	0.000356	0.015084	−3.68742	Down	sulfatase 2
ILMN_1778681	EBF1	−0.38542	0.000387	0.015721	−3.66397	Down	EBF transcription factor 1
ILMN_1810852	LAMC1	−0.38297	0.000107	0.008689	−4.02291	Down	laminin subunit γ 1
ILMN_1723123	FGFR3	−0.38138	0.000151	0.010247	−3.92785	Down	fibroblast growth factor receptor 3
ILMN_1741632	RAB3IL1	−0.381	1.3E-05	0.003464	−4.57014	Down	RAB3A interacting protein like 1
ILMN_2230025	PDLIM3	−0.381	0.000642	0.019792	−3.51588	Down	PDZ and LIM domain 3
ILMN_1772612	ANGPTL2	−0.37902	2.69E-05	0.004861	−4.38663	Down	angiopoietin like 2
ILMN_1810844	RARRES2	−0.37881	0.000884	0.023267	−3.42025	Down	retinoic acid receptor responder 2
ILMN_1738816	FOXO1	−0.37837	0.000525	0.018141	−3.57509	Down	forkhead box O1
ILMN_1689953	CD81	−0.37702	1.74E-07	0.000867	−5.58795	Down	CD81 molecule
ILMN_1651950	TPST1	−0.37597	3.72E-05	0.005297	−4.30272	Down	tyrosylproteinsulfotransferase 1
ILMN_1692731	TTYH3	−0.37535	3.37E-05	0.005189	−4.32847	Down	tweety family member 3
ILMN_1658835	CAV2	−0.37448	0.001154	0.026156	−3.33932	Down	caveolin 2
ILMN_1680453	ITM2C	−0.37416	0.000588	0.019112	−3.54215	Down	integral membrane protein 2C
ILMN_1702835	SH3BGRL	−0.37249	0.000342	0.01488	−3.69952	Down	SH3 domain binding glutamate rich protein like
ILMN_1732923	SIPA1L2	−0.37207	0.000758	0.021475	−3.46637	Down	signal induced proliferation associated 1 like 2
ILMN_1797009	F3	−0.37136	0.00032	0.014545	−3.71818	Down	coagulation factor III, tissue factor
ILMN_1738263	PIGU	−0.37121	0.00019	0.011345	−3.86457	Down	phosphatidylinositol glycan anchor biosynthesis class U
ILMN_1739946	VKORC1	−0.3684	0.000138	0.009805	−3.95412	Down	vitamin K epoxide reductase complex subunit 1
ILMN_1803312	DIMT1	−0.36765	2.11E-05	0.004531	−4.44861	Down	DIMT1 rRNAmethyltransferase and ribosome maturation factor
ILMN_2089752	ALKAL2	−0.3669	0.003884	0.049065	−2.95119	Down	ALK and LTK ligand 2
ILMN_1729563	UGDH	−0.36578	0.00195	0.034354	−3.1755	Down	UDP-glucose 6-dehydrogenase
ILMN_1695290	FERMT2	−0.36562	8.34E-06	0.00295	−4.68113	Down	fermitin family member 2
ILMN_1748473	GIMAP4	−0.3654	0.000178	0.010961	−3.88275	Down	GTPase, IMAP family member 4
ILMN_3242038	GPX8	−0.36483	0.000302	0.014133	−3.73508	Down	glutathione peroxidase 8 (putative)
ILMN_1781256	LEFTY2	−0.36409	0.001663	0.031713	−3.2258	Down	left-right determination factor 2
ILMN_1718607	TSPAN4	−0.36243	0.000121	0.009241	−3.98826	Down	tetraspanin 4
ILMN_1653028	COL4A1	−0.36243	0.000251	0.013046	−3.78705	Down	collagen type IV α 1 chain
ILMN_1806403	RASL12	−0.3617	0.000182	0.011037	−3.87729	Down	RAS like family 12
ILMN_1770338	TM4SF1	−0.36154	0.002035	0.035083	−3.16202	Down	transmembrane 4 L six family member 1
ILMN_1757552	CAVIN1	−0.36036	1.87E-05	0.004187	−4.47961	Down	caveolae associated protein 1
ILMN_2148944	ADCY4	−0.36032	0.002039	0.035103	−3.16139	Down	adenylatecyclase 4
ILMN_2346997	RAB23	−0.36006	4.72E-05	0.005816	−4.241	Down	RAB23, member RAS oncogene family
ILMN_1803429	CD44	−0.35802	0.001453	0.029623	−3.268	Down	CD44 molecule (Indian blood group)
ILMN_1757845	SPIRE1	−0.35788	0.000136	0.009769	−3.9577	Down	spire type actin nucleation factor 1
ILMN_2063168	MALL	−0.35738	0.000348	0.014991	−3.69453	Down	mal, T cell differentiation protein like
ILMN_1794492	HOXC6	−0.35691	2.08E-05	0.004478	−4.45271	Down	homeobox C6
ILMN_2089073	ATP9A	−0.35669	0.000323	0.014545	−3.71549	Down	ATPase phospholipid transporting 9A (putative)
ILMN_1676897	HSPA12B	−0.35655	0.001255	0.027297	−3.31351	Down	heat shock protein family A (Hsp70) member 12B
ILMN_1720158	ETS2	−0.35607	1.88E-05	0.004187	−4.47844	Down	ETS proto-oncogene 2, transcription factor
ILMN_1767448	LHFPL6	−0.35579	0.000815	0.022307	−3.44483	Down	LHFPL tetraspan subfamily member 6
ILMN_3238560	IFI27L2	−0.35573	2.7E-05	0.004861	−4.38599	Down	interferon α inducible protein 27 like 2
ILMN_1784871	FASN	−0.35492	1.19E-06	0.001926	−5.14833	Down	fatty acid synthase
ILMN_1680874	TUBB2B	−0.35438	2.27E-05	0.004675	−4.42951	Down	tubulin β 2B class IIb
ILMN_2081682	SMAP2	−0.35276	6.18E-05	0.006575	−4.16954	Down	small ArfGAP2
ILMN_1774982	CDC42EP5	−0.35271	4.66E-05	0.005784	−4.24433	Down	CDC42 effector protein 5
ILMN_1788019	LAMA2	−0.35245	0.00276	0.041163	−3.0638	Down	laminin subunit α 2
ILMN_1783276	NEXN	−0.35153	0.000177	0.010947	−3.88438	Down	nexilin F-actin binding protein
ILMN_1676088	MSRB3	−0.35012	0.000365	0.015248	−3.68065	Down	methionine sulfoxidereductase B3
ILMN_1795183	RNASE1	−0.34959	0.003183	0.044326	−3.01723	Down	ribonuclease A family member 1, pancreatic
ILMN_1704537	PHGDH	−0.34957	8.55E-05	0.007633	−4.08329	Down	phosphoglycerate dehydrogenase
ILMN_1805543	ADAMTS9	−0.34922	0.001219	0.026853	−3.32252	Down	ADAM metallopeptidase with thrombospondin type 1 motif 9
ILMN_2127605	LRP3	−0.3484	9.59E-07	0.001775	−5.19866	Down	LDL receptor related protein 3
ILMN_2410523	DDR2	−0.34789	0.000108	0.008756	−4.019	Down	discoidin domain receptor tyrosine kinase 2
ILMN_2389876	TGFB1I1	−0.34786	0.000225	0.012343	−3.81789	Down	transforming growth factor β 1 induced transcript 1
ILMN_3241758	POTEF	−0.34764	0.001247	0.027181	−3.31554	Down	POTE ankyrin domain family member F
ILMN_1770454	AGRN	−0.34742	0.00053	0.018229	−3.57218	Down	agrin
ILMN_1752249	PIEZO1	−0.34641	0.000798	0.022091	−3.45127	Down	piezo type mechanosensitive ion channel component 1
ILMN_1663080	LFNG	−0.34636	5.26E-05	0.006073	−4.21211	Down	LFNG O-fucosylpeptide 3-β-N-acetylglucosaminyltransferase
ILMN_1672389	CRYZ	−0.3461	0.000537	0.018298	−3.56835	Down	crystallin zeta
ILMN_1776724	LYPD6	−0.34584	1.34E-05	0.00347	−4.56326	Down	LY6/PLAUR domain containing 6
ILMN_1777397	MSX1	−0.34534	0.000377	0.015431	−3.67168	Down	mshhomeobox 1
ILMN_1783593	CCL13	−0.34534	0.001155	0.026177	−3.33892	Down	C-C motif chemokine ligand 13
ILMN_2398159	DKK3	−0.34528	0.003364	0.045581	−2.99896	Down	dickkopf WNT signaling pathway inhibitor 3
ILMN_1743367	FZD4	−0.34522	0.000443	0.016957	−3.62449	Down	frizzled class receptor 4
ILMN_2186061	PFKFB3	−0.34512	0.000537	0.018294	−3.56884	Down	6-phosphofructo-2-kinase/fructose-2,6-biphosphatase 3
ILMN_1662340	ZNF358	−0.34471	3.09E-06	0.002517	−4.9227	Down	zinc finger protein 358
ILMN_1778991	NFIB	−0.34285	0.001613	0.031272	−3.23532	Down	nuclear factor I B
ILMN_1812926	ANTXR2	−0.3415	5.68E-05	0.006347	−4.19218	Down	ANTXR cell adhesion molecule 2
ILMN_1657632	TMEM35B	−0.33999	8.22E-05	0.007511	−4.09378	Down	transmembrane protein 35B
ILMN_1778226	EXTL3	−0.33995	0.001652	0.03162	−3.22796	Down	exostosin like glycosyltransferase 3
ILMN_1782938	SLC16A10	−0.33976	0.002898	0.042362	−3.04796	Down	solute carrier family 16 member 10
ILMN_2193325	MMP23B	−0.33959	0.000713	0.020805	−3.48474	Down	matrix metallopeptidase 23B
ILMN_1739496	PRRX1	−0.33948	0.000146	0.010103	−3.93722	Down	paired related homeobox 1
ILMN_1865764	ZMAT3	−0.33777	1.18E-06	0.001926	−5.14964	Down	zinc finger matrin-type 3
ILMN_1794863	CAMK2N1	−0.33759	0.001093	0.025566	−3.35581	Down	calcium/calmodulin dependent protein kinase II inhibitor 1
ILMN_1735877	EFEMP1	−0.33701	0.003	0.043103	−3.03665	Down	EGF containing fibulin extracellular matrix protein 1
ILMN_2120247	SLC2A10	−0.33689	0.001012	0.02466	−3.37924	Down	solute carrier family 2 member 10
ILMN_1803423	ARHGEF6	−0.33673	0.001997	0.034749	−3.16805	Down	Rac/Cdc42 guanine nucleotide exchange factor 6
ILMN_2201533	TMEM256	−0.3363	9.84E-05	0.008273	−4.04548	Down	transmembrane protein 256
ILMN_1698934	CMTM7	−0.33562	6.8E-05	0.006927	−4.14452	Down	CKLF like MARVEL transmembrane domain containing 7
ILMN_1697409	TNFRSF14	−0.33559	0.000266	0.013333	−3.77075	Down	TNF receptor superfamily member 14
ILMN_1778240	GFOD1	−0.33518	0.00062	0.019425	−3.52642	Down	glucose-fructose oxidoreductase domain containing 1
ILMN_1743373	DLL1	−0.3346	0.000177	0.010936	−3.88539	Down	δ like canonical Notch ligand 1
ILMN_2216582	LYL1	−0.3343	0.000484	0.017744	−3.59922	Down	LYL1 basic helix-loop-helix family member
ILMN_1765641	SEMA3A	−0.33427	0.000485	0.017744	−3.59859	Down	semaphorin 3A
ILMN_1890614	INKA2	−0.33389	7.18E-06	0.00295	−4.71805	Down	inka box actin regulator 2
ILMN_2095133	SPTAN1	−0.33345	7.54E-05	0.007229	−4.11685	Down	spectrin α, non-erythrocytic 1
ILMN_1783681	MRPL34	−0.33294	0.001535	0.030319	−3.25087	Down	mitochondrial ribosomal protein L34
ILMN_1705302	FCGRT	−0.33153	0.00135	0.028444	−3.29084	Down	Fc fragment of IgG receptor and transporter
ILMN_1682738	SMAD3	−0.33045	6.58E-05	0.006811	−4.15297	Down	SMAD family member 3
ILMN_2059535	PPM1F	−0.3304	0.001065	0.025246	−3.36391	Down	protein phosphatase, Mg2+/Mn2+ dependent 1F
ILMN_1790953	TBCB	−0.3301	2.21E-05	0.004675	−4.43726	Down	tubulin folding cofactor B
ILMN_1764788	TNFRSF1B	−0.32976	0.003169	0.044269	−3.0186	Down	TNF receptor superfamily member 1B
ILMN_1754660	ZCCHC24	−0.32898	0.000101	0.008387	−4.03815	Down	zinc finger CCHC-type containing 24
ILMN_2252309	DPP7	−0.32892	0.001126	0.025921	−3.34674	Down	dipeptidyl peptidase 7
ILMN_1674160	BIN1	−0.32855	0.000214	0.012004	−3.83144	Down	bridging integrator 1
ILMN_1675656	PPFIBP2	−0.32771	0.00043	0.016689	−3.63326	Down	PPFIA binding protein 2
ILMN_1728512	YWHAH	−0.32748	6.04E-05	0.006541	−4.17595	Down	tyrosine 3-monooxygenase/tryptophan 5-monooxygenase activation protein eta
ILMN_1789171	EEF2K	−0.32709	1.05E-05	0.003154	−4.62387	Down	eukaryotic elongation factor 2 kinase
ILMN_1680973	FOXF1	−0.3269	0.000111	0.008868	−4.01194	Down	forkhead box F1
ILMN_1805750	IFITM3	−0.3253	0.000765	0.021605	−3.46392	Down	interferon induced transmembrane protein 3
ILMN_2298365	PPP2R2B	−0.3252	0.000614	0.019397	−3.52901	Down	protein phosphatase 2 regulatory subunit Bβ
ILMN_1814194	TCF4	−0.32479	0.00114	0.026051	−3.34287	Down	transcription factor 4
ILMN_1769520	UBE2L6	−0.32451	0.001765	0.032784	−3.20703	Down	ubiquitin conjugating enzyme E2 L6
ILMN_1730487	CALD1	−0.32449	0.000631	0.019631	−3.52115	Down	caldesmon 1
ILMN_1804929	OXTR	−0.32448	0.000803	0.02216	−3.44941	Down	oxytocin receptor
ILMN_2174127	DCBLD2	−0.32399	6.83E-06	0.00295	−4.73008	Down	discoidin, CUB and LCCL domain containing 2
ILMN_1703477	ARHGEF2	−0.32314	7.85E-05	0.007364	−4.10614	Down	Rho/Rac guanine nucleotide exchange factor 2
ILMN_1804277	SPRED1	−0.32305	0.002215	0.036485	−3.13485	Down	sprouty related EVH1 domain containing 1
ILMN_1712075	SYNM	−0.32231	0.002466	0.038662	−3.10033	Down	synemin
ILMN_1770290	CNN2	−0.3223	0.000584	0.01904	−3.54398	Down	calponin 2
ILMN_2064725	METTL7B	−0.32199	0.000739	0.021238	−3.474	Down	methyltransferase like 7B
ILMN_2343097	NCALD	−0.32174	5.26E-06	0.002879	−4.7939	Down	neurocalcin δ
ILMN_1676515	IMPDH1	−0.32151	2.8E-06	0.002369	−4.94609	Down	inosine monophosphate dehydrogenase 1
ILMN_1685540	SHROOM3	−0.32144	9.41E-05	0.008052	−4.05753	Down	shroom family member 3
ILMN_1839019	LPP	−0.32071	0.000295	0.01409	−3.74125	Down	LIM domain containing preferred translocation partner in lipoma
ILMN_1747223	FRYL	−0.32039	0.000312	0.014386	−3.72536	Down	FRY like transcription coactivator
ILMN_1779735	LAMTOR4	−0.32032	0.001384	0.028811	−3.28315	Down	late endosomal/lysosomal adaptor, MAPK and MTOR activator 4
ILMN_2316386	GPBAR1	−0.31949	0.000134	0.009736	−3.96066	Down	G protein-coupled bile acid receptor 1
ILMN_1810559	RHOQ	−0.31922	9.79E-05	0.008252	−4.04665	Down	ras homolog family member Q
ILMN_1804498	BRAT1	−0.31861	0.000157	0.010362	−3.91859	Down	BRCA1 associated ATM activator 1
ILMN_1785618	SMTN	−0.31794	0.000103	0.008513	−4.03259	Down	smoothelin
ILMN_1778444	FKBP5	−0.31765	0.003284	0.045007	−3.00692	Down	FKBP prolylisomerase 5
ILMN_1704154	TNFRSF19	−0.31706	0.000322	0.014545	−3.71667	Down	TNF receptor superfamily member 19
ILMN_2104141	FGD5	−0.317	0.000237	0.012746	−3.80302	Down	FYVE, RhoGEF and PH domain containing 5
ILMN_2149226	CAV1	−0.31687	0.002907	0.042449	−3.04697	Down	caveolin 1
ILMN_1654398	RGL1	−0.31678	0.000409	0.016197	−3.64795	Down	ral guanine nucleotide dissociation stimulator like 1
ILMN_3307892	PARVA	−0.31677	1.15E-05	0.003353	−4.60138	Down	parvin α
ILMN_3241262	PABPC4L	−0.3167	0.001528	0.030233	−3.2524	Down	poly(A) binding protein cytoplasmic 4 like
ILMN_1730229	CGNL1	−0.31553	0.000937	0.02378	−3.40267	Down	cingulin like 1
ILMN_1779071	FEZ1	−0.31546	3.78E-05	0.005357	−4.29902	Down	fasciculation and elongation protein zeta 1
ILMN_1775330	CCDC9B	−0.31511	8.78E-05	0.007748	−4.07615	Down	coiled-coil domain containing 9B
ILMN_1701204	VEGFC	−0.31459	0.000186	0.011194	−3.87116	Down	vascular endothelial growth factor C
ILMN_1777881	TSPAN17	−0.31456	4.57E-05	0.005773	−4.24941	Down	tetraspanin 17
ILMN_1677200	CYFIP2	−0.31396	0.0005	0.017901	−3.58965	Down	cytoplasmic FMR1 interacting protein 2
ILMN_2056032	CD99	−0.3134	0.001609	0.031251	−3.23623	Down	CD99 molecule (Xg blood group)
ILMN_1752591	LEPROTL1	−0.3128	0.001734	0.032382	−3.21268	Down	leptin receptor overlapping transcript like 1
ILMN_1757338	PLSCR4	−0.31123	0.000327	0.014624	−3.7118	Down	phospholipid scramblase 4
ILMN_3245564	ARHGAP44	−0.3111	4.92E-05	0.005917	−4.22987	Down	Rho GTPase activating protein 44
ILMN_1699980	TSPAN18	−0.31001	0.000286	0.013944	−3.74989	Down	tetraspanin 18
ILMN_3232894	CNRIP1	−0.30921	0.00187	0.033574	−3.18876	Down	cannabinoid receptor interacting protein 1
ILMN_1771800	PRKCA	−0.30913	3.17E-05	0.005189	−4.34465	Down	protein kinase C α
ILMN_2397954	PARP3	−0.30902	1.52E-06	0.001955	−5.09076	Down	poly(ADP-ribose) polymerase family member 3
ILMN_1808777	EHD2	−0.30827	0.0001	0.008353	−4.04098	Down	EH domain containing 2
ILMN_1758128	CYGB	−0.30813	3.22E-05	0.005189	−4.34001	Down	cytoglobin
ILMN_1668514	PIP5K1C	−0.30797	0.000323	0.014545	−3.71537	Down	phosphatidylinositol-4-phosphate 5-kinase type 1 γ
ILMN_3236344	BMS1P4	−0.30771	0.000204	0.011804	−3.84512	Down	BMS1 pseudogene 4
ILMN_1708743	NT5DC2	−0.30746	7.44E-05	0.007229	−4.12035	Down	5′-nucleotidase domain containing 2
ILMN_1760493	LIMS2	−0.30721	0.000139	0.00984	−3.95143	Down	LIM zinc finger domain containing 2
ILMN_1736670	PPP1R3C	−0.30647	0.001626	0.031392	−3.23287	Down	protein phosphatase 1 regulatory subunit 3C
ILMN_1671295	CCDC3	−0.3059	0.001513	0.030094	−3.25547	Down	coiled-coil domain containing 3
ILMN_2102330	COL8A2	−0.3057	0.000112	0.008882	−4.01107	Down	collagen type VIII α 2 chain
ILMN_2359945	CES1	−0.30559	0.00016	0.010366	−3.91273	Down	carboxylesterase 1
ILMN_1780057	RENBP	−0.3052	0.000155	0.010361	−3.92141	Down	renin binding protein
ILMN_1658847	NRARP	−0.30411	0.000766	0.021608	−3.46347	Down	NOTCH regulated ankyrin repeat protein
ILMN_1718303	NECTIN2	−0.30407	0.000344	0.014909	−3.69776	Down	nectin cell adhesion molecule 2
ILMN_1764410	GUCD1	−0.30401	0.001226	0.026935	−3.3206	Down	guanylylcyclase domain containing 1
ILMN_1691717	RHBDF2	−0.30355	0.000467	0.017456	−3.60933	Down	rhomboid 5 homolog 2
ILMN_1766675	CDH6	−0.30348	5.65E-05	0.006347	−4.19354	Down	cadherin 6
ILMN_1752046	SH2B3	−0.30284	0.003129	0.044068	−3.02278	Down	SH2B adaptor protein 3
ILMN_1656300	GFRA2	−0.30283	0.000222	0.012233	−3.82121	Down	GDNF family receptor α 2
ILMN_2148459	B2M	−0.30266	0.003487	0.046567	−2.98705	Down	β-2-microglobulin
ILMN_1795639	MGMT	−0.30229	0.000215	0.012004	−3.83117	Down	O-6-methylguanine-DNA methyltransferase
ILMN_1687335	FLNA	−0.30213	0.00045	0.017125	−3.62022	Down	filamin A
ILMN_2049536	TRPV2	−0.30191	5E-05	0.005957	−4.22548	Down	transient receptor potential cation channel subfamily V member 2
ILMN_1668721	CCND3	−0.30153	0.003431	0.046061	−2.99245	Down	cyclin D3
ILMN_1801226	DOCK6	−0.30138	0.000109	0.008759	−4.01846	Down	dedicator of cytokinesis 6
ILMN_3238196	CYTH4	−0.30118	0.001775	0.0328	−3.20526	Down	cytohesin 4
ILMN_1760667	POLR3GL	−0.30114	0.000115	0.009	−4.00214	Down	RNA polymerase III subunit G like
ILMN_2367707	PKN1	−0.30078	1.76E-05	0.004057	−4.49419	Down	protein kinase N1
ILMN_1756439	SCRN1	−0.30064	2.03E-05	0.004408	−4.45788	Down	secernin 1
ILMN_1746704	TRIM8	−0.30043	1.71E-06	0.001955	−5.06366	Down	tripartite motif containing 8
ILMN_1727043	COLGALT1	−0.30041	1.61E-06	0.001955	−5.0774	Down	collagen β(1-O)galactosyltransferase 1
ILMN_1789639	FMOD	−0.29998	0.000703	0.020621	−3.48914	Down	fibromodulin
ILMN_1759513	RND3	−0.2987	0.00056	0.018724	−3.5563	Down	Rho family GTPase 3
ILMN_2339294	LILRB5	−0.29854	0.000286	0.013933	−3.75069	Down	leukocyte immunoglobulin like receptor B5
ILMN_2205896	MEIS3P1	−0.29849	3.51E-05	0.005215	−4.31774	Down	Meishomeobox 3 pseudogene 1
ILMN_1677404	RAP2A	−0.29848	1.01E-05	0.003119	−4.63287	Down	RAP2A, member of RAS oncogene family
ILMN_1716678	NPC2	−0.29813	0.000259	0.013173	−3.77819	Down	NPC intracellular cholesterol transporter 2
ILMN_1686555	FYN	−0.29766	6.74E-05	0.006914	−4.14687	Down	FYN proto-oncogene, Src family tyrosine kinase
ILMN_1853824	MGAT3	−0.29749	0.000891	0.023304	−3.4178	Down	β-1,4-mannosyl-glycoprotein 4-β-N-acetylglucosaminyltransferase
ILMN_1661264	SHMT2	−0.29747	0.000466	0.017456	−3.61015	Down	serine hydroxymethyltransferase 2
ILMN_1748206	CCM2L	−0.29721	0.001703	0.032137	−3.21833	Down	CCM2 like scaffold protein
ILMN_1724480	AXIN2	−0.29689	0.000111	0.008868	−4.01392	Down	axin 2
ILMN_1776157	SEPTIN4	−0.29638	0.000337	0.014847	−3.70339	Down	septin 4
ILMN_1775734	SH2D3C	−0.29607	0.001157	0.02618	−3.33856	Down	SH2 domain containing 3C
ILMN_1761159	ESYT1	−0.29576	9.88E-06	0.003091	−4.63908	Down	extended synaptotagmin 1
ILMN_1770824	ARHGAP4	−0.29525	0.000371	0.015365	−3.67562	Down	Rho GTPase activating protein 4
ILMN_1713732	ABL1	−0.29499	1.21E-05	0.003404	−4.58941	Down	ABL proto-oncogene 1, non-receptor tyrosine kinase
ILMN_1687440	HIPK2	−0.2942	0.001881	0.033734	−3.1869	Down	homeodomain interacting protein kinase 2
ILMN_1694539	MAP3K6	−0.29418	7.48E-05	0.007229	−4.11899	Down	mitogen-activated protein kinase kinasekinase 6
ILMN_2381697	P4HA2	−0.29305	0.000174	0.010872	−3.88914	Down	prolyl 4-hydroxylase subunit α 2
ILMN_1803348	EHBP1	−0.29305	0.000173	0.010825	−3.89106	Down	EH domain binding protein 1
ILMN_1776464	PARP4	−0.29295	4.71E-05	0.005816	−4.24138	Down	poly(ADP-ribose) polymerase family member 4
ILMN_1671404	SVIL	−0.29228	0.00024	0.012746	−3.79968	Down	supervillin
ILMN_1769118	SEPTIN9	−0.29218	0.000231	0.012551	−3.81002	Down	septin 9
ILMN_2315789	PTPRD	−0.292	0.000226	0.012397	−3.81635	Down	protein tyrosine phosphatase receptor type D
ILMN_2401978	STAT3	−0.29197	2.58E-06	0.002346	−4.96523	Down	signal transducer and activator of transcription 3
ILMN_1785424	ABLIM1	−0.29182	0.002923	0.042546	−3.04516	Down	actin binding LIM protein 1
ILMN_2082585	SNAI2	−0.29137	0.000937	0.02378	−3.40282	Down	snail family transcriptional repressor 2
ILMN_1809850	RCN3	−0.2896	0.000133	0.009736	−3.96254	Down	reticulocalbin 3
ILMN_3246065	CCDC151	−0.28935	0.000122	0.009241	−3.98798	Down	coiled-coil domain containing 151
ILMN_1745806	PEMT	−0.28934	0.000161	0.010372	−3.91073	Down	phosphatidylethanolamine N-methyltransferase
ILMN_1791226	NXN	−0.28912	6.77E-05	0.006914	−4.14567	Down	nucleoredoxin
ILMN_1758315	SLC9A9	−0.28902	0.000259	0.013173	−3.77817	Down	solute carrier family 9 member A9
ILMN_1661194	CLDN14	−0.28826	0.000466	0.017456	−3.61005	Down	claudin 14
ILMN_3225591	RPL14	−0.28806	0.000665	0.020117	−3.50549	Down	ribosomal protein L14
ILMN_2232712	MYO10	−0.28774	0.001061	0.02524	−3.36484	Down	myosin X
ILMN_1913060	CMKLR1	−0.28763	0.00032	0.014545	−3.71847	Down	chemerin chemokine-like receptor 1
ILMN_1789502	GPC4	−0.28702	0.001807	0.033091	−3.19966	Down	glypican 4
ILMN_2047599	TMEM50B	−0.28576	0.001003	0.024564	−3.38214	Down	transmembrane protein 50B
ILMN_1719543	MAF	−0.28566	0.000438	0.016832	−3.62816	Down	MAF bZIP transcription factor
ILMN_1748625	TCEAL4	−0.2856	0.000494	0.01788	−3.59303	Down	transcription elongation factor A like 4
ILMN_1670134	FADS1	−0.28515	0.000296	0.01409	−3.74097	Down	fatty acid desaturase 1
ILMN_1660871	NEK6	−0.28507	0.000893	0.023304	−3.41739	Down	NIMA related kinase 6
ILMN_1674385	YWHAQ	−0.28455	2.86E-05	0.004971	−4.37044	Down	tyrosine 3-monooxygenase/tryptophan 5-monooxygenase activation protein theta
ILMN_1681515	CRLF1	−0.2833	0.002663	0.04025	−3.07546	Down	cytokine receptor like factor 1
ILMN_1782086	AOC3	−0.28296	0.000461	0.01734	−3.61347	Down	amine oxidase copper containing 3
ILMN_1768110	MAP3K20	−0.28275	0.00124	0.027121	−3.31715	Down	mitogen-activated protein kinase kinasekinase 20
ILMN_1733538	RGS10	−0.28222	0.001065	0.025246	−3.36393	Down	regulator of G protein signaling 10
ILMN_2185884	DHRS4	−0.28207	0.001328	0.028154	−3.29599	Down	dehydrogenase/reductase 4
ILMN_2381899	OPTN	−0.28193	0.000301	0.014133	−3.73615	Down	optineurin
ILMN_3202024	FTL	−0.28175	0.00324	0.044809	−3.01128	Down	ferritin light chain
ILMN_1815500	ITPR3	−0.28174	0.002482	0.038752	−3.09823	Down	inositol 1,4,5-trisphosphate receptor type 3
ILMN_1684554	COL16A1	−0.28149	0.001102	0.025658	−3.35341	Down	collagen type XVI α 1 chain
ILMN_1709590	PGM5	−0.28126	0.000681	0.020348	−3.49848	Down	phosphoglucomutase 5
ILMN_1669033	NCOA1	−0.28125	2.76E-05	0.00487	−4.37959	Down	nuclear receptor coactivator 1
ILMN_1913678	IRAK3	−0.28034	0.002099	0.035634	−3.15202	Down	interleukin 1 receptor associated kinase 3
ILMN_1815745	SOX4	−0.27903	4.4E-05	0.005755	−4.25887	Down	SRY-box transcription factor 4
ILMN_3236858	NYNRIN	−0.2784	4.66E-05	0.005784	−4.24391	Down	NYN domain and retroviral integrase containing
ILMN_1800634	NME4	−0.27818	0.000791	0.022033	−3.4539	Down	NME/NM23 nucleoside diphosphate kinase 4
ILMN_1714170	SPSB1	−0.27787	0.001682	0.031939	−3.22231	Down	splA/ryanodine receptor domain and SOCS box containing 1
ILMN_1698725	FRMD3	−0.27757	0.002954	0.042707	−3.04173	Down	FERM domain containing 3
ILMN_1695946	TRNP1	−0.27716	1.26E-05	0.003423	−4.57872	Down	TMF1 regulated nuclear protein 1
ILMN_1654065	ATOH8	−0.27686	0.001152	0.02613	−3.33982	Down	atonal bHLH transcription factor 8
ILMN_1739885	SLC41A3	−0.27657	2.16E-07	0.000867	−5.53926	Down	solute carrier family 41 member 3
ILMN_1693826	HAVCR2	−0.27451	0.003493	0.046607	−2.98647	Down	hepatitis A virus cellular receptor 2
ILMN_1775931	EPHA3	−0.27402	0.002521	0.039049	−3.09318	Down	EPH receptor A3
ILMN_1659206	RARA	−0.27351	0.00249	0.038808	−3.09718	Down	retinoic acid receptor α

### Gene ontology and pathway enrichment of DEGs analysis

To clarify the major functions of these DEGs, we first explored the associated biological processes and REACTOME pathways. The top highly enriched GO terms were divided into three categories: biological process (BP), cellular component (CC) and molecular function (MF) and are listed in Table 3. The most enriched GO terms in BP was reproduction, macromolecule catabolic process, cell adhesion and localization of cell, that in CC was nuclear outer membrane–endoplasmic reticulum membrane network, Golgi apparatus, supramolecular complex and cell junction, and that in MF had identical protein binding, molecular function regulator, signaling receptor binding and molecular function regulator. In the REACTOME pathway enrichment analysis, the DEGs were mostly enriched in cell surface interactions at the vascular wall, epigenetic regulation of gene expression, extracellular matrix organization and axon guidance and are listed in Table 4.

**Table 3:** The enriched GO terms of the up- and down-regulated DEGs

GO ID	CATEGORY	GO Name	P Value	FDR B and H	FDR B and Y	Bonferroni	Gene count	Gene
**Up-regulated genes**								
GO:0000003	BP	reproduction	1.54E-05	1.92E-02	1.75E-01	7.69E-02	59	CEBPB, GRHL2, ACSL4, S100A11, KMT2C, UBE2A, TESK2, AGFG1, AHR, HES1, MAFF, HSD11B2, PAQR7, RHOBTB3, NECTIN3, CRH, SLC4A2, STS, CSNK2A2, SLC22A5, GABRB1, PLAC1, SPIRE2, PSG1, PSG2, ING2, PSG3, INHA, PSG4, PSG5, PSG6, PSG7, PSG9, CYP11A1, PSG11, CYP19A1, YTHDC1, SEPTIN6, STAT5B, CREBRF, NFE2, SPIN1, STRA6, MBD2, DDR1, TBX3, CAST, CGB7, TEAD3, LHB, GMCL1, OVGP1, TIAL1, EGFR, INSL6, LNPEP, TLR3, TMF1, PLEKHA1
GO:0009057	BP	macromolecule catabolic process	5.89E-04	1.28E-01	1.00E+00	1.00E+00	48	CARHSP1, RPS13, WAC, UBE2A, UBE2D3, CLN3, UCHL3, LYPLA1, HSPB1, HSP90AA1, AMFR, DCP2, IDS, AZIN1, IGF2BP3, IGF2BP2, GET4, CSNK2A2, MTM1, NCAN, TNKS1BP1, USP33, TRIM25, PSMA3, PSMC4, DDB1, CREBRF, PTPN3, STX5, FBXO9, SMG9, PJA1, USP27X, RBX1, RBBP6, CAST, RDX, PELI1, NCCRP1, STT3B, RYBP, OVGP1, EGFR, TIMP2, LNPEP, PKP3, FURIN, TMF1
GO:0042175	CC	nuclear outer membrane- endoplasmic reticulum membrane network	1.06E-03	1.69E-01	1.00E+00	6.75E-01	38	SPTLC3, CDS1, BET1, ACSL4, SPCS3, PIGH, EVA1A, CLN3, HSD3B1, ULK1, HSD11B2, FKBP2, AMFR, FOLR1, SPTLC1, RAB3GAP1, STS, NSG1, GDPD1, GPAA1, CYP19A1, STX5, BCAP29, NUP153, TOR1AIP2, CAMK2G, NUCB2, CASP4, TMED4, STT3B, RASGRP1, MFSD2A, SPPL2A, EGFR, LPCAT3, FURIN, TLR3, PAFAH2
GO:0005794	CC	Golgi apparatus	1.36E-02	3.09E-01	1.00E+00	1.00E+00	46	SGSM1, CNST, BET1, SPG21, ST3GAL6, CLN3, AMFR, ANK3, FOLR1, GDF15, YIPF4, RHOBTB3, ST3GAL4, RAB3GAP1, STS, NCAN, NSG1, PDE4DIP, RAB11FIP5, FHDC1, TBC1D23, STX11, CSGALNACT1, ECE2, USP33, ACER2, ATP6V0C, ING2, STK26, C1GALT1, COG3, BMP1, STX5, AP1G2, TAF7, GOLGA4, YIPF6, NUCB2, TMED4, LHB, RASGRP1, EGFR, FURIN, TLR3, TMF1, ELF3
GO:0042802	MF	identical protein binding	4.89E-03	2.96E-01	1.00E+00	1.00E+00	59	CEBPB, IER5, TWIST1, S100A11, S100P, H2BC8, H2BC6, H2BC4, TLE5, CLK3, AHR, UCK2, TRPV6, HES1, HOOK2, ULK1, HSPB1, CAP2, HSP90AA1, AMFR, GDF15, ANXA4, SRR, CLDN7, NECTIN3, CGGBP1, TBK1, GRAMD2B, ATF3, RIPOR2, STK26, NAB2, DAPK1, BMP1, JUP, DHPS, SMG9, KYNU, HSPB8, NUP153, YIPF6, CAMK2G, DYRK1A, GRB7, RDX, UBA3, TDG, CLDN8, TFRC, GMCL1, RASGRP1, SPPL2A, EGFR, CBX5, ENTPD1, TLR3, PAK1, PAK2, SPATA13
GO:0098772	MF	molecular function regulator	1.03E-02	2.96E-01	1.00E+00	1.00E+00	55	SGSM1, NET1, PDPK1, DENND2D, SERPINI1, AGFG1, TNFAIP8, HSPB1, RASAL2, HSP90AA1, DNAJB1, FNTA, GDF15, ANXA4, CRH, AZIN1, RAB3GAP1, CSH2, PPP4R2, ATP1B3, DEPDC1B, RIPOR2, INHA, MYO9A, INSL4, GH2, BMP1, PTPN3, CGB1, SEMA3B, RGL2, TOR1AIP2, RGPD8, PPP1R14C, TFPI2, FBRS, CAST, CGB7, COX17, PPP1R14B, ITIH5, LHB, RASGRP1, EEF1B2, GRTP1, FAM13A, EGFR, INSL6, TIMP2, FURIN, TLR3, PAK2, MARK2, ENSA, SPATA13
**Down-regulated genes**								
GO:0007155	BP	cell adhesion	4.46E-28	8.58E-25	7.92E-24	2.57E-24	104	CLDN14, ABL1, ENPP2, VSIG4, EMILIN2, LAMC3, PGM5, NRARP, FZD4, SLIT2, MFAP4, MFGE8, FAT1, FBLN2, MYL9, CD99, SPON1, JAG1, GPC4, ANTXR1, SRPX, VCAM1, VCL, FOXF1, COL1A1, PMP22, VEGFC, COL3A1, COL5A1, COL6A1, COL6A2, FLNA, COL6A3, COL8A2, FBLN5, VTN, VWF, COL16A1, ABI3BP, PODXL, FOLR2, EGFL6, CYFIP2, LIMS2, PDPN, RND3, IGFBP2, CCM2L, IGFBP7, FEZ1, PPM1F, VCAN, PARVA, SNAI2, AOC3, PRKCA, CCN2, EGFLAM, EPDR1, FYN, NEXN, MYADM, MYO10, AXL, ADAM15, PIEZO1, TNFRSF14, GAS6, ITGA1, TNFRSF21, LYVE1, PTPRD, ADAMTS9, ACKR3, WNT3A, NECTIN2, FERMT2, RARA, SH2B3, DDR2, LAMA2, LAMA4, LAMB2, LAMC1, CAV1, TEK, DLL1, PIP5K1C, LGALS1, TGFB1I1, TGFBI, HAVCR2, COL18A1, THY1, FAM107A, CD36, LPP, CD44, CD81, CLDN5, CDH6, CDH11, SMAD3, EPHA3
GO:0051674	BP	localization of cell	2.02E-23	1.67E-20	1.54E-19	1.17E-19	111	ABL1, ABR, MAP1B, APCDD1, PDGFRB, ENPP2, MATN2, ACTA2, CYGB, TMIGD3, SOX18, LAMC3, F3, ADCY3, SEMA3A, SLIT2, FAT1, MGAT3, EFEMP1, CD99, JAG1, AGTR1, GPC4, CCL13, CMKLR1, CNN2, VCAM1, VCL, FOXF1, COL1A1, COL1A2, PMP22, VEGFC, COL3A1, COL5A1, FLNA, DDIT4, VTN, ADAMTS1, PODXL, FOLR2, CXCL14, ADGRA2, PDPN, RND3, ARHGAP4, IGFBP3, PRCP, PPM1F, VCAN, CSPG4, SLIT3, PARVA, SNAI2, AOC3, PRKCA, PKN1, CCN2, EGFLAM, CAVIN1, FSCN1, FYN, NEXN, HYAL2, PROS1, MYADM, SPARC, MYO10, AXL, ADAM15, TNFRSF14, GAS6, TUBB2B, NDN, ITGA1, DCN, STAT3, STC1, ADAMTS9, BST2, ACKR3, TSPO, RIPOR1, BRAT1, RAP2A, RARRES2, CD248, GPER1, DDR2, LAMA2, LAMA4, LAMB2, LAMC1, CAV1, TEK, PIP5K1C, LPAR1, LDB2, COL18A1, ARAP3, THY1, LMNA, FAM107A, TIMP1, ATOH8, CD44, SPRED1, CD81, ARHGEF2, SMAD3, EPHA3
GO:0099080	CC	supramolecular complex	3.08E-13	1.81E-11	1.24E-10	1.63E-10	77	TPM2, CYBRD1, MAP1B, ACTA2, ACTG2, TUBB2A, PGM5, TBCB, MFAP4, PARP4, FAT1, MYL9, PEMT, ANTXR1, CCDC151, CNN2, VCAM1, VCL, COL1A1, COL1A2, COL3A1, COL4A1, COL4A2, COL4A5, COL5A1, COL5A2, COL6A1, FLNA, COL6A3, BIN1, SHROOM3, FBLN5, SEPTIN4, PODXL, NCKAP5L, ADGRA2, SMTN, PDPN, ARHGAP4, FEZ1, PARVA, AOC3, FSCN1, FYN, NEXN, HYAL2, NES, MYH11, NEK6, MYO10, SEPTIN9, SPTAN1, TUBB2B, AIF1L, DCN, PTH1R, ITPR3, PDLIM3, SVIL, FERMT2, DPYSL2, CALD1, GPER1, SYNM, TEK, RHOQ, ABLIM1, OXTR, LMNA, FAM107A, FARP1, CD36, LPP, CD44, PALM, ARHGEF2, TNNT3
GO:0030054	CC	cell junction	3.95E-13	2.09E-11	1.43E-10	2.09E-10	71	CLDN14, MAP1B, PDGFRB, PGM5, FZD4, FAT1, CAMK2N1, CD99, JAG1, FGFR3, CNN2, ARHGEF6, VCL, PMP22, FLNA, SHROOM3, PODXL, YWHAH, CYFIP2, LIMS2, PDPN, RND3, CSPG4, PARVA, PRKCA, EGFLAM, FSCN1, NEXN, RAB23, MYADM, LYPD6, ADAM15, SPTAN1, B2M, CCND1, AIF1L, ITGA1, RASIP1, GJA4, PDLIM3, SYT11, ARHGAP44, SVIL, NECTIN2, YWHAQ, FERMT2, GPER1, SYNM, DDR2, LASP1, CAV1, CAV2, TEK, DLL1, PIP5K1C, TGFB1I1, HAVCR2, OXTR, THY1, FAM107A, FARP1, LPP, CD44, TSPAN4, CD81, CLDN5, CGNL1, ARHGEF2, AGRN, CDH6, CDH11
GO:0005102	MF	signaling receptor binding	6.59E-08	6.11E-06	4.59E-05	6.72E-05	75	CMTM3, ABL1, CRLF1, PDGFRB, ETS2, SEMA3A, SLIT2, MFGE8, EFEMP1, JAG1, AGTR1, CCL13, LTBP4, VCAM1, VEGFC, COL3A1, COL5A1, FLNA, FBLN5, VTN, VWF, COL16A1, CNRIP1, CXCL14, ALKAL2, YWHAH, EGFL6, PDPN, IGFBP2, IGFBP4, SLIT3, NCOA1, PRKCA, PKN1, CCN2, FYN, DKK3, HYAL2, NES, ADAM15, B2M, GAS6, ITGA1, STAT3, STC1, PXDN, PTPRD, WNT3A, PLSCR4, RARA, RARRES2, SH2B3, SH2D3C, LAMA2, LAMA4, LAMB2, CAV1, CAV2, DLL1, TGFB1I1, TGFB3, LEFTY2, TGFBI, RSPO3, ANGPTL2, THY1, TIMP1, CD36, CD44, TSPAN4, PALM, SPRED1, CD81, CMTM7, SMAD3
GO:0098772	MF	molecular function regulator	2.41E-05	1.12E-03	8.38E-03	2.46E-02	69	CMTM3, CRLF1, ABR, CTSC, PPP1R14A, SEMA3A, SLIT2, CAMK2N1, EFEMP1, JAG1, CCL13, SIPA1L2, DOCK6, SMAP2, ARHGEF6, VEGFC, FLNA, COL6A3, WARS1, CXCL14, ALKAL2, YWHAH, PPP1R3C, TXNIP, ARHGAP4, PPP2R2B, IGFBP3, LAMTOR4, CCN2, GPSM1, DKK3, HYAL2, CYTH4, PROS1, LYPD6, RAPGEF5, GAS6, CCND1, INKA2, STC1, RGL1, AFAP1L2, PXDN, BST2, WNT3A, ARHGAP44, RIPOR1, FGD5, RAB3IL1, RARA, SH2D3C, WFDC1, RENBP, CAV1, RGS10, CCND2, CCND3, TGFB3, LEFTY2, ARAP3, THY1, TIMP1, ABCE1, FARP1, SPRED1, ARHGEF2, CMTM7, AGRN, PCOLCE

BP, CC and MF.

**Table 4:** The enriched pathway terms of the up- and down-regulated DEGs

Pathway ID	Pathway Name	P-value	FDR B& H	FDR B&Y	Bonferroni	Gene Count	Gene
**Up-regulated genes**							
1269373	Cell surface interactions at the vascular wall	2.86E-05	4.73E-03	3.47E-02	2.46E-02	13	PSG8, SLC3A2, ATP1B3, PSG1, PSG2, PSG3, PSG4, PSG5, PSG6, PSG7, PSG9, PSG11, GRB7
1269734	Epigenetic regulation of gene expression	8.47E-04	2.18E-02	1.60E-01	7.28E-01	11	H2AC6, H2BC8, H2BC6, H2BC4, H2BC21, H4C8, H2BC12, MBD2, TAF1B, TDG, H2BC5
1270001	Metabolism of lipids and lipoproteins	1.33E-03	2.87E-02	2.10E-01	1.00E+00	33	OLAH, SPTLC3, CDS1, ACADVL, ACOX3, ACSL4, AHR, PIP5K1B, HSD3B1, TNFAIP8, HSD11B2, SH3KBP1, SPTLC1, TNFAIP8L1, PLEKHA6, STS, CSNK2A2, MTM1, ACER2, GDPD1, CYP11A1, CYP19A1, BMP1, TEAD3, LHB, MFSD2A, MTMR4, ABHD5, LPCAT3, FURIN, AKR1B15, PLEKHA1, RORA
1268701	Post-translational protein modification	3.57E-03	6.02E-02	4.41E-01	1.00E+00	38	BET1, WAC, H2AC6, H2BC8, UBE2A, H2BC6, H2BC4, UBE2D3, H2BC21, ST3GAL6, PIGH, UCHL3, ADAMTSL4, H2BC12, AMFR, ANK3, FOLR1, ST3GAL4, STS, BABAM1, USP33, TRIM25, GPAA1, INO80C, PSMA3, PSMC4, C1GALT1, COG3, H2AW, STX5, DHPS, NUP153, RAB25, GNE, TDG, LHB, FURIN, H2BC5
1268677	Metabolism of proteins	1.06E-02	1.30E-01	9.49E-01	1.00E+00	52	ACADVL, BET1, RPS13, WAC, H2AC6, SPCS3, H2BC8, UBE2A, H2BC6, H2BC4, UBE2D3, H2BC21, ST3GAL6, PIGH, H4C8, UCHL3, ADAMTSL4, H2BC12, AMFR, ANK3, FOLR1, DCP2, ST3GAL4, STS, BABAM1, CSNK2A2, ATF3, ATF4, CGB5, USP33, CHCHD10, TRIM25, GPAA1, INO80C, INHA, PSMA3, PSMC4, C1GALT1, COG3, H2AW, STX5, GNG12, DHPS, NUP153, RAB25, GNE, COX17, TDG, LHB, EEF1B2, FURIN, H2BC5
1270302	Developmental Biology	1.79E-02	1.92E-01	1.00E+00	1.00E+00	36	CEBPB, RPS6KA5, PFN2, KMT2C, H2AC6, H2BC8, H2BC6, H2BC4, H2BC21, H4C8, HES1, H2BC12, RASAL2, CAP2, HSP90AA1, ANK3, KRT23, CSNK2A2, NCAN, PSMA3, PSMC4, JUP, KAZN, KRTAP26-1, RBX1, CAMK2G, GRB7, RDX, RASGRP1, TCHH, EGFR, PKP3, FURIN, PAK1, PAK2, H2BC5
**Down-regulated genes**							
1270244	Extracellular matrix organization	1.93E-23	1.40E-20	1.00E-19	1.40E-20	46	LAMC3, MFAP4, FBLN2, EFEMP1, PLOD1, LTBP4, VCAM1, COL1A1, COL1A2, COL3A1, COL4A1, COL4A2, COL4A5, COL5A1, COL5A2, COL6A1, COL6A2, COL6A3, COL8A2, FBLN5, VTN, FMOD, COL16A1, ADAMTS1, EFEMP2, VCAN, PRKCA, SPARC, ADAM15, ITGA1, DCN, ADAMTS9, P4HA2, DDR2, LAMA2, LAMA4, LAMB2, LAMC1, COLGALT1, TGFB3, COL18A1, TIMP1, CD44, LTBP2, AGRN, PCOLCE
1270303	Axon guidance	3.15E-05	1.35E-03	9.67E-03	2.29E-02	31	ABL1, PDGFRB, RPS6KA2, SEMA3A, SLIT2, MYL9, FGFR3, VCL, COL4A1, COL4A2, COL4A5, COL6A1, COL6A2, COL6A3, VWF, SLIT3, PRKCA, FYN, MYH11, MYO10, SPTAN1, ITGA1, GFRA2, DPYSL2, LAMC1, TEK, PIP5K1C, ABLIM1, SPRED1, AGRN, EPHA3
1269478	Signaling by PDGF	2.76E-04	7.15E-03	5.13E-02	2.00E-01	22	PDGFRB, ADCY3, FGFR3, VCL, COL4A1, COL4A2, FOXO1, COL4A5, COL6A1, COL6A2, COL6A3, ADCY4, VWF, PRKCA, FYN, SPTAN1, GFRA2, STAT3, ITPR3, TEK, PIP5K1C, SPRED1
1269340	Hemostasis	1.83E-03	3.51E-02	2.51E-01	1.00E+00	29	ABL1, PDE9A, F3, EHD2, CD99, DOCK6, VCL, VEGFC, FLNA, VWF, PDPN, PRCP, PRKCA, FYN, PROS1, SPARC, GAS6, ITGA1, ITPR3, CFD, RARRES2, SH2B3, CAV1, TEK, TGFB3, LEFTY2, TIMP1, CD36, CD44
1270302	Developmental Biology	6.14E-03	8.76E-02	6.28E-01	1.00E+00	41	ABL1, PDGFRB, RPS6KA2, SEMA3A, SLIT2, MYL9, FGFR3, VCL, COL4A1, COL4A2, FOXO1, COL4A5, COL6A1, COL6A2, COL6A3, VWF, SLIT3, NCOA1, PRKCA, FYN, MYH11, MYO10, SPTAN1, ITGA1, GFRA2, STAT3, TCF4, DPYSL2, RARA, LAMC1, EBF1, TEK, PIP5K1C, CCND3, LEFTY2, ABLIM1, CD36, SPRED1, AGRN, SMAD3, EPHA3
1269310	Cytokine Signaling in Immune system	6.57E-03	9.18E-02	6.58E-01	1.00E+00	31	CRLF1, UBE2L6, PDGFRB, IFITM3, FGFR3, VCAM1, VCL, COL1A2, FOXO1, VWF, IFITM2, IFNGR2, FSCN1, FYN, SPTAN1, B2M, TNFRSF14, CCND1, NDN, GFRA2, STAT3, BST2, TRIM8, TEK, HAVCR2, TIMP1, CD36, CD44, SPRED1, IRAK3, TNFRSF1B

### PPI network establishment and modules selection

**Figure 3:**
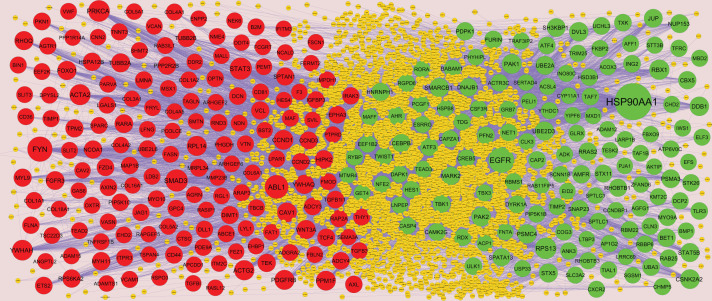
PPI network of DEGs The PPI network of DEGs was constructed using Cytoscape. Up-regulated genes are marked in green; down-regulated genes are marked in red.

**Figure 4:**
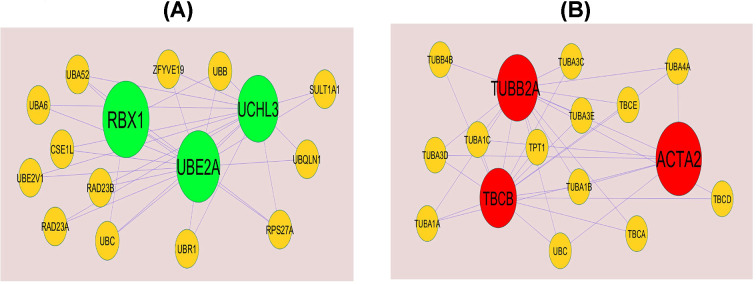
Modules of isolated form PPI of DEGs (**A**) The most significant module was obtained from PPI network with 16 nodes and 32 edges for up-regulated genes. (**B**) The most significant module was obtained from PPI network with 16 nodes and 34 edges for down-regulated genes. Up-regulated genes are marked in green; down-regulated genes are marked in red.

By using the STRING database, the PPI network of DEGs was established and consisted of 4687 nodes and 11236 edges (Figure 3). A total of ten hub genes were selected for key biomarker identification and are listed in Table 5. They consisted of five up-regulated genes (*HSP90AA1*, *EGFR*, *RPS13*, *RBX1* and *PAK1*) and five down-regulated genes (*FYN*, *ABL1*, *SMAD3*, *STAT3* and *PRKCA*). Then PEWCC1 was used to find clusters in the network. Four modules were calculated according to k-core = 2. Among them, module 1 contained 16 nodes and 32 edges, with the highest score (Figure 4A) and module 2 contained 16 nodes and 34 edges (Figure 4B). We performed the functional analysis for the top 2 modules. In functional enrichment analysis, the DEGs of module 1 were mostly enriched in post-translational protein modification, developmental biology and macromolecule catabolic process; the DEGs of module 2 in supramolecular complex and localization of cell.

**Table 5:** Topology table for up- and down-regulated genes

Regulation	Node	Degree	Betweenness	Stress	Closeness
Up	HSP90AA1	655	0.22721	81351654	0.412863
Up	EGFR	324	0.081831	21358048	0.396682
Up	RPS13	176	0.040553	21024400	0.332742
Up	RBX1	132	0.02978	9542636	0.3408
Up	PAK1	115	0.016755	4561194	0.37158
Up	CSNK2A2	112	0.026157	6172590	0.354758
Up	PAK2	107	0.012051	4334042	0.349858
Up	DDB1	105	0.024917	6240978	0.340602
Up	PSMC4	101	0.019368	3810802	0.348739
Up	DVL3	99	0.017256	5360158	0.344762
Up	UBE2D3	96	0.018158	6872298	0.333975
Up	SMARCB1	90	0.021042	7085456	0.333215
Up	STAT5B	89	0.005633	1782886	0.349937
Up	STX5	86	0.021729	5783250	0.327692
Up	UBE2A	86	0.01665	6800442	0.330652
Up	NUP153	81	0.019493	3839268	0.33388
Up	JUP	79	0.012614	3875882	0.320148
Up	PSMA3	78	0.009276	2735940	0.330535
Up	SH3KBP1	76	0.011315	2648130	0.345321
Up	HSPB1	69	0.012593	3676246	0.34167
Up	BET1	67	0.013941	4543480	0.325711
Up	AMFR	66	0.018899	2411936	0.345907
Up	RRAS2	64	0.00438	2166928	0.301914
Up	MARK2	63	0.012107	3287228	0.338315
Up	CBX5	62	0.015481	3171408	0.327807
Up	CEBPB	62	0.007601	2245984	0.350172
Up	PDPK1	62	0.005919	1612918	0.342169
Up	HNRNPH1	59	0.013608	2423406	0.335625
Up	DNAJB1	58	0.00611	1362926	0.344205
Up	ATF3	57	0.005226	1555506	0.352755
Up	SPATA13	56	0.00717	2217896	0.295759
Up	FURIN	55	0.017306	2333572	0.332317
Up	RHOBTB1	54	0.005265	891404	0.309798
Up	SNAP23	52	0.010301	2133980	0.326551
Up	STX11	49	0.006356	1012388	0.276999
Up	TBK1	48	0.00946	1612462	0.346521
Up	RAB25	48	0.00796	4209932	0.282579
Up	ING2	42	0.006848	2338492	0.325236
Up	ULK1	42	0.007643	7120856	0.274292
Up	UCHL3	42	0.007629	1994146	0.325779
Up	PFN2	41	0.004889	1200798	0.329235
Up	CAP2	40	0.002011	661156	0.288156
Up	UBA3	40	0.005749	1625200	0.326187
Up	DYRK1A	40	0.004394	1121650	0.309634
Up	RBM22	39	0.01146	1346380	0.322616
Up	TXK	38	0.001513	482866	0.300558
Up	TAF7	38	0.010147	1476134	0.322972
Up	CAMK2G	38	0.007152	1050770	0.341969
Up	ATF4	37	0.006742	1255258	0.328036
Up	TLR3	36	0.004686	1054636	0.305217
Up	MYO9A	36	8.05E-04	406980	0.28752
Up	BABAM1	36	0.008431	1423346	0.324044
Up	STK26	36	0.008804	1319200	0.323842
Up	ACTR3C	35	0.002617	659206	0.282732
Up	CREB5	35	0.001154	566084	0.298035
Up	NET1	33	0.003076	787876	0.327189
Up	EEF1B2	32	0.00465	1484558	0.32501
Up	AHR	32	0.003156	847220	0.347858
Up	PIP5K1B	31	0.00183	957308	0.326391
Up	CLN3	31	0.007159	992822	0.323485
Up	HES1	30	0.0035	796872	0.300115
Up	RBBP6	29	0.008094	1087968	0.324089
Up	AP1G2	29	0.008892	933344	0.321377
Up	RDX	29	0.003378	953914	0.325146
Up	RGPD8	28	0.002967	626564	0.32295
Up	HSD3B1	28	0.009418	6545696	0.230497
Up	COG3	27	0.00261	458374	0.24988
Up	ATP6V0C	27	0.008472	953284	0.320959
Up	TFRC	27	0.004398	847110	0.329004
Up	TIAL1	27	0.005954	1083682	0.323485
Up	DCP2	27	0.007451	4215608	0.243872
Up	CAPZA1	26	0.002869	626878	0.325191
Up	FKBP2	25	0.003414	683462	0.282255
Up	TWIST1	24	0.001372	488874	0.298643
Up	TIMP2	24	0.001662	741652	0.250924
Up	SPTLC1	24	0.008272	878220	0.320827
Up	DAPK1	24	0.003354	582304	0.3437
Up	STT3B	23	0.005888	688678	0.320805
Up	RBMS1	23	0.005223	803708	0.322772
Up	ANK3	21	0.004965	565126	0.321819
Up	TDG	21	0.00124	515588	0.326346
Up	CHMP5	20	0.00503	681262	0.320915
Up	MBD2	20	0.003168	3223460	0.265195
Up	TRIM25	20	0.002013	463938	0.326073
Up	TEAD3	20	0.001225	501618	0.323954
Up	SLC3A2	20	0.005187	569152	0.332435
Up	BMP1	19	0.00311	565522	0.259095
Up	RHOBTB3	19	0.002152	346198	0.322439
Up	CHD2	19	0.00121	998828	0.256894
Up	RYBP	19	0.002415	541940	0.322572
Up	GLRX	18	0.00459	583470	0.320959
Up	KMT2C	18	0.003676	553422	0.285019
Up	YTHDC1	18	0.003349	530960	0.33407
Up	GRB7	18	5.17E-04	151894	0.294958
Up	IWS1	17	0.004258	1715034	0.236798
Up	TRAF3IP2	17	0.001901	398464	0.324246
Up	CSF3R	17	0.001832	324530	0.329027
Up	AFF1	17	0.003482	1512904	0.247805
Up	INO80C	17	0.003498	441732	0.321245
Up	CASP4	17	0.002253	931980	0.263822
Up	YIPF6	17	0.003441	5013996	0.201367
Up	PCGF1	16	0.003259	409788	0.320827
Up	CXCR2	16	0.003899	2017434	0.253558
Up	ELF3	16	6.72E-04	203260	0.333452
Up	USP33	16	0.002932	430028	0.322927
Up	AGFG1	15	0.003227	435804	0.321443
Up	LNPEP	14	7.10E-04	211470	0.322483
Up	RORA	14	0.001493	299584	0.282187
Up	LARP1B	14	0.004683	546700	0.321708
Up	ACSL4	14	0.005208	423594	0.320388
Up	ESRRG	14	0.001317	573474	0.244687
Up	MXD1	13	6.21E-04	548286	0.273427
Up	GET4	13	0.001988	297146	0.321113
Up	NFE2	13	0.001758	361494	0.277475
Up	SCNN1B	13	0.001794	293352	0.321863
Up	TBX3	11	0.00107	660104	0.254826
Up	TAF1B	11	0.00167	339720	0.323106
Up	ADK	11	0.003862	318638	0.320257
Up	CLK3	11	0.001752	276054	0.272997
Up	PELI1	10	0.001025	246998	0.322195
Up	PHYHIPL	2	1.23E-05	3410	0.238801
Up	MAFF	2	8.27E-06	5370	0.260986
Up	TESK2	2	1.97E-05	2606	0.259425
Up	MTMR4	1	0	0	0.259138
Up	ZFAND6	1	0	0	0.230519
Up	ACP1	1	0	0	0.27858
Up	EFS	1	0	0	0.27858
Up	RAB11FIP5	1	0	0	0.220331
Up	CYP11A1	1	0	0	0.187328
Up	ADAM12	1	0	0	0.230951
Up	FBXO9	1	0	0	0.25419
Up	HSPB8	1	0	0	0.254674
Up	CCNDBP1	1	0	0	0.256234
Up	LTBP3	1	0	0	0.217731
Up	FNTA	1	0	0	0.246424
Up	ACOX3	1	0	0	0.24266
Up	PJA1	1	0	0	0.24372
Up	SERTAD4	1	0	0	0.247308
Up	EID2	1	0	0	0.259138
Up	AKTIP	1	0	0	0.254951
Up	SPTLC3	1	0	0	0.242911
Up	SGSM1	1	0	0	0.246424
Up	LRRC69	1	0	0	0.292236
Down	FYN	305	0.060673	15146282	0.386124
Down	ABL1	256	0.044808	14298188	0.385806
Down	SMAD3	209	0.042609	22810928	0.349754
Down	STAT3	202	0.037341	10226766	0.378422
Down	PRKCA	182	0.041662	11865040	0.377234
Down	ACTA2	161	0.02569	7462512	0.366351
Down	YWHAH	149	0.028069	10474112	0.350015
Down	ACTG2	131	0.011051	3779892	0.354141
Down	RPL14	127	0.022838	13966136	0.331377
Down	PDGFRB	123	0.013902	4225738	0.358778
Down	CCND1	119	0.017659	8841110	0.344483
Down	PPM1F	119	0.016265	6535610	0.32041
Down	CAV1	113	0.018938	4593828	0.368628
Down	YWHAQ	101	0.013637	4845932	0.355674
Down	FOXO1	96	0.015104	5652522	0.349128
Down	WNT3A	95	0.013629	4052094	0.299674
Down	RHOQ	92	0.016733	3678142	0.331471
Down	TUBB2A	91	0.012587	3886552	0.334213
Down	FGFR3	83	0.010949	3642512	0.326755
Down	RARA	81	0.013223	4465300	0.341869
Down	MYH11	72	0.01194	3062450	0.35249
Down	HIPK2	72	0.01135	3565432	0.339639
Down	HSPA12B	68	0.003037	744544	0.301991
Down	RPS6KA2	68	0.005549	2522498	0.317738
Down	TEK	66	0.004665	1653094	0.338168
Down	NCOA1	65	0.008395	3153458	0.31475
Down	VCL	63	0.009681	2780132	0.338217
Down	TUBB2B	62	0.00676	1694194	0.343977
Down	PPP2R2B	62	0.010479	4191976	0.328542
Down	TPM2	62	0.006776	1763750	0.334428
Down	SPTAN1	62	0.011399	2596256	0.334046
Down	MYL9	60	0.008098	2479906	0.335361
Down	DIMT1	58	0.008481	2242228	0.327326
Down	TCF4	54	0.009445	2760706	0.330629
Down	MRPL34	52	0.006656	17810170	0.236106
Down	CCND3	51	0.003239	1601134	0.295927
Down	LMNA	47	0.00911	2401846	0.338682
Down	PKN1	46	0.003592	1349794	0.299559
Down	CD44	46	0.010187	2270300	0.349493
Down	DCN	45	0.007594	1930616	0.322283
Down	ADCY3	45	0.008618	1694556	0.33133
Down	TGFB1I1	45	0.003137	931752	0.325349
Down	EPHA3	44	0.002379	661600	0.318884
Down	FZD4	43	0.005096	1738748	0.327006
Down	RND3	42	0.006152	1123788	0.333832
Down	ADCY4	41	0.001819	672196	0.287785
Down	ETS2	41	0.004246	1834106	0.329559
Down	RAP2A	40	0.007351	1433240	0.326983
Down	CCND2	40	0.002601	1155154	0.334046
Down	AXIN2	39	0.00394	1203884	0.333618
Down	OPTN	37	0.00695	1707428	0.328289
Down	UBE2L6	37	0.005601	1462120	0.327189
Down	PIP5K1C	37	0.002815	1263578	0.334714
Down	MAP1B	34	0.007029	1488054	0.325371
Down	AXL	34	0.002374	729250	0.345881
Down	IRAK3	33	8.12E-04	865022	0.277229
Down	FLNA	33	0.004649	1087030	0.310146
Down	COL1A1	32	0.008312	1395294	0.32942
Down	DLL1	31	0.006383	1006300	0.324583
Down	JAG1	29	0.005281	874336	0.325033
Down	PDE9A	29	0.003164	644058	0.281577
Down	AGTR1	28	0.004101	926250	0.304088
Down	TNNT3	27	1.64E-04	89708	0.268585
Down	MYO10	27	0.002183	551478	0.325892
Down	GPC4	26	0.001618	1207760	0.258652
Down	IGFBP3	26	0.004163	894182	0.300288
Down	ABCE1	25	0.007191	831802	0.321973
Down	TIMP1	23	0.006574	950444	0.322905
Down	VTN	23	0.005502	970948	0.284138
Down	ARAP3	23	8.48E-04	298776	0.326642
Down	COL1A2	23	0.001462	373484	0.28554
Down	B2M	23	0.00758	619198	0.322505
Down	TBCB	22	0.002726	428028	0.322705
Down	VASN	22	0.003962	582444	0.343574
Down	LGALS1	21	0.004371	578082	0.333903
Down	CD81	21	0.005039	710796	0.323708
Down	VCAM1	21	0.003333	2717522	0.2699
Down	THY1	21	0.003795	675432	0.288956
Down	PHGDH	21	0.004117	689964	0.323999
Down	ITPR3	21	0.003472	727920	0.327326
Down	TGFB3	20	0.002631	725004	0.278316
Down	BIN1	20	0.002083	351704	0.291835
Down	LPAR1	20	0.001469	1296676	0.242258
Down	MSX1	20	0.003633	648486	0.289134
Down	SLIT2	20	0.001084	281232	0.291273
Down	SHMT2	19	0.005637	547666	0.32215
Down	COL4A1	19	0.001742	983934	0.257813
Down	RAB3IL1	19	0.002314	1857444	0.231054
Down	SPARC	17	0.002023	197820	0.275923
Down	VCAN	17	0.003487	561304	0.267405
Down	LDB2	17	0.003396	1291388	0.254812
Down	DPYSL2	17	0.003321	506826	0.325869
Down	MAF	17	0.001088	288082	0.289456
Down	PARVA	16	0.003041	421786	0.323731
Down	TNFRSF1B	16	0.001419	325188	0.32382
Down	FBLN2	16	0.001835	589690	0.249987
Down	EEF2K	15	0.001411	364288	0.323083
Down	TEAD2	15	7.99E-04	352390	0.324066
Down	TAGLN	15	0.001241	205764	0.280381
Down	FASN	15	0.001757	367956	0.322084
Down	CD36	15	0.002116	386412	0.294661
Down	NDN	15	0.003831	473694	0.322239
Down	ADAM15	15	0.001982	294010	0.331283
Down	ARHGEF2	15	9.41E-04	262426	0.330396
Down	PEMT	14	0.005116	629532	0.293664
Down	VWF	14	0.001828	345586	0.275097
Down	OXTR	14	0.001359	1221820	0.23325
Down	FRYL	14	0.001994	332012	0.321598
Down	AGRN	14	0.001733	263834	0.326482
Down	LFNG	13	0.001654	331212	0.264357
Down	ARHGEF6	13	0.001032	179796	0.326573
Down	TSC22D3	13	1.34E-04	119696	0.32391
Down	IMPDH1	13	0.002712	390200	0.321554
Down	NME4	12	0.003215	901430	0.228485
Down	EHD2	12	0.003128	309602	0.320717
Down	RGL1	12	5.53E-04	670384	0.247308
Down	COL18A1	12	0.001098	309928	0.266008
Down	ADGRA2	12	8.13E-04	196338	0.301583
Down	FMOD	11	2.68E-04	98450	0.300385
Down	SLIT3	10	6.95E-05	22762	0.24207
Down	COL5A1	3	4.31E-05	14682	0.244636
Down	TGFBI	3	8.62E-06	14986	0.248607
Down	RASIP1	2	1.64E-06	1660	0.251732
Down	CAV2	2	0	0	0.285836
Down	PCOLCE	2	3.25E-05	6584	0.246761
Down	COL4A2	2	0	0	0.208517
Down	SVIL	2	0	0	0.262801
Down	SMTN	2	7.00E-06	2536	0.261073
Down	COL4A5	2	7.45E-06	6672	0.244776
Down	FSCN1	2	2.41E-06	2090	0.268585
Down	ADAMTS1	2	5.19E-05	3268	0.250857
Down	CTSC	1	0	0	0.244559
Down	FEZ1	1	0	0	0.21526
Down	MALL	1	0	0	0.269357
Down	LYL1	1	0	0	0.248489
Down	SEMA3A	1	0	0	0.27858
Down	ENPP2	1	0	0	0.195022
Down	BST2	1	0	0	0.24757
Down	F3	1	0	0	0.236738
Down	COL16A1	1	0	0	0.204978
Down	ANGPTL2	1	0	0	0.252724
Down	NCALD	1	0	0	0.250508
Down	RASL12	1	0	0	0.228262
Down	PPP1R14A	1	0	0	0.273923
Down	FCGRT	1	0	0	0.243872
Down	CNN2	1	0	0	0.268139
Down	EHBP1	1	0	0	0.242848
Down	COL3A1	1	0	0	0.216264
Down	DDIT4	1	0	0	0.25408
Down	ITM2C	1	0	0	0.249441
Down	GAS6	1	0	0	0.257007
Down	FERMT2	1	0	0	0.252751
Down	RAPGEF5	1	0	0	0.246424
Down	APCDD1	1	0	0	0.230588
Down	MMP23B	1	0	0	0.200599
Down	PTPRD	1	0	0	0.274549
Down	RSPO3	1	0	0	0.249441
Down	COL6A1	1	0	0	0.243745
Down	DDR2	1	0	0	0.247805
Down	NEK6	1	0	0	0.244776
Down	COL5A2	1	0	0	0.205788
Down	TSPAN4	1	0	0	0.244559
Down	IFITM3	1	0	0	0.27858
Down	HES4	1	0	0	0.261875
Down	FAT1	1	0	0	0.242521

### MiRNA–hub gene regulatory network construction

**Figure 5:**
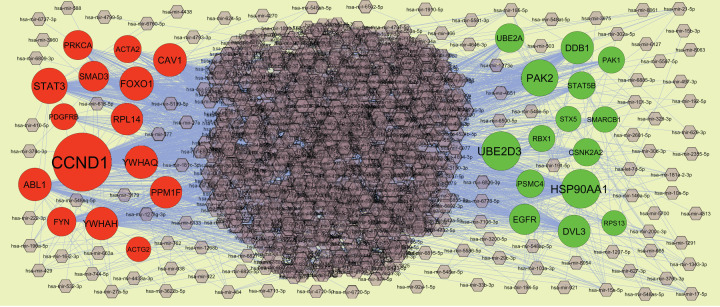
MiRNA–hub gene regulatory network The light purple color diamond nodes represent the key miRNAs; up-regulated genes are marked in green; down-regulated genes are marked in red.

miRNet database was applied to screen the targeted miRNAs of the hub genes. Cytoscape software was used to construct the miRNA–hub gene network. As illustrated in Figure 5, the interaction network consists of 307 hub genes and 2280 miRNAs. The hub genes and miRNAs in the network were ranked by their degree of connectivity using Network Analyzer and are listed in Table 6. Based on the expression trend of hub genes in GDM, we found that UBE2D3 was the predicted target of hsa-mir-6127, HSP90AA1 was the predicted target of hsa-let-7d-5p, PAK2 was the predicted target of hsa-mir-8063, DDB1 was the predicted target of hsa-mir-329-3p, DVL3 was the predicted target of hsa-mir-1207-5p, FYN was the predicted target of hsa-mir-4651, ABL1 was the predicted target of hsa-mir-410-5p, SMAD3 was the predicted target of hsa-mir-222-3p, STAT3 was the predicted target of hsa-mir-29c-3p and PRKCA was the predicted target of hsa-mir-663a.

**Table 6:** miRNA–hub gene and TF–hub gene interactions

Regulation	Hub Genes	Degree	MicroRNA	Regulation	Hub Genes	Degree	TF
Up	UBE2D3	189	hsa-mir-6127	Up	HSP90AA1	45	E2F1
Up	HSP90AA1	188	hsa-let-7d-5p	Up	UBE2D3	43	HCFC1
Up	PAK2	158	hsa-mir-8063	Up	EGFR	39	SRY
Up	DDB1	108	hsa-mir-329-3p	Up	PSMC4	34	ZFX
Up	DVL3	104	hsa-mir-1207-5p	Up	DDB1	32	RUNX1
Up	EGFR	83	hsa-mir-181a-2-3p	Up	STAT5B	28	ESR1
Up	UBE2A	60	hsa-mir-5700	Up	PAK2	27	REST
Up	PSMC4	47	hsa-mir-665	Up	RBX1	27	YY1
Up	CSNK2A2	45	hsa-mir-30d-3p	Up	STX5	24	SREBF2
Up	STAT5B	42	hsa-mir-1343-3p	Up	CSNK2A2	21	SIN3A
Up	PAK1	31	hsa-mir-629-3p	Up	RPS13	19	ASH2L
Up	RBX1	29	hsa-mir-4513	Up	SMARCB1	18	EGR1
Up	SMARCB1	21	hsa-mir-192-5p	Up	DVL3	17	TTF2
Up	STX5	15	hsa-mir-146a-5p	Up	PAK1	16	TP63
Up	RPS13	10	hsa-mir-15b-3p	Up	UBE2A	11	TRIM28
Down	CCND1	396	hsa-mir-4651	Down	STAT3	67	SPI1
Down	STAT3	148	hsa-mir-410-5p	Down	CCND1	56	MYBL2
Down	FOXO1	124	hsa-mir-222-3p	Down	SMAD3	53	SUZ12
Down	CAV1	115	hsa-mir-29c-3p	Down	FOXO1	44	TBX3
Down	ABL1	112	hsa-mir-663a	Down	PRKCA	43	YAP1
Down	YWHAH	112	hsa-mir-577	Down	ABL1	40	TEAD4
Down	YWHAQ	111	hsa-mir-16-2-3p	Down	FYN	39	CEBPD
Down	RPL14	99	hsa-mir-3960	Down	YWHAQ	38	SOX9
Down	PPM1F	99	hsa-mir-638	Down	CAV1	36	BMI1
Down	SMAD3	78	hsa-mir-744-5p	Down	YWHAH	31	RCOR3
Down	PRKCA	67	hsa-mir-6727-3p	Down	RPL14	29	LMO2
Down	FYN	61	hsa-mir-429	Down	PDGFRB	28	TAL1
Down	ACTA2	42	hsa-mir-376c-3p	Down	PPM1F	18	FOXP1
Down	PDGFRB	28	hsa-mir-101-3p	Down	ACTA2	14	E2F4
Down	ACTG2	10	hsa-mir-103a-3p	Down	ACTG2	8	CUX1

### TF–hub gene regulatory network construction

**Figure 6:**
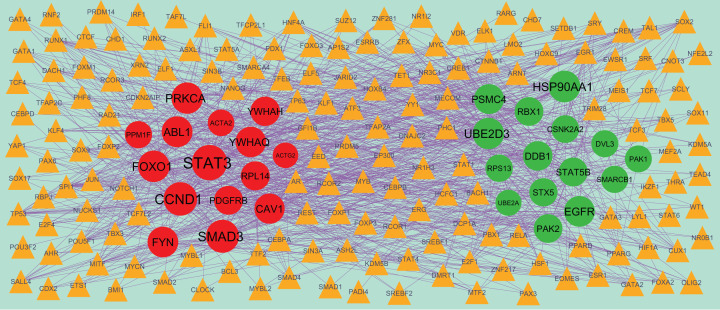
TF–hub gene regulatory network The yellow color triangle nodes represent the key TFs; up-regulated genes are marked in green; down-regulated genes are marked in red.

NetworkAnalyst database was applied to screen the targeted TFs of the hub genes. Cytoscape software was used to construct the TF–hub gene network. As illustrated in Figure 6, the interaction network consists of 306 hub genes and 195 TFs. The hub genes and TFs in the network were ranked by their degree of connectivity using Network Analyzer and are listed in Table 6. Based on the expression trend of hub genes in GDM, we found that HSP90AA1 was the predicted target of E2F1, UBE2D3 was the predicted target of HCFC1, EGFR was the predicted target of SRY, PSMC4 was the predicted target of ZFX, DDB1 was the predicted target of RUNX1, STAT3 was the predicted target of SPI1, CCND1 was the predicted target of MYBL2, SMAD3 was the predicted target of SUZ12, FOXO1 was the predicted target of TBX3 and PRKCA was the predicted target of YAP1.

###  ROC curve analysis

**Figure 7:**
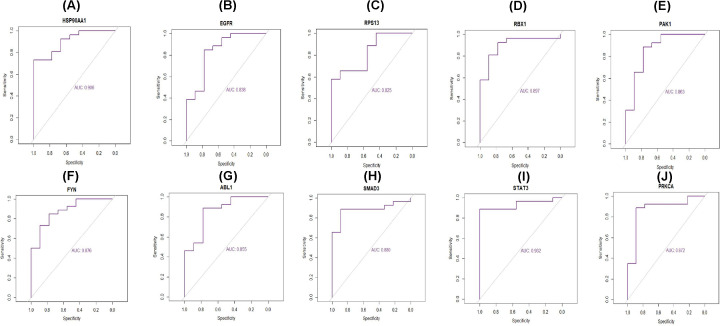
ROC curve validated the sensitivity, specificity of hub genes as a predictive biomarker for GDM prognosis (**A**) HSP90AA1, (**B**) EGFR, (**C**) RPS13, (**D**) RBX1, (**E**) PAK1, (**F**) FYN, (**G**) ABL1, (**H**) SMAD3, (**I**) STAT3, (**J**) PRKCA.

ROC curve analysis was implemented to evaluate the capacity of hub genes to distinguish GDM and non-GDM in E-MTAB-6418, HSP90AA1, EGFR, RPS13, RBX1, PAK1, FYN, ABL1, SMAD3, STAT3 and PRKCA, exhibiting better diagnostic efficiency for GDM and non-GDM, and the combined diagnosis of these ten hub genes was more effective. The AUC index for the ten hub gene scores were 0.906, 0.838, 0.825, 0.897, 0.863, 0.876, 0.855, 0.880, 0.932 and 0.872, and are shown in Figure 7.

### RT-PCR analysis

**Figure 8:**
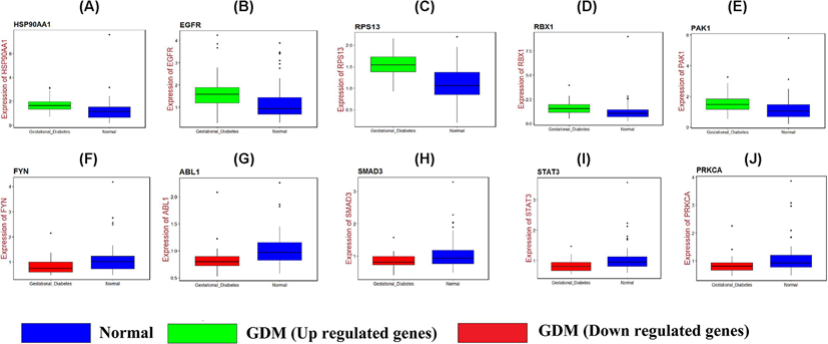
Validation of hub genes by RT- PCR (**A**) HSP90AA1, (**B**) EGFR, (**C**) RPS13, (**D**) RBX1, (**E**) PAK1, (**F**) FYN, (**G**) ABL1, (**H**) SMAD3, (**I**) STAT3, (**J**) PRKCA.

To further verify the expression level of hub genes in GDM, RT-PCR was performed to calculate the mRNA levels of the ten hub genes identified in the present study (*HSP90AA1, EGFR, RPS13, RBX1, PAK1, FYN, ABL1, SMAD3, STAT3* and *PRKCA*) in GDM. As illustrated in Figure 8, the expressions of *HSP90AA1, EGFR, RPS13, RBX1, PAK1* were significantly up-regulated in GDM samples compared with normal, while *FYN, ABL1, SMAD3, STAT3 *and *PRKCA* were significantly down-regulated in GDM samples compared with normal. The present RT-PCR results were in-line with the aforementioned bioinformatics analysis, suggesting that these hub genes might be linked to the molecular mechanism underlying GDM.

### Molecular docking experiments

**Figure 9:**
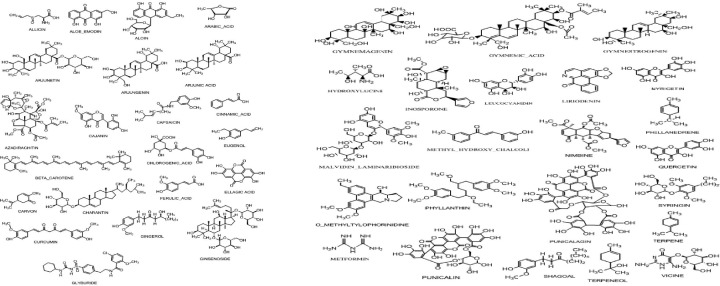
Chemical structures of phytoconstituents

**Figure 10:**
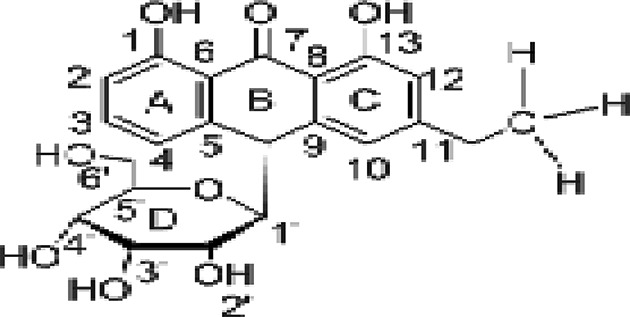
Structure of ALO

**Figure 11:**
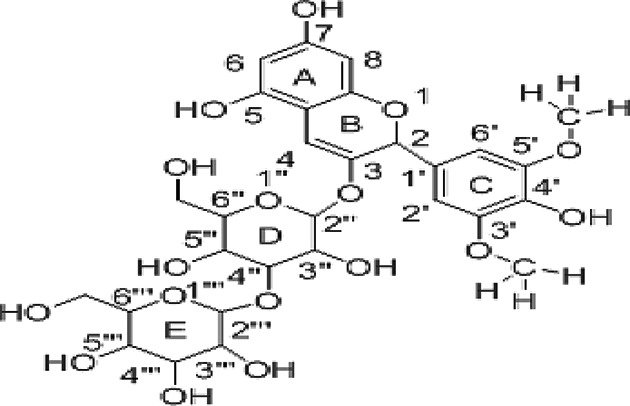
Structure of MAL

**Figure 12:**
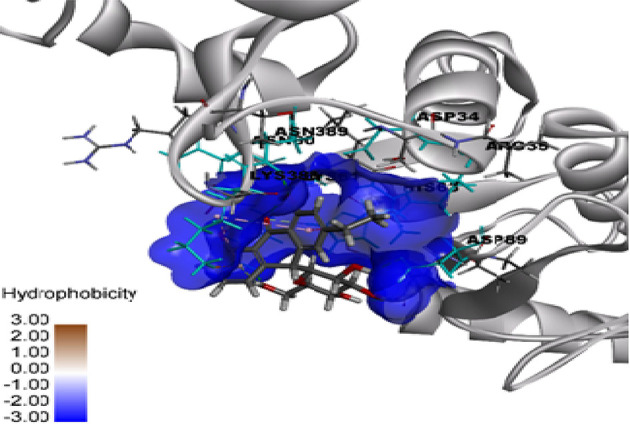
3D binding of ALO with 3FN1

**Figure 13:**
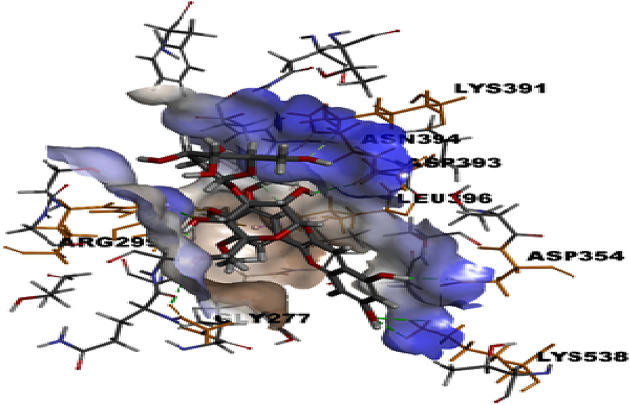
3D binding of MAL with 3Q4Z

**Figure 14:**
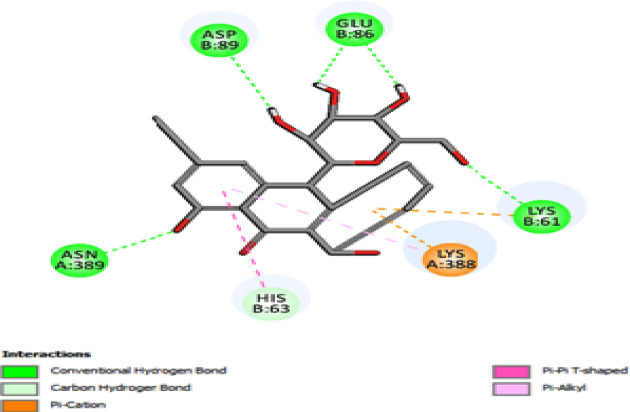
2D binding of ALO with 3FN1

**Figure 15:**
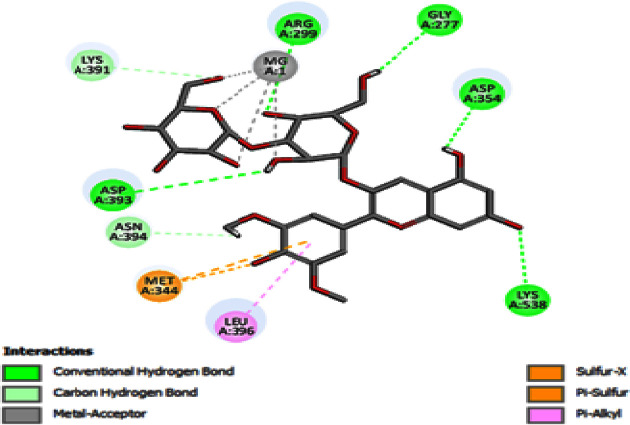
2D binding of MAL with 3Q4Z

In the recent findings, the docking study was performed using Biovia Discovery Studio perpetual software to analyze the binding pattern of the natural plant products such as herbs have the ability to lower blood glucose levels and ameliorate diabetes with decreased adverse side effects. The natural well-known phytoconstituents which decreases the blood sugar level are Malvidin 3-laminaribioside (MLR), Ferulic acid (FRA), Inosporone (INO), Allicin (ALL), Liriodenin (LIR), Azadirachitin (AZA), Sulforaphane, Cajanin (CAJ), Carvone (CAR), Capsaicin (CAP), Terpineol (TER), Phellandrene (PHE), Terpene (TPN), Ellagic acid (ELA), Leucodelphinidin, O-methyltylophorinidine (OMT), Gymnemic acid, β-Carotene (BCR), Leucocyanidin (LEC), Syringin (SYR), Ginsenoside (GNS), Phyllanthin (PHY), Punicalagin (PUC), Punicalin (PUN), Arjunic acid (AJA), Arjunetin (ARJ), Arabic acid (ARA), Arjungenin (ARG), Gingerol (GIN), Shogaol, Aloe emodin (ALE), Arabic acid (ARA), Aloin (ALO), Charantin (CHR), Cinnamic acid (CIN), Curcumin (CUR), Euginol (EUG), Gymnemagenin (GMG), Gymnestrogenin (GYM), Hydroxylucin (HYD), Methoxy hydroxyl chalcoli (MHC), Myricetin (MYR), Nimbine (NIM), Quercetin (QUE), Vicine (VIC) and Shagoal (SHA) are shown in Figure 9. The molecules were constructed based on the natural plant products containing these chemical constituents which play vital role in reducing type 2 diabetes mellitus. The traditional plant products are used in conjunction with allopathic drug to reduce the dose of the allopathic drugs and/or to increase the efficacy of allopathic drugs. Some common and most prominent antidiabetic plants and active principles were selected from their phytochemicals for docking studies in the present research to identify the active natural molecule to avoid the use of allopathic drugs in gestational diabetes and the blood sugar level is controlled by altering the diet. For docking experiments well-known and most commonly used two allopathic drugs such as Glyburide (GLY), Metformin (MET) in gestational diabetes are used as standard and to compare the binding interaction of natural phytoconstituents with allopathic drugs. A total of common 44 in that 42 natural active constituents, few from each of flavonoids, saponins, tannins and glycosides etc., present in plant extracts responsible for antidiabetic function and 2 allopathic drugs were chosen for docking studies on overexpressed proteins and the structures are depicted in Figure 1, respectively. The one protein from each overexpressed gene in gestational type 2 diabetes mellitus such as EGFR, HSP90AA1, PAK1 and RBX1 and their X-ray crystallographic structure and co-crystallized PDB code and their PDB codes 4UV7, 5NJX, 3Q4Z and 3FNI, respectively were constructed for docking. The docking on natural active constituents was conducted to classify the potential molecule and their binding affinity to proteins. A higher number of negative number -CDOCKER energy and binding energy indicates a stronger binding interactions with proteins, few constituents obtained with a greater -CDOCKER energy and binding energy respectively with particular proteins. Docking experiments were carried out on a total of 42 constituents from plant products, few constituents obtained excellent -CDOCKER energy and binding energy. Out of 44 molecules, few of the molecules obtained -CDOCKER interaction energy of more than 40 and majority with more than 30 and less than 40, few molecules obtained optimum -CDOCKER interaction energy of less than 30, respectively. the molecules with -CDOCKER interaction energy of 40 and above are said to have good interaction with proteins and stable. The natural constituents of the molecules GLY, GNS, GYM, MLR, PUC and ALO, GLY, MLRand ALE, ALO, BCR, CAP, CHR, ELA, LUR, GIN, GLY, GMG, GNS, GYM, LEC, LIR, MLR, MYR, NIM, OMP, PHY, PUC, PUN, QUE, SHE,VI C obtained a -CDOCKER interaction energy of more than 40 with protein of PDB codes 5NJX and 3FNI and 3Q4Z, respectively. The natural constituents obtained -CDOCKER interaction energy of less than 40 and more than 30 are ALO, ARJ, BCR, CHR, CUR, PHY, PUN and BCR, CAJ, CAP, CUR, GIN, LEC, MYR, OMP, QUE, VIC and AJA, ARA, ARG, CAJ, FRA, HYD, MHC and GNS, PHY, PUC, PUN with 5NJX and 3FNI and 3Q4Z and 4UV7. The constituents obtained less than 30 and more than 20 are AJA, ALE, ARG, CAJ, CAP, GIN, GMG, GYM, HYD, LEC, MHC, MLR, MYR, NIM, OMP, QUE, VIC and AJA, ALE, ALL, ARG, AJA,CHR, CIN, EUG, FRA, GMG, GNS, GYM, LIR, MHC, NIM, PUC and ALL, CIN, EUG, MET, TER and ALA, ALE, ALO, ARJ, BCR, CAJ, CHR, ELA, FRA, GIN, GMG, LEC, MLR, MYR, OMP, QUE, SHA with 5NJX and 3FNI and 3Q4Z and 4UV7. Following the molecules obtained less than 20 -CDOCKER interaction energy are ALL, ARA, CAR, CHR, CIN, EUG, FRA, LIR, MET, PHE, TER, TPN and ARJ, ARA, CAR, HYD, MET, PHE, TPN and CAR, PHE, TPN and AJA, ALL, ARG, CAR, CIN, EUG, GYM, HYD, LIR, MET, MHC, NIM, PHE, TER, TPN, VIC with protein 5NJX and 3FNI and 3Q4Z and 4UV7, respectively, the binding energy, -CDOCKER energy and -CDOCKER interaction energy are depicted in Table 7. The two molecules such as ALO and MAL (Figures 10 and 11), their interaction with amino acids of proteins with 3D structures for 3FN1 (Figure 12) and 3Q4Z (Figure 13), while 2D structures for 3FN1 (Figure 14) and 3Q4Z (Figure 15).

**Table 7:** Docking results of designed molecules on overexpressed proteins

Sl. No/Code	EGFR			HSP90AA1			PAK1			RBX1		
	PDB: 4UV7			PDB:5NJX			PDB: 3Q4Z			PDB: 3FN1		
	Binding Energy	−Cdocker Energy	− Cdocker Interaction Energy	Binding Energy	−Cdocker Energy	− Cdocker Interaction Energy	Binding Energy	−Cdocker Energy	− Cdocker Interaction Energy	Binding Energy	−Cdocker Energy	− Cdocker Interaction Energy
AJA	−56.02	−10.57	18.17	−77.05	−6.93	21.66	−196.65	13.03	43.86	−113.94	5.61	37.90
ALE	−22.04	−17.45	10.55	−16.93	−16.44	12.00	−58.34	−2.81	24.49	−24.28	−9.71	18.84
ALO	−6.22	−25.19	8.57	−2.69	−24.11	9.65	−4.41	−19.09	14.60	−11.36	−18.02	15.96
ARA	−50.94	−28.62	19.50	−57.53	−18.31	26.43	−159.25	8.76	57.02	−83.85	−16.52	33.54
ARJ	−43.98	17.23	28.38	−39.56	18.14	25.73	−119.99	33.00	43.82	−39.87	26.58	31.46
ARG	−49.90	20.57	23.74	−61.58	22.29	25.37	−151.03	40.87	47.08	−7.21	−116.32	22.65
ANA	−65.59	−68.87	32.04	−89.71	−66.11	51.76	−245.05	−79.05	83.86	−61.60	21.05	41.01
ALL	−118.34	−47.02	34.21	−168.97	−50.42	39.91	−139.90	−23.49	62.54	−18.62	−15.64	14.90
CAR	−86.83	19.53	35.26	−45.09	19.64	36.45	−131.24	37.17	58.34	−48.59	−55.20	21.22
CAJ	−6.67	−21.05	9.42	−6.26	−20.34	10.30	−9.30	−16.06	13.09	−47.54	28.64	30.02
CAP	−35.79	−58.29	19.06	−42.81	−50.54	28.01	−172.32	−28.95	57.88	−6.63	−25.87	30.85
CAV	−65.69	22.19	25.24	−53.63	25.00	27.33	−136.49	43.84	47.47	−46.65	19.83	28.55
CHA	−4.05	−30.55	21.32	−35.08	−30.24	21.35	−107.73	−7.42	44.11	−27.07	13.28	14.79
CHL	−14.17	8.84	17.63	−49.92	15.28	24.60	−81.41	27.24	34.82	−80.66	−26.20	43.47
CIN	−50.02	12.60	14.67	−27.06	11.55	13.70	−92.97	18.99	21.39	−29.49	−17.74	24.17
CUR	−61.32	−38.90	27.73	−64.69	−22.71	40.68	−276.18	16.00	87.65	−61.40	18.48	32.45
ELL	−40.77	−22.62	18.69	−34.20	−24.49	16.72	−137.67	1.59	41.48	−73.55	20.96	35.11
EUG	−71.25	14.65	28.33	−71.08	13.46	27.19	−149.04	30.21	46.83	−40.14	−27.96	25.52
FER	−84.22	11.92	24.50	−38.93	14.41	25.46	−157.42	32.92	48.59	−74.77	−97.25	24.35
GIN	−26.63	−36.38	14.98	−51.74	−30.08	21.65	−135.45	−15.24	39.95	−47.21	14.33	17.68
GNS	−36.67	10.71	17.53	−41.60	12.11	20.48	−132.64	26.84	30.74	−84.78	−82.04	29.12
GYM	−41.53	−84.64	23.43	−52.36	−79.44	27.80	−110.88	−55.77	55.03	−78.34	−164.85	26.81
GYA	−75.68	−162.34	32.37	−86.27	−145.33	40.68	−160.78	−122.08	67.89	−75.89	20.89	41.44
GMT	−4.26	−99.21	17.86	−72.64	−91.68	25.64	−109.57	−79.81	40.10	−47.57	30.04	32.56
HYD	−68.08	19.53	34.12	−125.59	23.82	41.37	−158.25	41.54	59.92	−20.94	20.67	23.23
INO	−50.91	24.87	26.12	−43.89	24.42	27.14	−87.39	39.52	44.59	−28.20	15.41	23.15
LEU	−38.73	18.54	21.60	−26.25	15.39	18.10	−122.22	27.38	32.75	−48.37	26.66	39.62
LEP	−16.17	7.13	15.51	−21.35	8.09	16.21	−62.06	19.28	25.76	−31.27	18.48	20.64
LIR	−53.70	9.95	22.56	−84.07	18.36	27.30	−142.76	31.85	41.89	−29.11	−73.02	27.80
MAL	−58.66	21.70	30.45	−34.92	23.71	32.09	−138.59	36.48	50.57	−24.50	−15.13	18.76
MHC	−28.62	13.37	15.74	−32.04	13.89	16.17	−33.87	21.61	22.73	−40.19	−77.90	31.36
MYR	−22.60	−74.73	29.54	−51.69	−68.66	31.40	−94.78	−45.63	63.61	−48.31	20.69	36.63
NIM	−17.29	−20.00	11.23	−22.94	−18.68	12.75	−39.38	−11.59	19.23	−73.77	21.14	30.96
MPO	−26.31	−85.18	28.60	−27.95	−81.28	30.13	−62.20	−67.23	48.83	−49.69	−107.43	17.79
PHE	−52.17	12.68	21.80	−59.98	17.35	28.95	−140.57	34.70	49.40	−51.52	−95.45	20.50
PUN	−23.27	−121.88	26.67	−62.56	19.02	28.47	−100.15	27.33	36.00	−6.35	3.83	17.39
PUC	−22.94	−98.54	23.93	−110.98	−114.66	31.90	−147.91	−67.17	73.20	−25.35	−112.29	24.27
QUE	−25.04	−95.41	19.39	−29.38	−88.29	31.16	−182.08	−73.82	62.28	−119.62	14.22	41.65
SHA	−62.78	4.01	17.60	−97.71	−87.07	28.88	−133.51	−71.22	39.42	−38.98	22.34	28.18
SYR	−42.02	−112.72	18.53	−57.33	0.94	14.08	−111.64	16.61	30.08	−28.67	15.86	22.42
TER	−48.17	3.01	26.96	−45.14	−109.25	24.75	−88.49	−99.19	31.30	−55.95	20.86	33.04
TPN	−48.96	17.76	22.80	−58.53	5.58	30.24	−151.71	24.04	55.75	−38.43	15.22	28.09
VIC	−49.26	11.32	17.70	−60.62	17.96	23.33	−150.32	32.32	43.25	−15.63	21.72	55.13
GLY	−49.25	15.93	28.98	−46.36	9.39	14.16	−83.17	23.57	29.57	−25.71	18.79	28.22
MET	−35.21	18.35	32.89	−25.62	34.77	17.74	−196.65	13.03	43.86	−61.04	27.56	32.39

## Discussion

GDM is a metabolic disorder that can be caused by various factors, including genetics and the endocrine system. It is essential to understand the molecular mechanisms underlying GDM in order to find and advance more valid diagnostic and therapeutic strategies. Gene chip technology is generally used to reveal the expression levels of numerous genes within the human genome and might help in the recognition of target genes of interest for diagnosing or treating GDM.

In our study, a total of 869 DEGs were screened, including 439 up-regulated genes and 430 down-regulated genes. The CGB5 was associated with pregnancy success and might be a possible genetic marker for pregnancy success [30]. Studies have reported that corticotropin releasing hormone (CRH) [31], PSG1 [32] and CYP19A1 [33] are directly related to the development preeclampsia. CD248 has become an attractive target for hypertension [34], but this gene might be novel target for GDM. A previous study showed that COL1A1 [35] was expressed in type 2 diabetes mellitus, but this gene might be novel target for GDM. In a recent study, ABI3BP could facilitate the progression of cardiovascular diseases [36], but this gene might be novel target for GDM. In previous studies, there was a large amount of evidence that MFAP4 [37] directly or indirectly affects the occurrence and development of type 1 diabetes mellitus, but this gene might be novel target for GDM.

Potential pathways were obtained after GO and pathway enrichment analysis. Evidence suggests that CEBPB (CCAAT enhancer binding protein β) [38], ACSL4 [39], MBD2 [40], ULK1 [41], NUCB2 [42], TWIST1 [43], HOOK2 [44], CLDN7 [45], TBK1 [46], YIPF6 [47], TFRC (transferrin receptor) [48], ENPP2 [49], SLIT2 [50], MFGE8 [51], FAT1 [52], GPC4 [53], COL6A3 [54], EGFL6 [55], AOC3 [56], CCN2 [57], LYVE1 [58], RARA (retinoic acid receptor α) [59], COL18A1 [60], THY1 [61], CD36 [62], PEMT (phosphatidylethanolamine N-methyltransferase) [63], AIF1L [64], OXTR (oxytocin receptor) [65], LMNA (lamin A/C) [66], CXCL14 [67], DKK3 [68], ANGPTL2 [69] and CMTM7 [70] might be regarded as genetic factors in humans due to their involvement in obesity, but these genes might be novel target for GDM. Expression sites of AHR (aryl hydrocarbon receptor) [71], STS (steroid sulfatase) [72], PLAC1 [73], CYP11A1 [74], PSG11 [75], STAT5B [76], TLR3 [77], FOLR1 [78], HSPB1 [79], HSP90AA1 [80], ANXA4 [81], ATF3 [82], DAPK1 [83], ENTPD1 [84], ABL1 [85], VSIG4 [86], CD99 [87], VWF (von Willebrand factor) [88], PODXL (podocalyxin like) [89], PDPN (podoplanin) [90], RND3 [91], VCAN (versican) [92], AXL (AXL receptor tyrosine kinase) [93], PIEZO1 [94], GAS6 [93], LAMA4 [95], CAV1 [96], DLL1 [97], CD44 [98], CD81 [99], SMAD3 [100], NES (nestin) [101], DCN (decorin) [102], AGTR1 [103], SLIT3 [104], B2M [105], STAT3 [106], STC1 [107], ADAMTS1 [108], HSD11B2 [109] and HSD3B1 [110] in preeclampsia were specific, but these genes might be novel target for GDM. Increasing evidence indicates that the development of type 2 diabetes mellitus, due to the dysregulation of genes, such as CSNK2A2 [111], NFE2 [112], CAMK2G [113], RASGRP1 [114], S100P [115], SRR (serine racemase) [116], DHPS (deoxyhypusine synthase) [117], DYRK1A [118], JAG1 [119], COL3A1 [120], VTN (vitronectin) [121], WNT3A [122], ACTA2 [123], SEMA3A [124], RARRES2 [125], CAV2 [126] and SPRED1 [127], but these genes might be novel target for GDM. In a previous report, Santiago et al. [128], Auburger et al. [129], Qu et al. [130], Śnit et al. [131] and Hjortebjerg et al. [132] reported that SLC22A5, SH2B3, ITPR3, CALD1 and IGFBP4 served important roles in type 1 diabetes mellitus, but these genes might be novel target for GDM. Krishnan et al. [133], Hu et al. [134], Martins et al. [135], Prieto-Sánchez et al. [136], Sugulle et al. [137], Zhao et al. [138], Siddiqui et al. [139], Han et al. [140], Lappas et al. [141], Wang et al. [142], Artunc-Ulkumen et al. [143], Blois et al. [144], Vacínová et al. [145] and Vilmi-Kerälä et al. [146] demonstrated that the expression of CREBRF (CREB3 regulatory factor), STRA6, EGFR, MFSD2A, GDF15, PAK1, VCAM1, IGFBP2, IGFBP7, PRKCA (protein kinase C α), ADAMTS9, LGALS1, BIN1 are susceptibility for GDM, but further analysis of the function remains to be seen. Aquila et al. [147], Chen et al. [148], Xie et al. [149], Zhang et al. [150], Aspit et al. [151], Akadam-Teker et al. [152], Jiang et al. [153], Cetinkaya et al. [154], Grond-Ginsbach et al. [155], Dong et al. [156], Chardon et al. [157], Chen et al. [158], Yamada et al. [159], Hu et al. [160], Bobik and Kalinina [161], Schwanekamp et al. [162], Liu et al. [163], Schroer et al. [164], Raza et al. [165], Yang et al. [166], Azuaje et al. [167], Durbin et al. [168], Chowdhury et al. [169], Wang et al. [170], Li et al. [171], Lv et al. [172], Bertoli-Avella et al. [173], Grossman et al. [174], Andenæs et al. [175] and Chen et al. [176] demonstrated that expression of HES1, SPIN1, TBX3, EVA1A, CAP2, BMP1, HSPB8, RDX (radixin), COL5A1, LIMS2, PARVA (parvin α), EGFLAM (EGF like, fibronectin type III and laminin G domains), NEXN (nexilin F-actin binding protein), TNFRSF14, TGFBI (transforming growth factor β induced), HAVCR2, CDH11, COL4A1, COL4A2, COL5A2, SHROOM3, HYAL2, PDLIM3, ETS2, PLSCR4, TGFB3, COL6A2 and LTBP2 have previously been detected in cardiovascular diseases as well, but these genes might be novel target for GDM. Flamant et al. [177], Wan et al. [178], Zhang et al. [179], Vallvé et al. [180], Heximer and Husain [181], Selvarajah et al. [182], Jain et al. [183], Sun et al. [184], Satomi-Kobayashi et al. [185], Jiang et al. [186], Waghulde et al. [187] and Dahal et al. [188] reported that DDR1, CAST (calpastatin), KYNU (kynureninase), FBLN2, SPON1, VEGFC (vascular endothelial growth factor C), FLNA (filamin A), SNAI2, MYADM (myeloid associated differentiation marker), NECTIN2 and SMTN (smoothelin), GPER1, PDGFRB (platelet-derived growth factor receptor β) are important genetic factors related to hypertension, but these genes might be novel target for GDM. This investigation demonstrated that the pathogenesis of GDM has genetic heterogeneity. These candidate genes might play a pathogenic role through different signaling pathways, and different gene alteration might lead to different system damage in GDM. The genetic pathogenesis of GDM will become a more research hotspot again.

From the PPI network and modules diagram, it can be observed that hub genes were the key nodes of the PPI network and modules, with the highest node degree, betweenness, stress and closeness value. RPS13, RBX1, FYN, UBE2A, TUBB2A and TBCB were the novel biomarkers for the progression of GDM.

From the miRNA–hub gene network construction and TF–hub gene network diagram, it can be observed that hub genes, miRNAs and TFs are the key nodes with the highest degree value. CCND1 has been shown to be involved in the pathogenesis of obesity [189], but this gene might be novel target for GDM. Other studies which shown that FOXO1 [190], hsa-mir-1207-5p [191], hsa-mir-4651 [191], hsa-mir-222-3p [192] and E2F1 [193] are involved in GDM evolution. Hsa-let-7d-5p [194], hsa-mir-29c-3p [195] and SRY (sex-determining region Y) [196] have been reported to be associated with type 2 diabetes mellitus, but these genes might be novel target for GDM. Expression of hsa-mir-663a [197] and TBX3 [198] were consistent in cardiovascular diseases, but these genes might be novel target for GDM. Several reports have demonstrated that RUNX1 [199] and YAP1 [200] have active roles in preeclampsia, but these genes might be novel target for GDM. UBE2D3, PAK2, DDB1, DVL3, PSMC4, hsa-mir-6127, hsa-mir-8063, hsa-mir-329-3p, hsa-mir-410-5p, HCFC1, ZFX (zinc finger protein, X-linked), SPI1, MYBL2 and SUZ12 were the novel biomarkers for the progression of GDM.

The molecule GLY, MLR obtained a good -CDOCKER interaction energy with 5NJX, 3FNI and 3Q4Z the -CDOCKER interaction energy of GLY is 41.37, 59.92, 41.44 and for MLR is 40.68, 87.65, 43.47 with 5NJX, 3FNI and 3Q4Z, respectively. The two molecules such as ALO and MAL its interaction with amino acids are 2′ hydroxyl group formed hydrogen bond interaction with ASP-89 and 3′, 4′ hydroxyl groups formed hydrogen bond interaction with GLU-86. Following 6′ hydroxyl group formed hydrogen bond interaction with LYS-61. The C-13 hydroxyl formed hydrogen bond interaction with ASP-389 and ring C electrons formed π–π t-shaped interactions with HIS-63 and π–alkyl interaction with LYS-388. Ring A electrons formed π–carbon interaction with LYS-388 and LYS61, respectively. The ring C electrons and 4′ hydroxyl group of molecule MLR formed sulphur oxygen interaction with MET-344 and ring C electrons formed π–alkyl interaction with LEU-396. The ring A 5 and 6 hydroxyl group formed hydrogen bond interaction with ASP-354 and LYS-538. Ring D 3″ and 6″ hydroxyl group formed hydrogen bond interaction with ASP-393 and GLY-277, 3″ hydroxyl group formed pi-alkyl interaction with Mg ion. Ring D 5″ hydroxyl group formed hydrogen bond interaction with ARG-299. Ring E 6′″ alkyl hydroxyl formed Carbon hydrogen interaction with LYS-391 and ring E oxygen, 3′″ hydroxyl group and 6′″ alkyl hydroxyl formed π–alkyl interaction with Mg ions, respectively.

We conducted a comprehensive bioinformatics analysis on transcription profiling of GDM. Hub genes and pathways were identified to provide more detailed molecular mechanisms for the process of GDM and shed light on potential therapeutic targets. Nevertheless, further experiments are needed to further validate the identified hub genes and pathways.

## Data Availability

The datasets supporting the conclusions of this article are available in the ArrayExpress database (https://www.ebi.ac.uk/arrayexpress/) repository [(E-MTAB-6418) https://www.ebi.ac.uk/arrayexpress/experiments/E-MTAB-6418/?array=A-MEXP-2072].

## Abbreviations

adj. P. val: adjusted P value
AUC: area under the curve
BP: biological process
CC: cellular component
CEBPB: CCAAT enhancer binding protein beta
CRH: corticotropin releasing hormone
DEG: differentially expressed gene
EGFR: epidermal growth factor receptor
GDM: gestational diabetes mellitus
GEO: Gene Expression Omnibus
GO: Gene Ontology
HSP90AA1: heat shock protein 90 alpha family Class A member 1
MF: molecular function
PAK1: p21 (RAC1) activated kinase 1
